# Simulation of electrochemical properties of naturally occurring quinones

**DOI:** 10.1038/s41598-020-70522-z

**Published:** 2020-08-11

**Authors:** Sebastian Birkedal Kristensen, Tanja van Mourik, Tobias Bruun Pedersen, Jens Laurids Sørensen, Jens Muff

**Affiliations:** 1 Department of Chemistry and Bioscience, Section of Chemical Engineering, Aalborg University, Niels Bohrs Vej 8, 6700 Esbjerg, Denmark; 2 School of Chemistry, University of St. Andrews, North Haugh, St. Andrews, Fife, KY16 9ST Scotland, UK

**Keywords:** Biochemistry, Computational biology and bioinformatics

## Abstract

Quinones are produced in organisms and are utilized as electron transfer agents, pigments and in defence mechanisms. Furthermore, naturally occurring quinones can also be cytotoxins with antibacterial properties. These properties can be linked to their redox properties. Recent studies have also shown that quinones can be utilized in flow battery technology, though naturally occurring quinones have not yet been investigated. Here, we have analyzed the properties of 990 different quinones of various biological sources through a computation approach to determine their standard reduction potentials and aqueous solubility. The screening was performed using the PBE functional and the 6-31G** basis set, providing a distribution of reduction potentials of the naturally occurring quinones varying from − 1.4 V to 1.5 V vs. the standard hydrogen electrode. The solvation energy for each quinone, which indicates the solubility in aqueous solution, was calculated at the same level. A large distribution of solubilities was obtained, containing both molecules that show tendencies of good solubilities and molecules that do not. The solubilities are dependent on the nature of the side groups and the size of the molecules. Our study shows that the group containing the quinones of fungal origin, which is also the largest of the groups considered, has the largest antimicrobial and electrochemical potential, when considering the distribution of reduction potentials for the compounds.

## Introduction

Quinones are organic molecules found in nature in a variety of different types with different properties based on chemical and aromatic ring structure, side-chain groups, etc. Quinones in nature fall into the category of secondary metabolites, and are found in flowering plants, fungi, bacteria, algae and in some amounts in animals^1,2^. Common to all of them is the aromatic di-one or di-ketone system, which can be placed both in para or ortho positions. Quinones are often described as derivatives from oxidization of hydroquinones or polyphenols^3,4^. Naturally occurring quinones include aromatic ring structures ranging from the common 1-ring structures named benzoquinones (BQ), 2-ring structures named naphtoquinones (NQ) and 3-ring structures named anthraquinones (AQ) as well as more complex polyquinones^1–4^. In most eukaryote cells plastoquinone and ubiquinone conduct electron transport in the oxygenic photosynthesis and the aerobic respiratory chain, respectively ^2,5–7^. The function of quinones in living organisms is primarily due to their ability to undergo reversible 2${e}^{-}$ redox reactions that through complex reaction mechanisms protect the cells against free radicals and other potential harmful oxidants.

Quinones have during the past two decades been investigated in detail in plants, due to their medicinal properties in e.g. rhubarb (*Rheum* spp.^3,8–12^). The plant itself has been used in Chinese medicine since the Han dynasty^9^. It has also been shown that the AQs of rhubarb can inhibit bacterial growth, treat cancer, and inhibit protein misfolding and aggregation, which can be useful in the treatment of diabetes^3,9,10^. When challenged by microorganisms, rhubarb uses the secondary metabolites as a defence mechanism and it has been shown that the production of quinones increases when the plant is exposed to elicitor-active chemicals^13^. The compounds found in rhubarb can also be utilized as dyes and pigments as numerous quinones naturally absorb light in the visible range of the electromagnetic spectrum due to the presence of conjugated double bonds in the structure. The pigments and dyes can furthermore act as antibacterial agents when used as treatment of woolen thread^14^. These antimicrobial properties have been linked to the plant’s redox biochemistry, and consequently to the quinones, as they often acts as electron transfer agents^11,15^.

The occurrence of quinones in bacteria is also well known and widely described. In phototrophic bacteria the quinones perform different functions as electron transfer agents in respiratory and photosynthetic processes. Menaquinones and ubiquinones are constituents of bacterial plasma membranes and represent an important role in electron transfer and possibly also in phosphorylation^2,6,7^. It is well known that fungi produce a plethora of secondary metabolites with a variety of functions, used in survival and communication in natural habitats. Among the most famous antibacterial agents found in fungi is penicillin, found in the *Penicillium* and *Aspergillus* species^16^. Some of the secondary metabolites from fungi contain quinone structures in different variations^2,17–20^. The complex mechanism of cytotoxic properties of the quinone metabolites can be attributed to the interaction with the mitochondrial nicotinamide adenine dinucleotide (NAD) and NAD phosphate (NADP) dependent flavin enzymes, where the quinone undergoes a reduction, and hereby produces semiquinone radicals. These can react with oxygen and hereby create superoxides which, together with the semiquinone radicals, can damage DNA, RNA and other macromolecules^20–22^. Furthermore, the oxidative stress can induce apoptosis, which has been observed in several organisms and cancer cells^23,24^. Similarly to plants, the defence mechanism in fungi can therefore also be attributed to the redox behavior of these compounds. However, due to versatile structure and characteristics of biologically produced quinones, the cytotoxic mechanism for each quinone is far from fully determined. An example of determined cytotoxic properties can be seen in the *Fusarium* species, that produces, among others, the quinone aurofusarin. This compound has shown inhibitory effects towards different types of bacteria including *Lactobacillus* and *Bifidobacterium*^25^*.* Understanding the redox behavior of quinones is a key component in understanding how these compounds work antimicrobially, for example in dyes of various sorts, thereby expanding our knowledge of the biochemistry of organisms, especially with respect to the medical use of quinones. The reduction potential can be directly linked to the microbial properties of the quinone structure^20,25^. The solubility of the compounds is also an important property to take into account, as a quinone with a higher solubility can travel easier around cells, through cell walls etc., and can be utilized to a larger extent than a compound with a lower solubility. Due to the redox properties of quinones, the use of quinones as electrolytes in flow batteries has in recent years become an area of interest, where quinones have shown potential as substitutes for metals such as vanadium^26–33^. In this field of research the focus has so far been on synthetically prepared quinones, and changing sidechain groups for improvement of reduction potentials and solubility has been the main emphasis^26–33^. The naturally orccuring quinones however present the same kind of redox activity. This paper is thereby also a screening for the extent of reduction potentials found in natural occurring quinones and their solubility in aqueous solutions for potential uses in flow battery technology. Previous reports have determined the electrochemical potential of artificial or chemically synthesized quinones through various computational methods^26,27,34–37^. In this paper, we have used a similar approach on 990 natural occurring quinones, primarily derived from bacteria and fungi.

## Results and discussion

### Distribution of E^0^ of naturally occurring quinones

**Figure 1:**
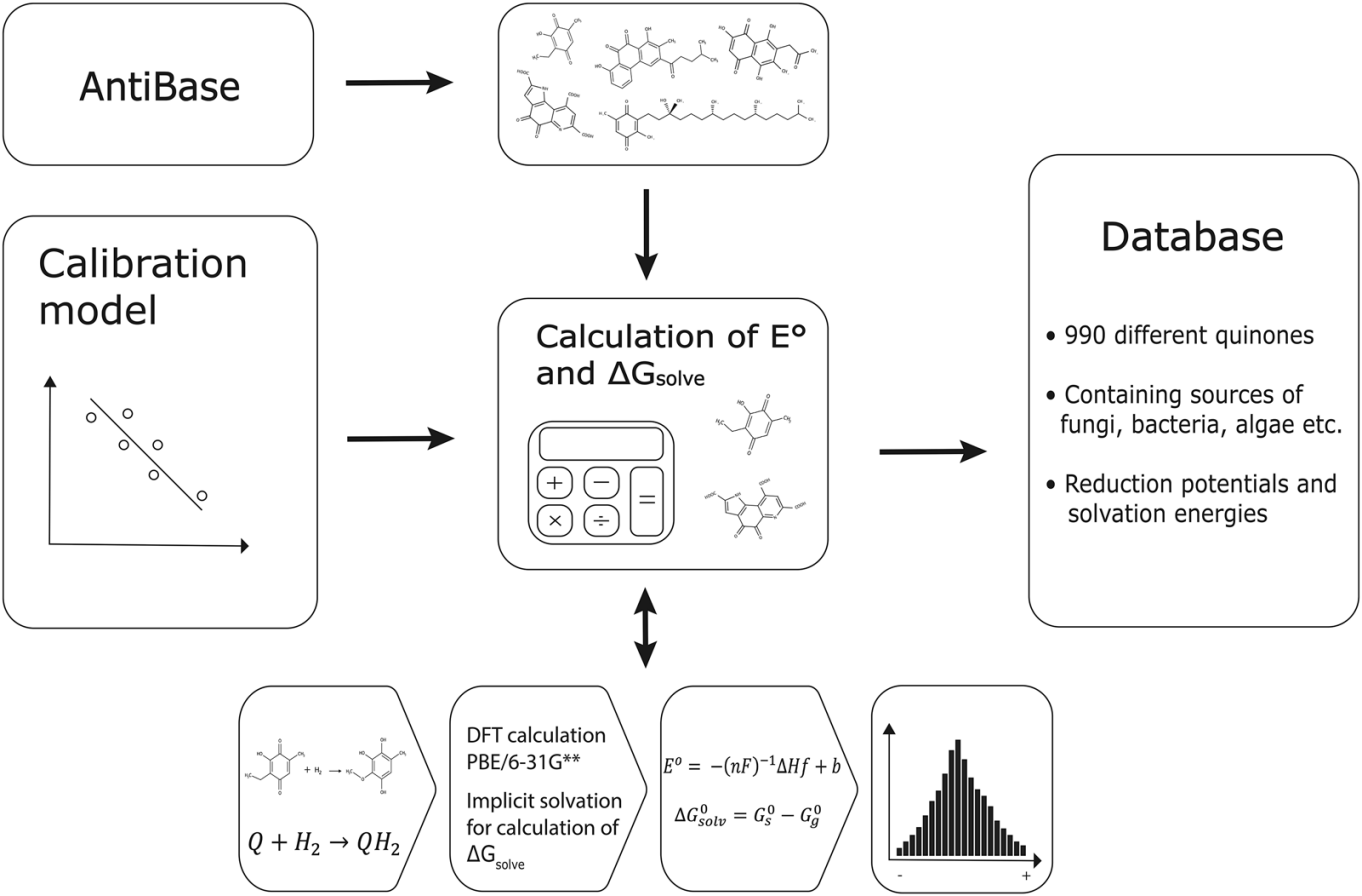
Flow diagram of the computational procedure employed in the study.

**Figure 2:**
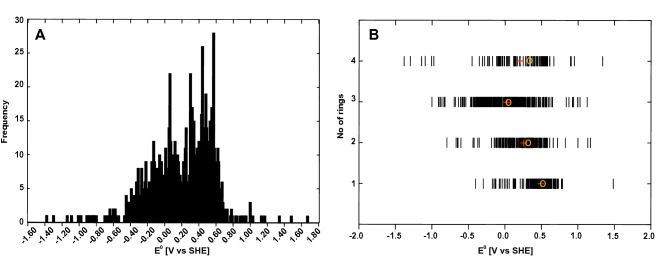
(**A**) histogram of distribution of predicted redox potentials. (**B**) distribution of redox potentials based on the number of aromatic rings within the compounds.

In order to predict the redox potentials (${E}^{0}$) of known quinones of biological origin, we followed the pipeline outlined in Fig. 1. The dataset consisted of quinones from various sources, including 221 quinones from bacteria, 358 derived from fungi and 425 from “other” sources such as yeast, algae and plants. All was analyzed using density functional theory (DFT). Some of the quinones (e.g. herbarin, which is produced both in yeast^38^, and in fungi^39^) are produced in several different sources and are included twice, thereby making the sum of the above listed numbers exceed 990 (Supplementary Table 1). The distribution of the predicted ${E}^{0}$ of the naturally occurring quinones is visualized in Fig. 2A. The histogram shows that the majority of the predicted ${E}^{0}$ falls between − 0.4 V versus SHE and 0.7 V versus SHE, with slight indication towards a normal distribution of the potentials. The predicted values stretch from the most negative value at − 1.382 V versus SHE to the most positive value at 1.485 V versus SHE. The skewness of the distribution, − 0.6179, indicates that the distribution is slightly skewed towards the left (Supplemental Fig. 1). Dividing the quinones in groups according to the number of rings, shows that single-ring compounds (BQs) are located primarily around a median value of 0.4970 V versus SHE, although two compounds are placed at the positive extreme (Fig. 2B). The NQs (209 structures) display a wider distribution at both ends compared to the BQs, with a median value of 0.2870 V versus SHE and a mean value of 0.2340 V versus SHE. The largest group of quinone structures contains 411 different AQ structures. The mean and median of the AQs are 0.0024 V versus SHE and 0.0119 V versus SHE, respectively, the lowest of the four groups. The BQs, 276 structures, have the most positive mean value of 0.4788 V versus SHE. The structures with four or more rings form the smallest group, with 94 members. These have the largest diversity in potentials, with both positive and negative values. The mean is at the positive side of the diagram, 0.1803 V versus SHE, and the median is 0.3040 V versus SHE.

Closer examination of the analysed quinones shows that the seven quinones with the most positive potentials are all produced from various species of fungi, mostly from the species *Penicillium* and *Fusarium* (Table 1)*.* With a value of 1.485 V versus SHE, the compound with the most positive redox potential is tridentoquinone, a molecule produced by *Suillus tridentinus*^40^. Tridentoquinone is a benzoquinone, and the solubility descriptor indicates that it has a rather low relative solubility in water, which could be due to the large carbon chain in the compound, including several methyl groups, and double bonds. In general the seven most positive quinones show low numbers of electron donating groups (EDGs), such as –OH. These groups have been described as lowering the reduction potential when present^34^, and they show a similar tendency in this study. The positive list also includes compounds that are smaller than the negative compounds, displaying less aromacity leading to higher reduction potentials.

**Table 1:**
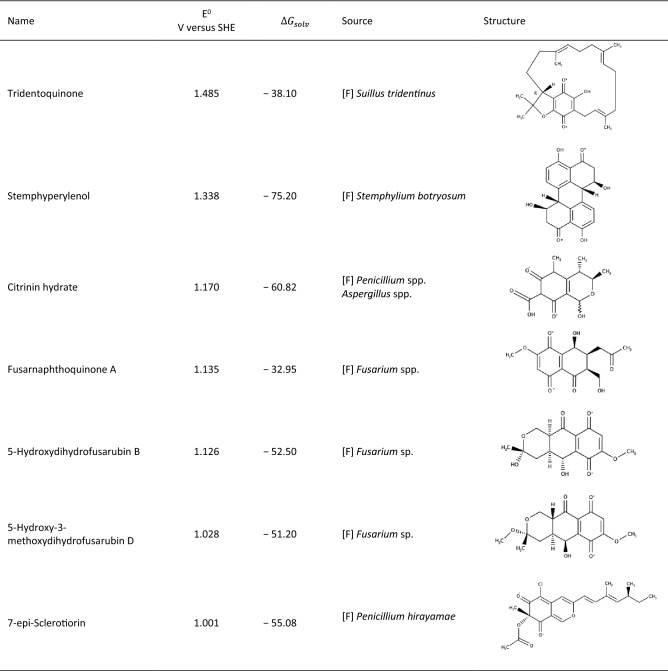
Properties of the seven quinones with the most negative potentials.

[F]-fungi, [B]-bacteria, * indicates the point of reduction of the reactant.

Whereas the quinones with the most positive potentials predominately originated from fungi and are relatively small and simple compounds, the quinones with the most negative potentials are larger and more complex quinone structures (Table 2). The compound with the most negative redox potential (− 1.382 V vs. SHE) is pradimicin M, which is produced in a mutant of the bacterium *Actinomadura hibisca.* The compound contains five rings and is therefore within the group of ≥ 4 ring structures. The results shows that, even though the compounds are larger than the quinones with predicted positive potentials, they have higher solubility descriptors, which can be linked to the presence of seven EDGs leading to an enhancement of the polarity of the compound. The number of EDGs present in the seven most negative compounds is significantly higher than in the compounds presenting positive reduction potentials, in agreement with findings in similar studies^34^. It is furthermore interesting that the structures of stemphyltoxin, altertoxin I, altertoxin II and stemphyltoxin IV are so similar; they only differ in the placement of the side chain groups. The structure of stemphyperylenol (Table 1), which has a positive potential, is also similar to the negative-potential compounds discussed above; however, the aromatic rings in this compound are placed opposite each other, and the compound contains a larger number of polar side chain groups. This may explain the large difference in reduction potential. The compound 7-epi-sclerotiorin (Table 1) , which has a simulated reduction potential of 1.001 V versus SHE, contains no EDGs, but a –Cl group which tends to make the reduction potential positive according to the study of Er et al.^34^, in agreement with findings in this manuscript. The full list of quinones and their predicted potentials is provided in the supporting information.

**Table 2:**
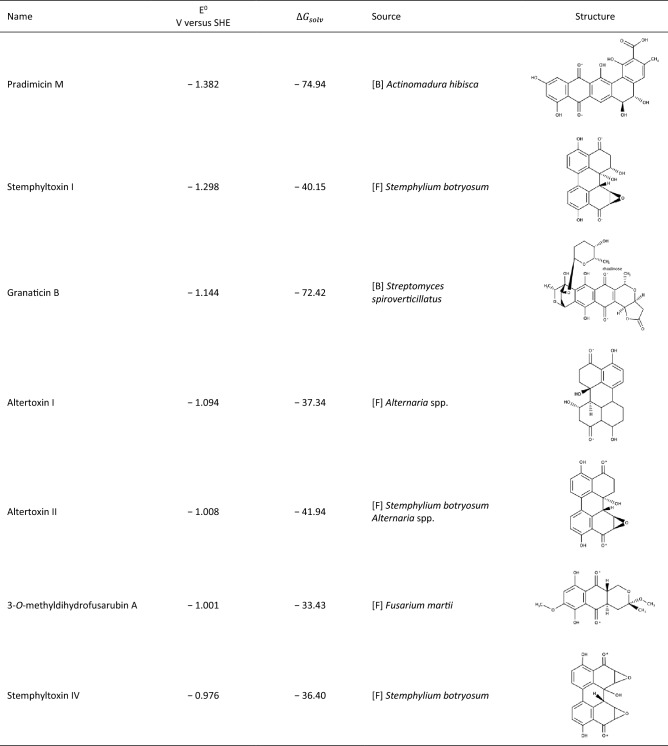
Properties of the seven quinones with the most negative potentials

[F]-fungi, [B]-bacteria, * indicates the point of reduction of the reactant.

### Distribution of E^0^ according to biological origin

**Figure 3:**
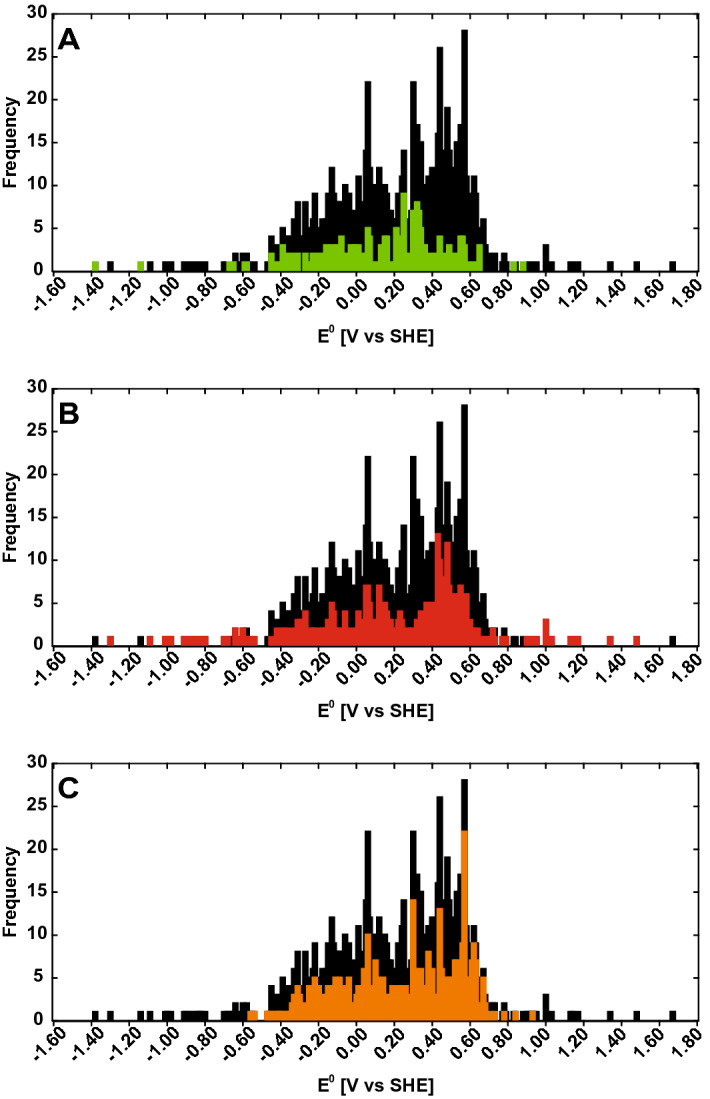
(**A**) distribution of reduction potentials for quinones produced in bacteria, (**B**) distribution of reduction potential for quinones produced in fungi, (**C**) distribution of reduction potentials for quinones produced in "other"(plants, algea , animals etc.). The distribution of the reduction potentials for all quinones is shown in black.

To get an overview of the electrochemical properties of the quinones according to their biological origin we divided the dataset into three groups: bacteria, fungi and others (plants, algea, animals etc.; Fig. 3 and Supplementary Fig. 2). The majority of the reduction potentials of the bacterial quinones is located in the middle of the histogram (Fig. 3A and Supplemental Fig. 3). The reduction potentials of the fungal quinones are more widely distributed compared to those in the other two groups (Fig. 3B and Supplemental Fig. 4). Thus, the fungi-produced compounds constitute the most varied group. The reduction potentials of the collective group of “other” quinones is centered around the middle values of the histogram with no extreme values (Fig. 3C and Supplemental Fig. 5).

**Figure 4:**
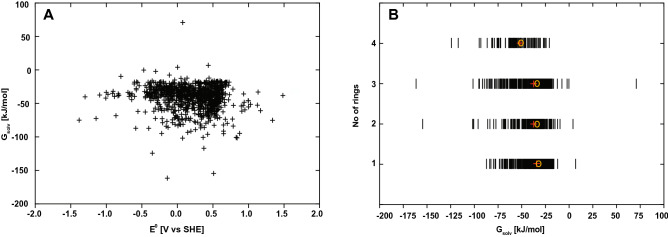
(**A**) Redox-solubility maps of all predicted quinone structures. (**B**) breakdown of solubility according to the number of rings.

**Figure 5:**
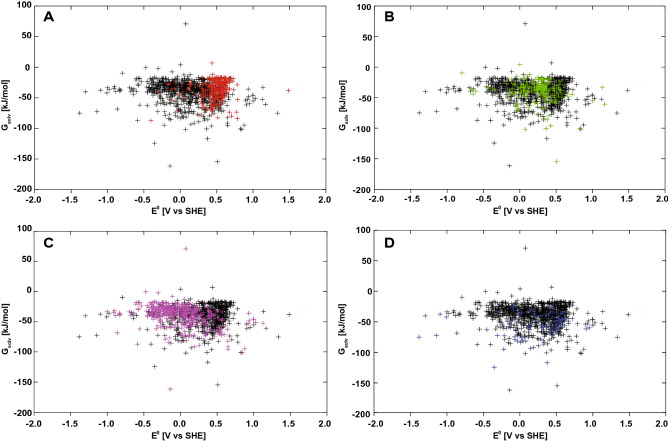
Distribution of quinones based on predicted reduction potential and solubility. (**A**) BQs highlighted with red, (**B**) NQs highlighted with green, (**C**) AQs illustrated with purple, (**D**) ≥ 4 rings is visualized with blue. The black marked points correspond to the values for the other quinone groups.

### Correlation of solubility and electrochemical potential

The calculated solubility of the quinones ranges between 0–100 kJ/mol, with only a few compounds outside this range (Fig. 4A). A breakdown of the ${\Delta G}_{solv}$ descriptors according to the number of rings in the quinones shows only marginal differences (Fig. 4B). The ≥ 4 group has the largest mean value of − 54.1543 kJ/mol and median of − 53.2041 kJ/mol, whereas the three other groups do not show any significant differences in solubility. This disagrees with the expectation that larger organic molecules have a lower solubility due to their larger non-polar surface. Apparently, the solubility of the quinones studied here is more dependent on the polar side chain groups than on their number of rings.

A breakdown into the BQs, NQs, AQs and ≥ 4 ring groups categories of the distribution of the solubility as a function of the redox potentials is shown in Fig. 5. The solubilities of the BQs tend to be at the positive side of the distribution, whereas the solubilities of the NQs are more centrally located (Fig. 5A,B). The solubilities of the AQs are more distributed towards the negative side compared to the BQs and NQs, which indicates a higher solubility of these compounds (Fig. 5C). The distribution of the BQs, NQs and AQs appears to indicate that the more rings the compound contains the lower is the redox potential, which corresponds to the correlation between higher aromacity and lower reduction potential, discussed above. However, the ${E}^{0}$ values of the ≥ 4 ring structure group are much more varied than those of the BQs, NQs and AQs, which is likely due to the larger variety in overall chemical structure within this group. These compounds have on average a slightly more negative descriptor of solubility, indicating that they have a slightly higher solubility compared to the other groups of quinones (Fig. 5D).

**Figure 6:**
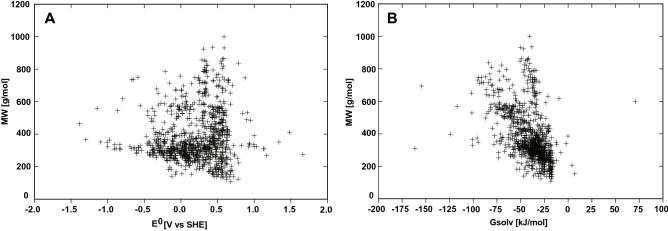
(**A**) Molecular weight versus predicted reduction potential distribution. (**B**) Molecular weight versus G_solv_ distribution.

The size of the compounds is usually a significant indicator for the solubility, as larger compounds tend to be less soluble than smaller compounds. In Fig. 6, molecular weight (MW) is presented versus reduction potential and solubility descriptor ${\Delta G}_{solv}$ respectively. The largest compound found in the database, ubiquinone Q12, has a MW of 999.58 g/mol, , and shows a reduction potential of 0.59 V and a ${\Delta G}_{solv}$ of − 40.36 kJ/mol. The ${\Delta G}_{solv}$ and ${E}^{0}$ of this compound are not placed at the extreme ends of the dataset, but placed more positive in both of the parameters. There is not a clear correlation between MW and reduction potential (Fig. 6A); however, the largest distribution of reduction potentials is observed to occur for compounds with smaller MW. Furthermore, a weak tendency towards larger compounds having a more negative ${\Delta G}_{solv}$ can be observed, which corresponds to the general hypothesis; however, the plot shows two slightly correlating tendencies, one which is going vertical, meaning no cooralation (Fig. 6B). The second correlation indicates that the higher MW, the lower the ${\Delta G}_{solv}$ is. The correlation is not significant, and thus, it cannot be concluded that the molecular weight directly influences the ${E}^{0}$ or ${\Delta G}_{solv}$, the occurrence of polar groups appears to have a larger impact on the ${E}^{0}$ or ${\Delta G}_{solv}$ than the MW of the compounds.

## Experimental

A flow diagram of the workflow used in the project is presented in Fig. 1. The reaction of the naturally occurring quinones to hydroquinones was treated as a single step: a rapid, two-electron and two-proton transfer as previously described^26,27,34–37^. The naturally occurring quinones that have been isolated as hydroquinones were initially prepared into oxidized versions, and only two of the hydroxyl or ketone groups were used for the simulation. If a compound presented several redox sites, only one of these was taken into account. The redox site used for the simulation has been marked with a * in Tables 1 and 2. The quinones that have been changed from the isolated form are marked with either a (1) or (2), according to the number of sites that have been changed prior to simulation, in the complete list of simulated naturally occurring quinones (Supplemental Table 1). The reaction scheme applied in the screening used the thermodynamic cycle shown in Supplemental Fig. 6.

### Database creation

A local database was compiled from AntiBase 2012^41^. The quinones were obtained through multiple searches in AntiBase which contains 41,000 recorded compounds. An initial search identified compounds containing “quinone” as structure name tag, which yielded 767 hits. A search of specific quinones was also conducted; this yielded approximately 40 hits. Lastly, a search for “quinones” in the “properties” search field was conducted; this search yielded 425 quinone structures. A proportion of the compounds appeared in more than one of the searches; these compounds were noted and removed so they only appear once in the database. This resulted in a database containing 990 different quinone structures originating from various sources i.e. fungi, bacteria, plants and animals.

### Development of the calibration model

To calculate the electrochemical properties of the 990 identified quinones we developed a method based on earlier published research on the screening of quinones^26,30,34,42^, which also employed DFT. All DFT calculations in the current project were carried out using the Gaussian 09 software^43^. First, results obtained by the Perdew-Burke-Ernzerhof (PBE^44^) Results obtained with the GGA (generalized gradient approximation) functional PBE and the more computationally demanding meta-hybrid M06-2X functional^45^, both coupled with the split-valence 6-31G** basis set, were compared. To calibrate the model, six basic quinones were chosen as representatives of BQs, NQs and AQs. The calibration model employed experimental redox potentials ($${E}_{exp}^{0})$$ of these compounds and the change of enthalpies of formation $$\left(\Delta {H}_{f}\right)$$ of the quinone redox reaction calculated using the two different functionals^46,47^. A line was fitted though the data points using least-squares fitting (Supplemental Fig. 7). The calibration model is based on the modified Nernst equation:

1 $$E^{0} = - (nF)^{ - 1} \Delta H_{f} + b$$

where *n* is the number of electrons transferred (2), *F* is the Faraday constant and *b* is the y-axis intercept obtained from the calibration model.

The PBE functional yielded slightly better correlation, ${R}^{2}$ = 0.9827 (Supplemental Fig. 7A), compared to the more computationally expensive M06-2X functional with ${R}^{2}$ of 0.9767 (Supplemental Fig. 7B). Therefore, the PBE/6-31G** data is applied to Eq. 1 to obtain the calibration model used in this work (Eq. 2).

2 $${E}^{0}= -0.4629\Delta {H}_{f}+0.0572$$

This equation enabled us to correlate the calculated change in gas-phase enthalpy of formation $(\Delta {H}_{f}$) at 0 K to the experimental redox potential for the reaction $Q/Q{H}_{2}$.

### Computational workflow

The structures in our database were converted from 2D structures into 3D structures using the Avogadro software^48^, visualized with GaussView^49^, and Gaussian input files were then created for both the reduced and oxidized form of the quinone. The calibration model (Eq. 2) was used to calculate the reduction potentials based on the computed energies, using the PBE/6-31G** functional/basis set combination. For solubility prediction in aqueous media, we used $\Delta {G}_{solv}$ as a descriptor calculated using PBE/6-31G**, for the oxidized forms of the quinones, as these are assumed to have a lower solubility compared to their reduced forms. The $\Delta {G}_{solv}$ was calculated as the difference between the total Gibbs energy of the oxidized quinone in aqueous solution ($\Delta {G}_{aq})$, calculated using the Polarizable Continuum Model (PCM) implicit solvation model, using the integral equation formalism variant (IEFPCM), with water as the solvent, using the dielectric constant of water (ε = 78.3553)^50,51^, and the total Gibbs energy of the oxidized quinone in gaseous phase $(\Delta {G}_{gas})$. This infers that the more negative the value of $\Delta {G}_{solv}$, the higher the solubility of the quinone.

## Supplementary information

Supplementary Information.
