# Supplementary material for: Simulation of electrochemical properties of naturally occurring quinones
Source: Sci Rep. 2020 Aug 11;10:13571. doi: 10.1038/s41598-020-70522-z (PMC7419317; doi:10.1038/s41598-020-70522-z)
Supplement: Supplementary file 1 — Supplementary Information. [file 41598_2020_70522_MOESM1_ESM.pdf]

Simulation of electrochemical properties of bio-quinones

Sebastian Birkedal Kristensen[1], Tanja van Mourik[2], Tobias Bruun Pedersen[1], Jens Laurids Sørensen[1,*], Jens Muff[1].

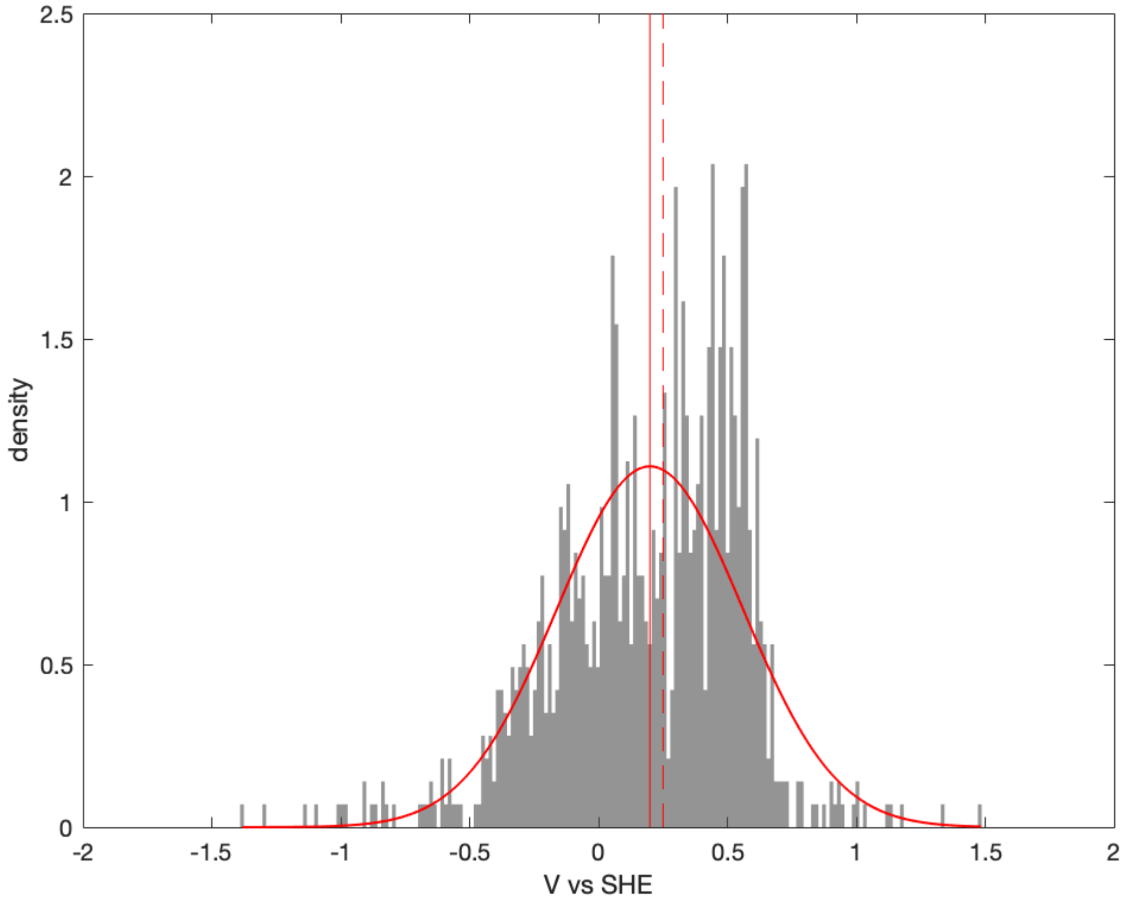

Supplemental Figure 1. Normal distribution curve (red line) for all quinones in the dataset. The Normal distribution curve is here fitted to the total distribution, mean and median indicated by vertical redline and red dashed line. The distribution is represented as density.

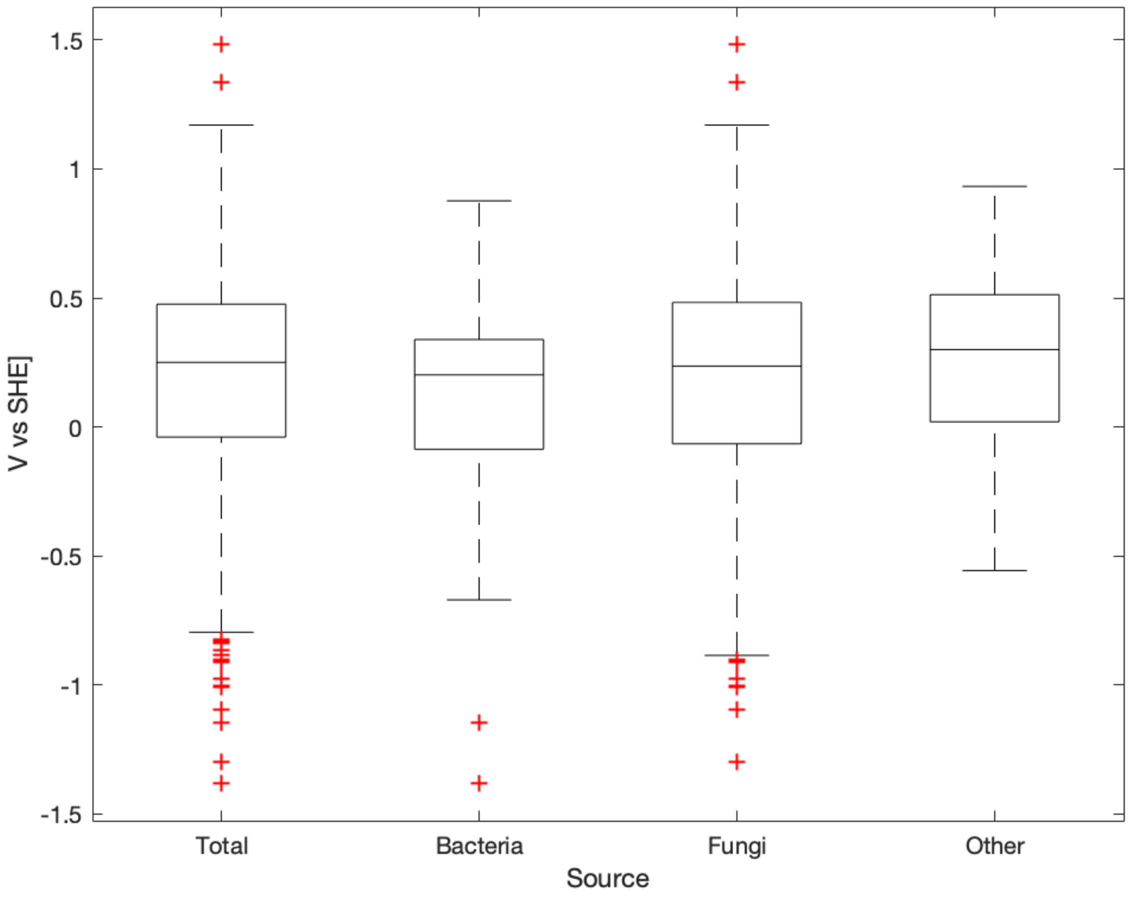

Supplemental Figure 2. Boxplot of distributions. Distributions illustrated as boxplots for the Total amount of quinones, the quinones produced in bacteria, fungi and "other" – which includes plants, algae etc. it is seen that the widest distribution in found in the compounds produced in fungi.

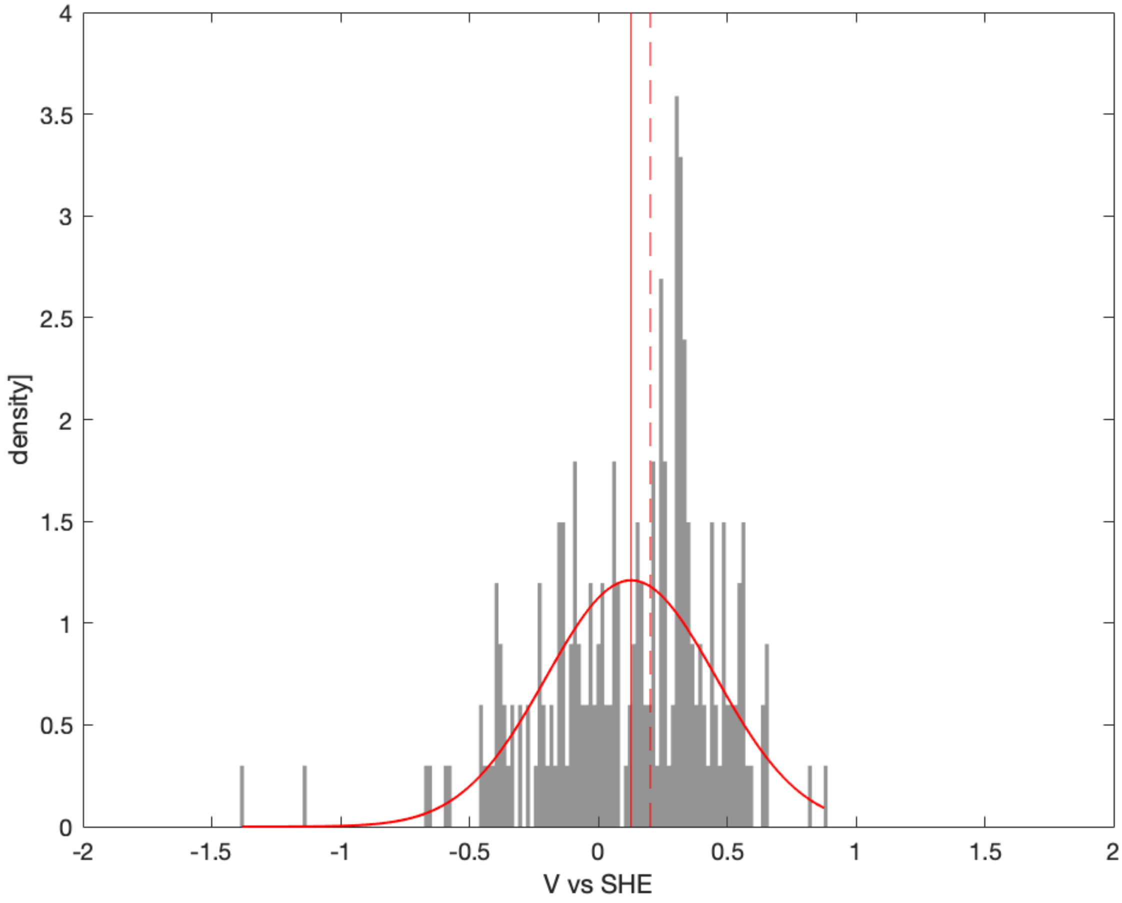

Supplemental Figure 3. The Normal distribution curve (red line) fitted to the distribution of quinones produced in bacteria. Mean and median indicated by vertical redline and red dashed line. The distribution is represented as density.

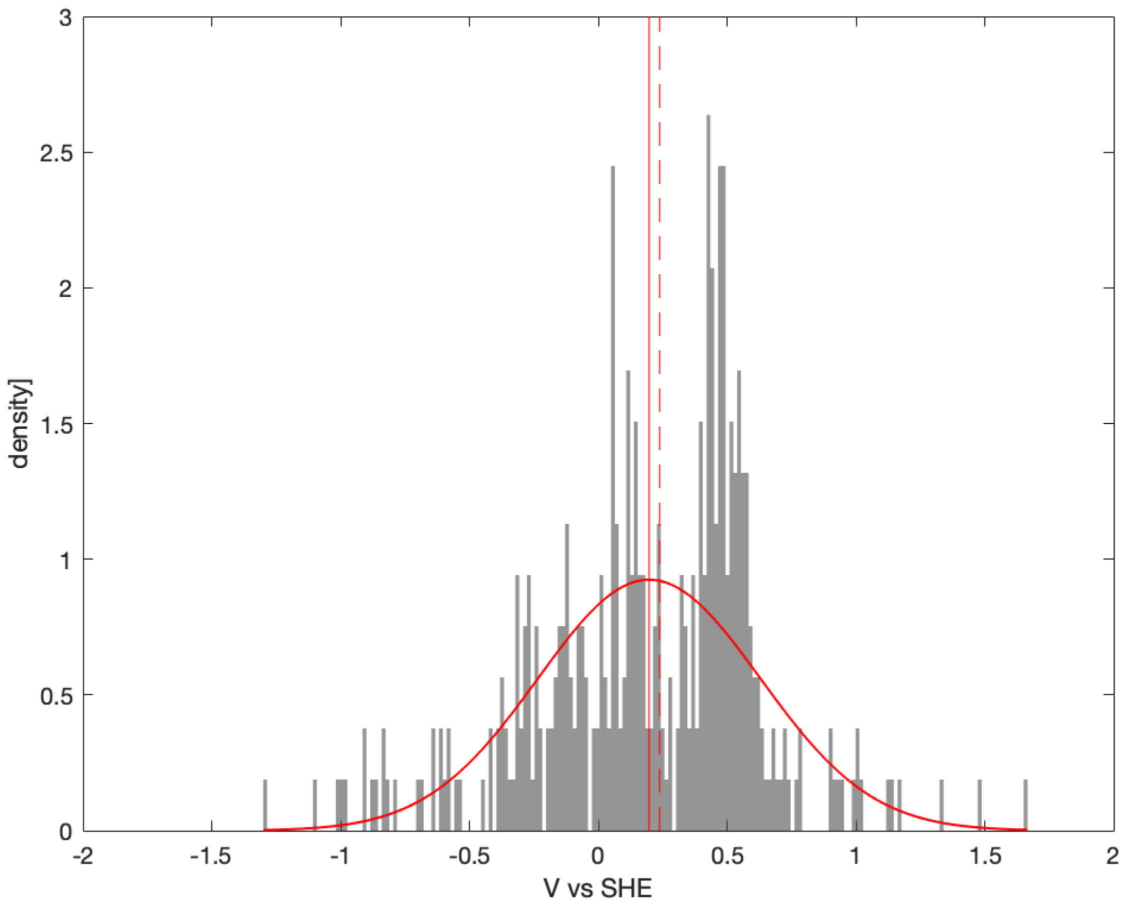

Supplemental Figure 4. The Normal distribution curve (red line) fitted to the distribution of compounds produced in fungi. Mean and median indicated by vertical redline and red dashed line. The distribution is represented as density.

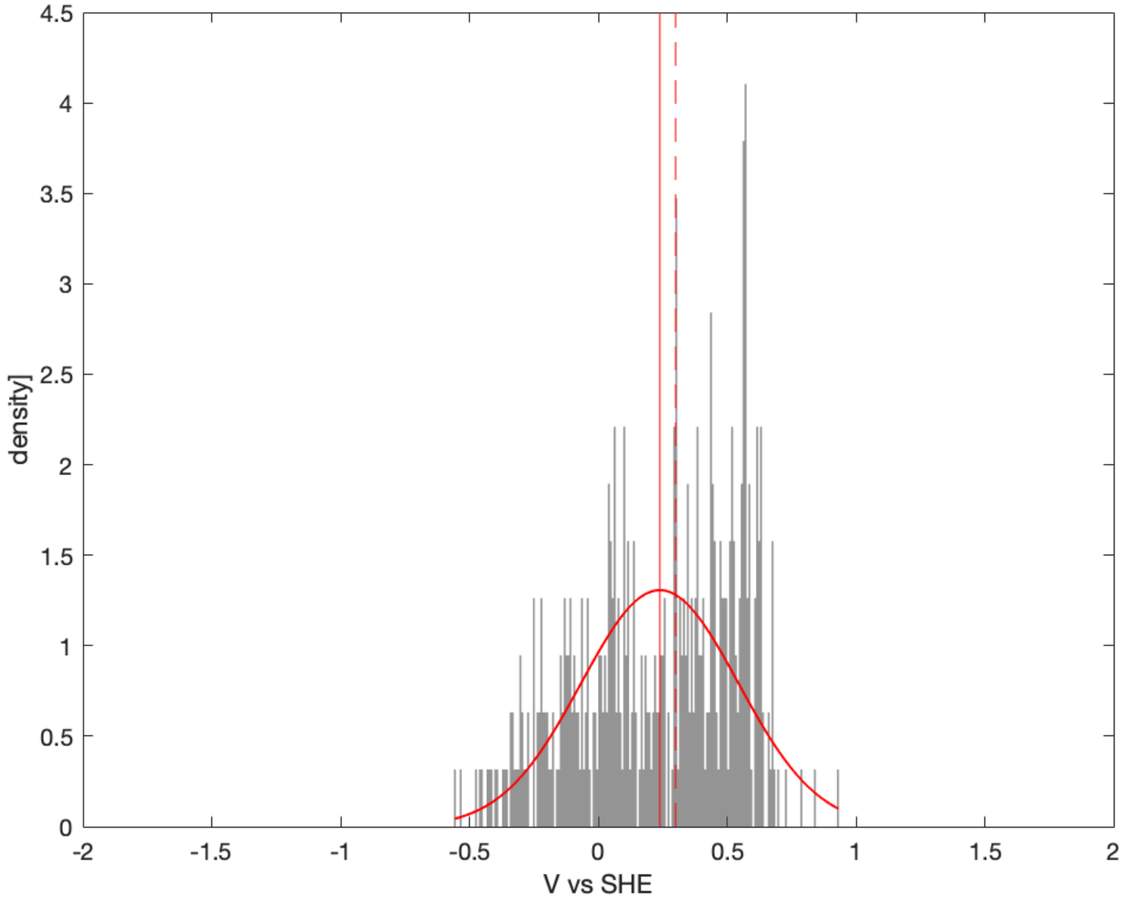

Supplemental Figure 5. The Normal distribution curve (red line) fitted to the distribution of compounds produced other organisms than bacteria and fungi (plants, algae etc.). Mean and median indicated by vertical redline and red dashed line. The distribution is represented as density.

$$Q_{(g)} \quad + \quad H_{2(g)} \quad \xrightarrow{\Delta H^{\circ}_{f(gas)}} \quad QH_{2(g)}$$

$$\downarrow \Delta G_{solv}(Q) \qquad \downarrow \Delta G_{solv}(H_2) \qquad \qquad \downarrow \Delta G_{solv}(QH_2)$$

$$Q_{(aq)} \quad + \quad H_{2(aq)} \quad \xrightarrow{\Delta H^{\circ}_{f(aq)}} \quad QH_{2(aq)}$$

Supplemental Figure 6. Thermodynamic cycle used in the current work. The reduction mechanism is using the formation energy in the gaseous phase. The ΔG_{solv} indicates the energy required to soloublize the oxidized quinone(Q), the hydrogen(H_2) and the fully reduced quinone(QH_2) in an aqueous solution. The solvation energy, ΔG_{solv}, is utulized to go from gaseous phase to aqueous. The bottom reaction is thereby in aqueous solution and thereby is the formation energy in aqueous solution, $\Delta H^{\circ}_{f}(aq)$, found, and used for calculation E^0.

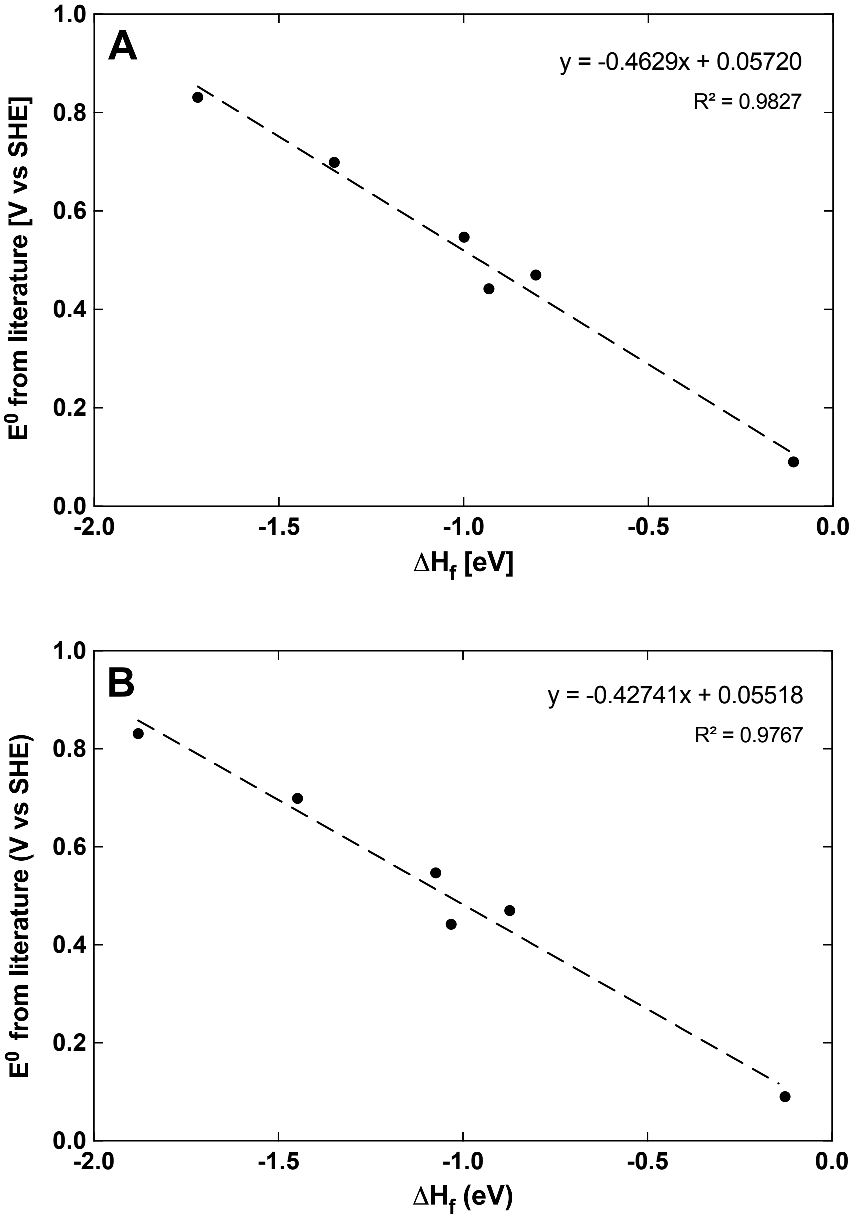

Supplemental Figure 7 - Calibration model for ΔH_f and experimental E^0. Linear relationship between calculated Formation energies, ΔH_f, and experimental reduction potentials, E^0, found in the literature using (**A**) Perdew-Burke-Ernzerhof and (**B**) meta-hybrid M06-2X algorithms. The six black dots represent six quinones, from left to right on the figure: 1.2-BQ, 1.4-BQ, 1.2-NQ, 9.10-PQ, 1.4-NQ and 9.10-AQ.

Supplementary table 1. List of examined quinones collected from AntiBase with calculated redox potentials (E^0) and solvation energy (Gsolv).

Name	ID AntiBase	E [V vs SHE]	Gsolv	rings	Formular	Structure	Source	Sites changed to oxidized form
Tridentoquinone	15805	1.485	-38.097	1	C26 H34 O4	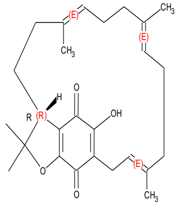	[F] Suillus tridentinus (Boletales)	
Stemphyperylenol	3527	1.338	-75.196	4	C20 H16 O6	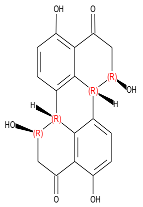	[F] Stemphylium botryosum var. lactucum	
Citrinin hydrate	8195	1.170	-60.823	2	C13 H16 O6	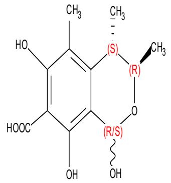	[F] Penicillium citrinum, P.implicatum, P.lividum, P.fellutanum, P. jenseni, P. citreo-viride, P.expansum, P.notatum, P. viridicatum, P.steckii, Aspergillus terreus, A.niveus, A.candidus,	2
Fusarnaphthoquinone A	38689	1.135	-32.945	2	C15 H18 O7	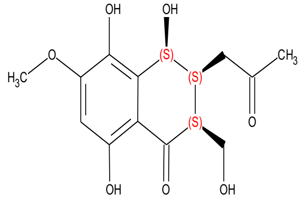	[F] Fusarium spp. PSU-F14 and PSU-F135	1
5-Hydroxydihydrofusarubin B	40219	1.126	-52.498	3	C15 H18 O7	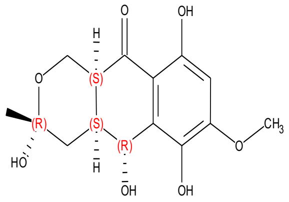	[F] endophytic Fusarium sp. BCC14842	1

5-Hydroxy-3-methoxydihydrofusarubin D	40050	1.028	-51.203	3	C16 H20 O7	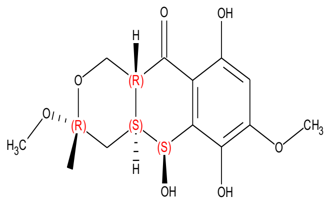	[F] endophytic Fusarium sp. BCC14842	1
7-epi-Sclerotiorin	9014	1.001	-55.079	2	C21 H23 Cl1 O5	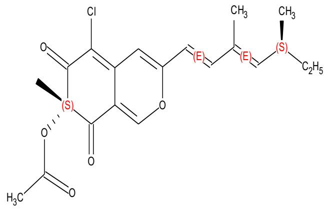	[F] Penicillium hirayamae	
5-Methoxydihydrofusarubin B	40048	0.999	-46.042	3	C16 H20 O7	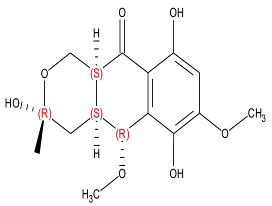	[F] endophytic Fusarium sp. BCC14842	2
3,5-Dimethoxydihydrofusarubin B	40049	0.996	-45.905	3	C17 H22 O7	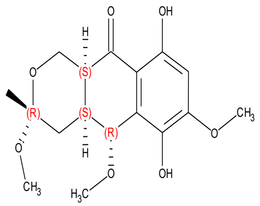	[F] endophytic Fusarium sp. BCC14842	2
Isocochlioquinone B	28896	0.950	-59.516	4	C28 H38 O7	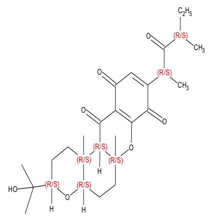	[F] plant pathogenic fungus Bipolaris cynodontis, [F] Drechslera dematioidea	

3,5-Dimethoxydihydrofusarubin D	40051	0.939	-48.218	3	C17 H22 O7	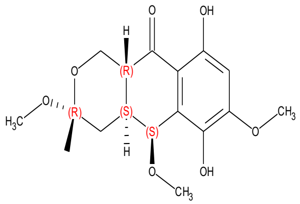	[F] endophytic Fusarium sp. BCC14842	2
Coleone-U-quinone	17191	0.932	-35.768	3	C20 H24 O5	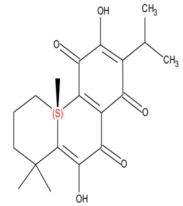		
Fusaranthraquinone	38688	0.924	-53.154	3	C17 H22 O6	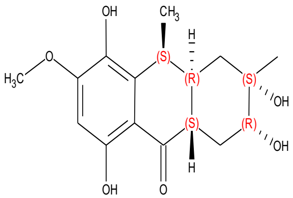	[F] Fusarium spp. PSU-F14 and PSU-F135	2
Isocochlioquinone A	20048	0.907	-61.496	4	C30 H44 O8	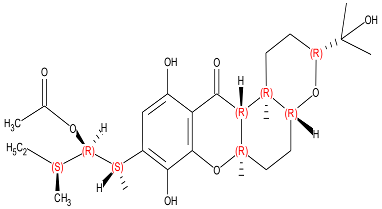	[F] plant pathogenic fungus Bipolaris cynodontis	2
Isocochlioquinone C	19643	0.898	-53.735	4	C28 H40 O7	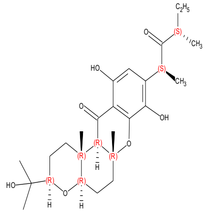	[F] plant pathogenic fungus Bipolaris cynodontis, [F] Drechslera dematioidea	2

Julichrome Q1,9	1915	0.876	-94.646	3	C38 H34 O16	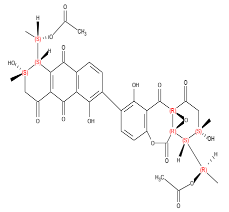	[B] Streptomyces shiodaensis	
Anhydroatrovirin-9,10-quinone	17502	0.840	######	3	C30 H24 O9	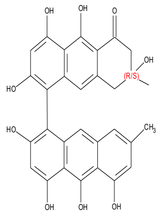		2
Methoxatin	15584	0.828	######	2	C14 H6 N2 O8	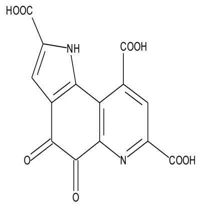	[B] Methylobacterium extorquens	
7-Hydroxy-8-methoxymalbranicin-5,6-quinone	30954	0.789	-52.894	1	C11 H12 O5	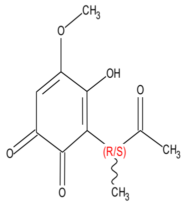	[F] Malbranchea cinnamomea HKI 286, HKI 296	
Amitenone	10116	0.785	-83.752	1	C53 H72 O8	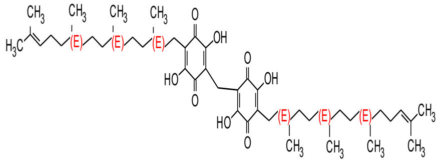		

490-Quinone	17763	0.781	-28.550	1	C6 H5 N1 O2	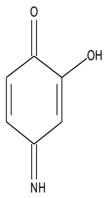	[F] Agaricus bisporus	1
g-L-Glutaminyl-3,4-benzoquinone	10414	0.775	-73.313	1	C11 H12 N2 O5	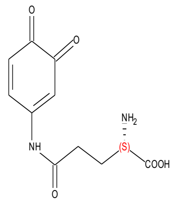	[F] Agaricus campestris	
Foeniculoxin	14965	0.733	-36.918	1	C16 H20 O4	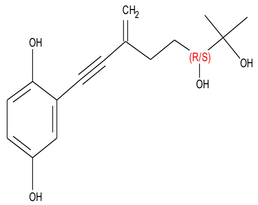	[F] Phomopsis foeniculi	2
2,6-Dibromo-1,4-benzoquinone	14816	0.728	-18.164	1	C6 H2 Br2 O2	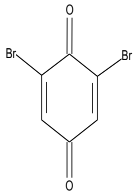	[An] Verongia aurea	
Scleroquinone	8653	0.719	-44.595	2	C18 H18 O6	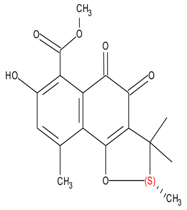	[F] Gremmeniella abietina	

Hydroxymethyl-1,4-benzoquinone	1818	0.719	-22.840	1	C7 H6 O3	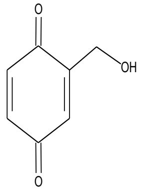	[F] Penicillium patulum	
Methylenediboviquinone-3,3	14888	0.702	-79.735	1	C43 H56 O8	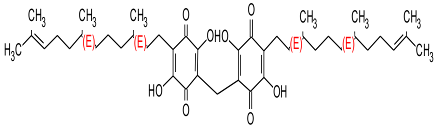	[F] Gomphidius rutilus	
2,5-Dichlorobenzoquinone	17560	0.698	-18.208	1	C6 H2 Cl2 O2	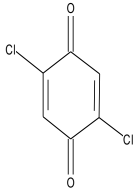	[synthetic]	
5-Bromotoluhydroquinone	42324	0.690	-18.413	1	C7 H7 Br1 O2	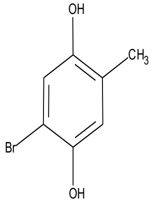	[F] marine-derived Dothideomycete sp. + NaBr	2
Chlorogentisylquinone	27252	0.683	-22.869	1	C7 H5 Cl1 O3	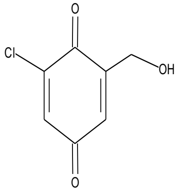	[F] marine fungus FOM-8100-cF7	

Benzoquinone	10174	0.680	-18.213	1	C6 H4 O2	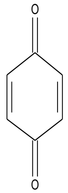	
2-Methoxy-3-methylbenzoquinone	9917	0.677	-24.969	1	C8 H8 O3	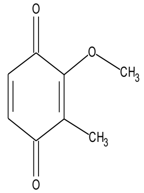	[An] millipede Floridobolus penneri
490-Quinone	10003	0.675	-73.502	1	C11 H12 N2 O7	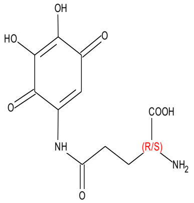	[F] Agaricus bisporus
2-Heptaprenyl-1,4-benzoquinone	14892	0.674	-34.936	1	C41 H60 O2	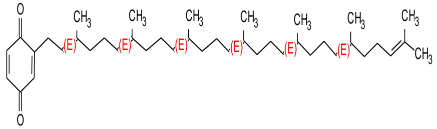	
Geranylbenzoquinone	14841	0.674	-21.548	1	C16 H20 O2	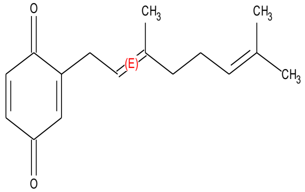	

2-Octaprenyl-1,4-benzoquinone	14893	0.674	-37.070	1	C46 H68 O2	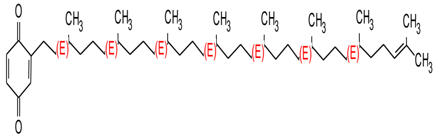	
g,g-Dimethylallyl-1,4-benzoquinone	10413	0.673	-18.799	1	C11 H12 O2	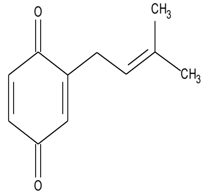	Prenyl-1,4-benzoquinone
1,2-Methylenedioxy-6a,7-dehydroaporphine-10,11-quinone	15583	0.668	-52.716	4	C18 H13 N1 O4	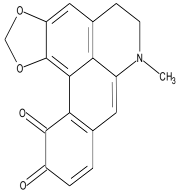	
2-Methoxybenzoquinone	9919	0.663	-19.424	1	C7 H6 O3	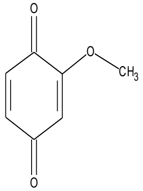	[Pl] Diospyros kaki
1-Hydroxy-5',8'-dimethoxy-3,3'-dimethyl-2,2'-binaphthyl-5,8,1',4'-diquinone	20175	0.662	-48.617	2	C24 H18 O7	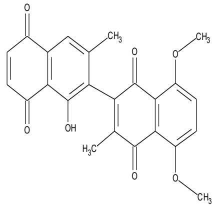	[synthetic]

(+)-Sclerotiorin	2887	0.662	-43.975	2	C21 H23 Cl1 O5	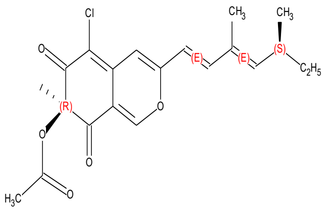	[F] Penicillium sclerotiorum, P. multicolor, P. implicatum	
Julichrome-Q1,4	4429	0.650	-95.213	3	C38 H34 O15	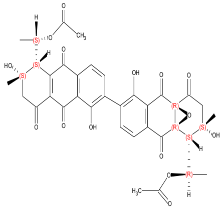	[B] Streptomyces shinodaensis	
Julichrome Q8,8	4433	0.649	-91.240	3	C38 H38 O14	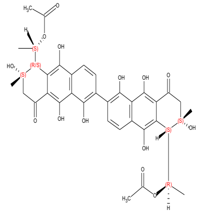	[B] Streptomyces shinodaensis	2
Julichrome Q3,8	1919	0.648	-82.146	3	C38 H38 O15	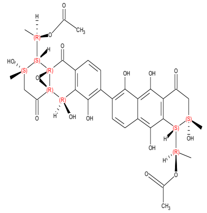	[B] Streptomyces shinodaensis	2
Variecolorquinone B	34846	0.643	-33.250	1	C17 H16 O6	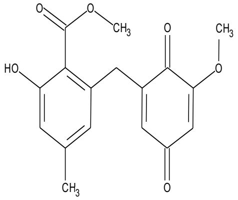	[F] Aspergillus variecolor B-17	

Julichrome Q1,7	4430	0.642	-62.501	3	C38 H36 O15	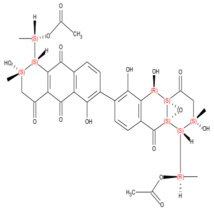	[B] Streptomyces shinodaensis	
2-Methyl-6-phytyl-p-benzoquinone	28681	0.642	-22.879	1	C27 H44 O2	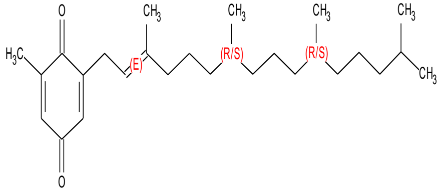	[A] brown alga Taonia atomaria, microalga Scenedesmus obliquus	
2-(2',3'-Dihydrosorbyl)-3,6-dimethyl-5-hydroxy-1,4-benzoquinone	32773	0.640	-25.339	1	C14 H16 O4	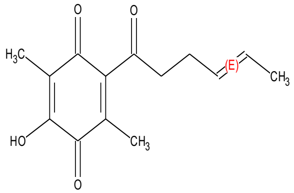	[F] marine Penicillium terrestre	
8',9'-Dihydroxysargaquinone	14878	0.639	-40.775	1	C27 H38 O4	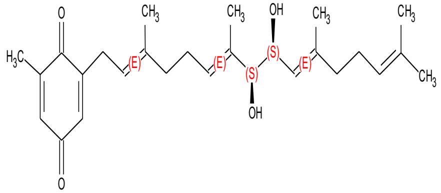		
2-Geranylgeranyl-6-methylbenzohydroquinone derivative	33984	0.633	-37.493	1	C22 H34 O4	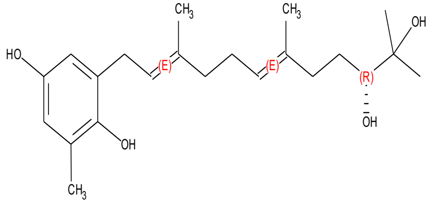	[A] brown alga Sargassum micracanthum.	2

Fallahydroquinone	36927	0.632	-42.319	1	C27 H40 O4	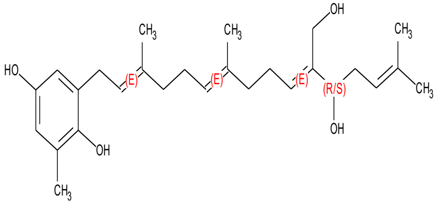	[B] brown alga Sargassum fallax	2
Menzohydroquinone	36652	0.632	-37.335	1	C27 H40 O3	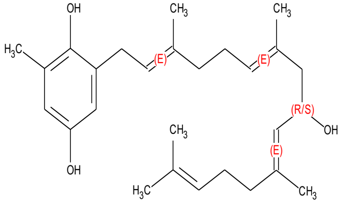	[A] brown alga Desmarestia menziesii	2
2-(Geranylgeranyl)-6-methyl-1,4-benzohydroquinone	18429	0.632	-26.479	1	C27 H40 O2	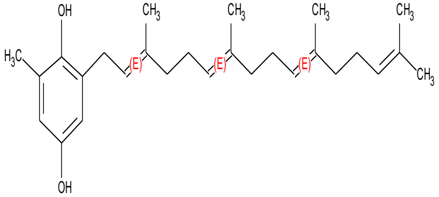	[A] brown alga Stypopodium flabelliforme [A] marine brown alga Halidrys siliquosa	2
2,3-Dimethoxy-1,4-benzoquinone	14207	0.632	-24.449	1	C8 H8 O4	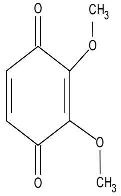	[An] millipede Floridobolus penneri Uroblaniulus canadensis Metiche tanganyicense Pachybolus brachysternus Phistreptus levis Spirostreptus pavani	
2,3-Dimethoxy-6-propyl-hydroquinone	9879	0.630	-25.323	1	C11 H16 O4	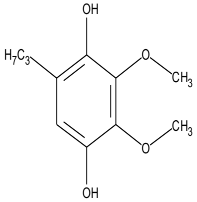	[F] Camarops microspora	2

11'-Methoxysargaquinone	14877	0.630	-29.582	1	C28 H40 O3	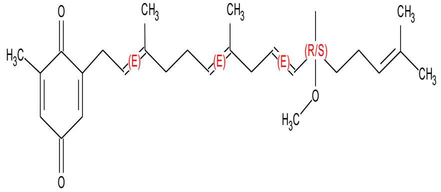	
2-Geranylgeranyl-6-methylbenzoquinone derivative	33985	0.629	-36.393	1	C22 H32 O4	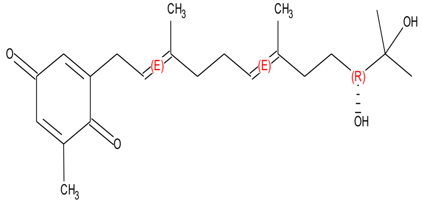	[A] brown alga Sargassum micracanthum.
6-Geranyl-2-methyl-1,4-benzoquinone	14842	0.629	-22.039	1	C17 H22 O2	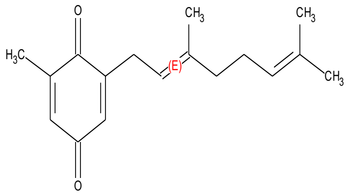	
n-Propyl-1,4-benzoquinone	14815	0.623	-17.452	1	C9 H10 O2	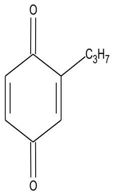	[An] Pedinini beetles
Toluquinone	10828	0.623	-17.728	1	C7 H6 O2	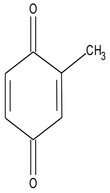	[F] Penicillium patulum; millipede Floridobolus penneri

Ethylbenzoquinone	10387	0.622	-17.591	1	C8 H8 O2	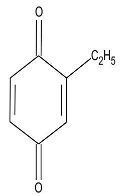		
(-)-Curcuhydroquinone	9805	0.621	-18.970	1	C14 H22 O2	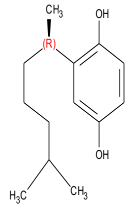	[An] Pseudopterogorgia rigida, Coelenterates	2
Capillaquinone	14856	0.621	-28.983	1	C22 H26 O3	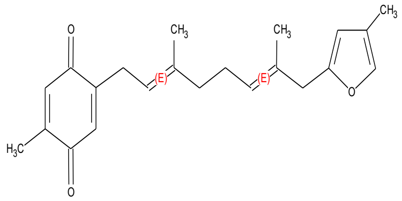		
5-Geranyl-2-methyl-1,4-benzoquinone	10021	0.620	-21.960	1	C17 H22 O2	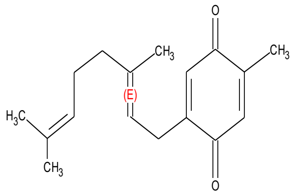		
5-Farnesyl-2-methyl-1,4-benzoquinone	14855	0.620	-24.016	1	C22 H30 O2	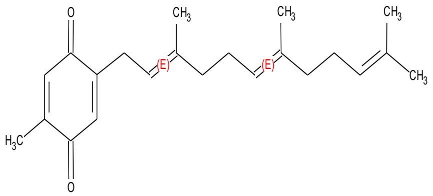	[F] Phellinus pini	

2-Hexaprenyl-1,4-benzoquinone	14891	0.619	-31.310	1	C36 H52 O2	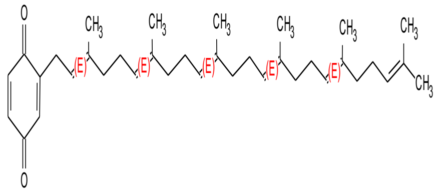	
2-Pentaprenyl-1,4-benzoquinone	14889	0.618	-29.902	1	C31 H44 O2	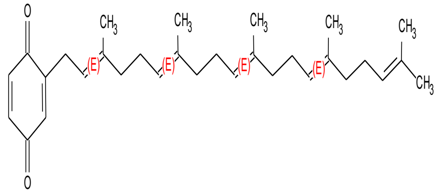	
Geranylgeranylbenzoquinone	14873	0.618	-25.940	1	C26 H36 O2	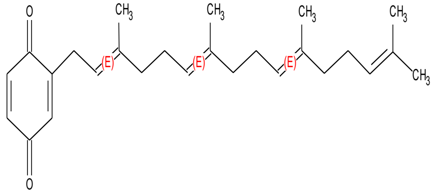	
7-Chlororubrocashmeriquinone	38593	0.615	-55.769	3	C14 H7 Cl1 O6	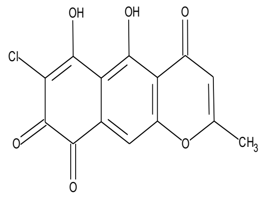	[L] Lethariella sernanderi, L. cashmeriana, L. sinensis
d-Tocopherylquinone	38928	0.615	-36.944	1	C27 H46 O3	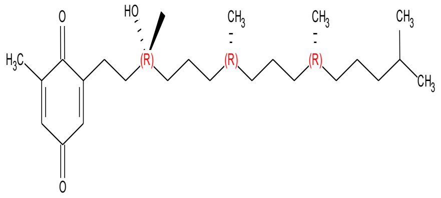	[A] red macroalga Callophycus serratus

(-)-Curcuquinone	9807	0.615	-17.896	1	C14 H20 O2	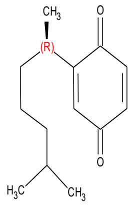	[An] Pseudopterogorgia ridiga (or rigida?), Coelenterates	
11-O-Methyl-epi-cochlioquinone A	31547	0.614	-43.427	4	C31 H46 O8	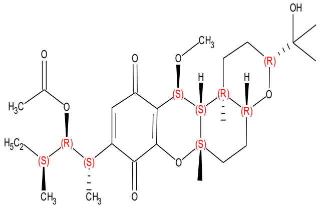	[F] Bipolaris brizae, Stachybotrys chartarum	
ortho-Naphthoquinone	20473	0.613	-29.503	2	C10 H6 O2	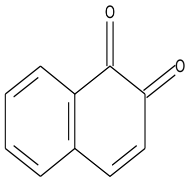	[synthetic]	
14-Epidihydrocochlioquinone-B	10900	0.611	-46.149	4	C28 H42 O6	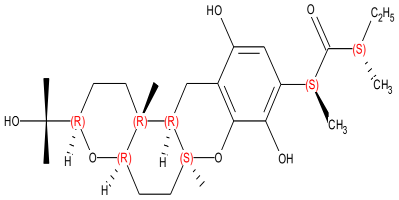	[F] (ascomycete) Neobulgaria pura	2
2-Acetoxycurcuquinone	14837	0.609	-27.626	1	C17 H22 O4	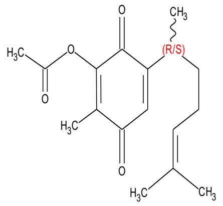	aerial parts of Coreopsis senaria80 and C. !asciculata81 - Thomson	

Hydroxy-2-octaprenyl-1,4-benzohydroquinone	14894	0.607	-47.249	1	C46 H70 O3	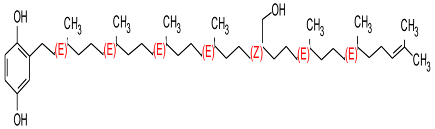		2
Geranylhydroquinone	10417	0.607	-22.577	1	C16 H22 O2	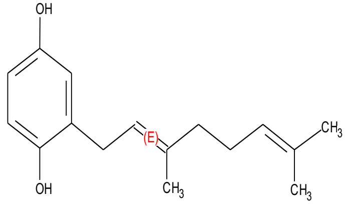	[An] Aplydium sp., Tunicates, Echinoderms	2
Farnesylhydroquinone	22152	0.607	-25.100	1	C21 H30 O2	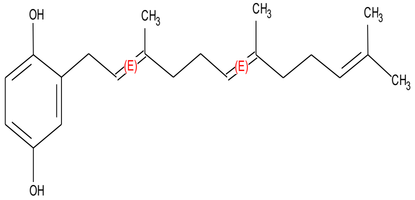	[A] alga Phaeophyta sp.	2
Asterriquinone A-1	3168	0.601	-46.732	1	C34 H34 N2 O4	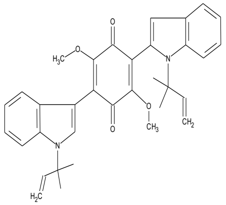	[F] Aspergillus terreus-africanus	
2,5-Dimethoxy-3,6-dimethylhydroquinone	9885	0.600	-16.221	1	C10 H14 O4	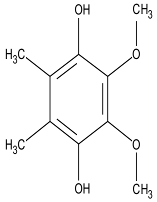	[F] Nectria coryoli	2

Rubrosinensiquinone A	40879	0.597	-71.171	3	C20 H17 N1 O8	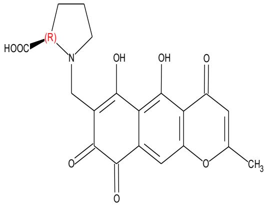	[L] Lethariella sinensis	
Desoxytridentoquinone	26167	0.597	-30.320	1	C26 H34 O3	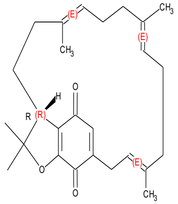	[F] Suillus tridentinus	
Ubiquinone precursor	17095	0.597	-39.541	1	C52 H78 O2	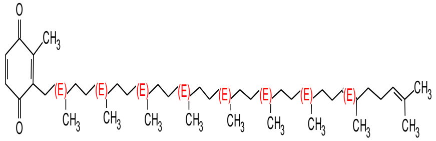	[B] Rhodospirillum rubrum	
7-Methoxy-malbranicin hydroquinone	30953	0.591	-35.647	1	C12 H16 O5	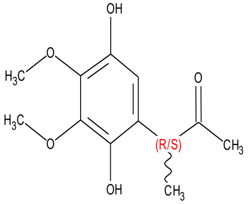	[F] Malbranchea cinnamomea HKI 286, HKI 296	2
Ubiquinone Q11	15208	0.590	-50.287	1	C64 H98 O4	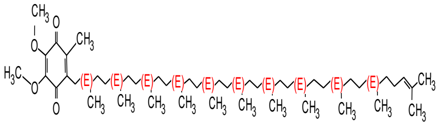		

Ubiquinone Q6	15755	0.590	-37.666	1	C39 H58 O4	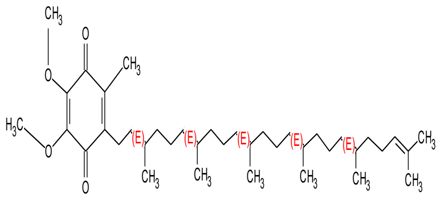	
Ubiquinone Q5	13454	0.590	-34.925	1	C34 H50 O4	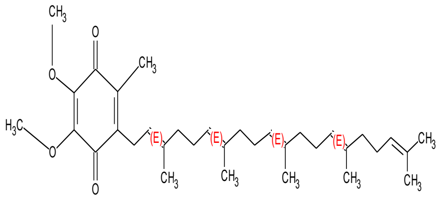	
Ubiquinone Q12	14826	0.589	-40.357	1	C69 H106 O4	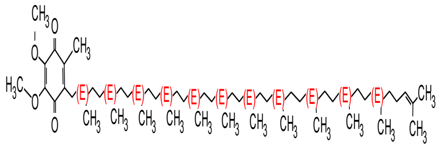	
Asterriquinone B-4	3171	0.585	-49.048	1	C34 H34 N2 O4	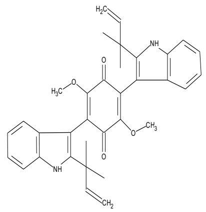	[F] Aspergillus terreus var. africanus (ifo 8835)
X-Dihydro-ubiquinone-10	17182	0.585	-41.838	1	C59 H92 O4	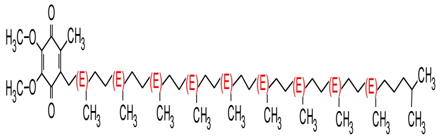	

2-[(2'E,6'E,10'E,14'Z)-5'-hydroxy-15'-hydroxymethyl-3',7',11'-trimethylhexadeca-2',6',10',14'-tetraenyl]-6-methylhydroquinone	17904	0.585	-37.511	1	C27 H40 O4	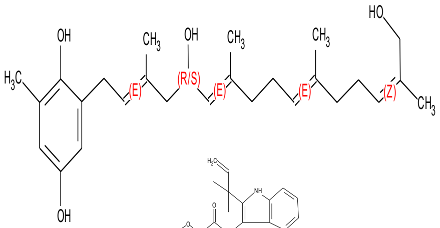	[A] Marine brown algae (Cystoseiraceae), Cystoseira squarrosa; [A] Acrocarpia, Acystis, Bifurcaria, Bifurcariopsis, Carpoglossum, [A] Caulocystis, Coccophora, Cystophora,	2
Asterriquinone A-4	3170	0.584	-54.315	1	C39 H42 N2 O4	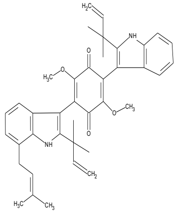	[F] Aspergillus terreus var. africanus (ifo 8835)	
Hibiscoquinone A	15169	0.583	-49.082	2	C15 H14 O4	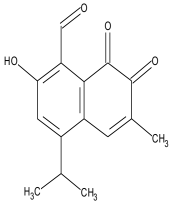		
(-)-Pleurotin	2657	0.583	-46.498	4	C21 H22 O5	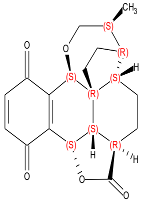	[F] Pleurotus griseus, Geopetalum geogenium, Hohenbuehelia geogenius, basidiomycete Nematoctonus robustus, Hohenbuehelia sp.	
Cochlioquinone C	20046	0.582	-34.003	4	C28 H40 O7	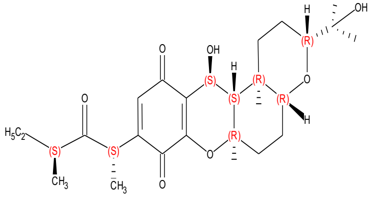	[F] plant pathogenic fungus Bipolaris cynodontis	

2,3-Didehydro-19a-hydroxy-14-epicochlioquinone B	41482	0.581	-58.418	4	C28 H38 O7	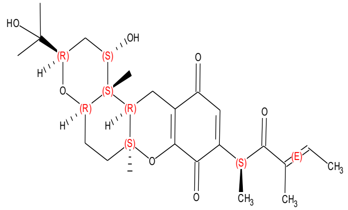	[F] marine-derived Nigrospora sp. MA75
8',9'-Diydroxy-5-methylsargaquinone	14879	0.579	-39.753	1	C28 H40 O4	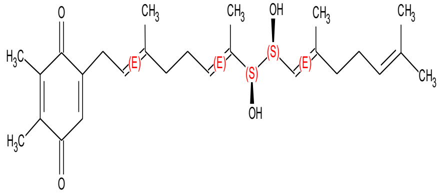	
2-Hydroxy-3-methyl-1,4-benzoquinone	14206	0.578	-31.281	1	C7 H6 O3	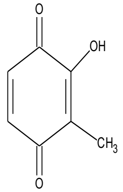	[An] millipede Floridobolus penneri
Ubiquinone precursor	16889	0.578	-43.813	1	C57 H86 O3	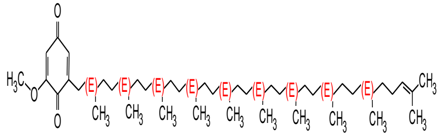	
Anhydrocochlioquinone A	34843	0.577	-46.814	4	C30 H42 O7	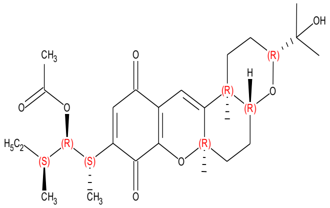	[F] Bipolaris oryzae

Stemphone	3521	0.577	-36.448	4	C30 H42 O8	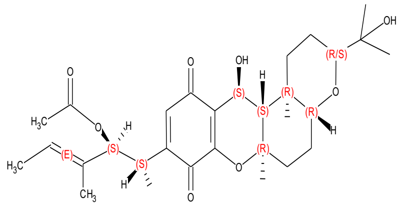	[F] Stemphylium sarcinae
(2'E, 6'E, 10'E, 14'E)-2-(8'-one-15'-formyl-3',7',11'-trimethylhexadeca-2',6',10',14'-tetraenyl)-6-methyl-1,4- benzoquinone	19709	0.575	-46.672	3	C27 H34 O4	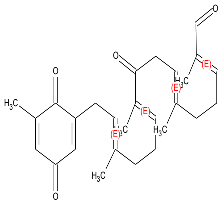	[A] brown alga Desmarestia menziesii
(3S)-Mucroquinone	9820	0.575	-43.970	1	C17 H16 O6	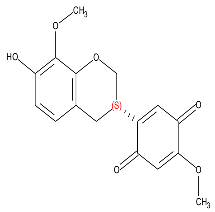	
Isospongiaquinone	10473	0.574	-31.365	1	C22 H30 O4	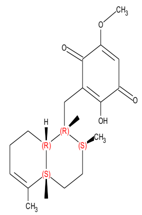	[An] Stelospongia conulata, Sponge
Plastoquinone-8	10694	0.573	-37.727	1	C48 H72 O2	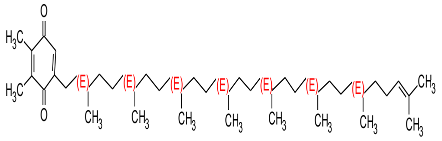	

Plastoquinone-3	10692	0.573	-22.693	1	C23 H32 O2	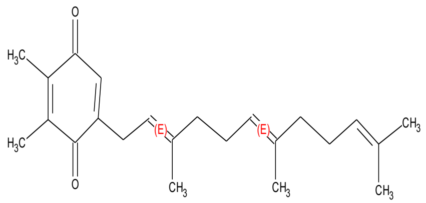	
Ubiquinone precursor	17033	0.573	-41.730	1	C52 H78 O3	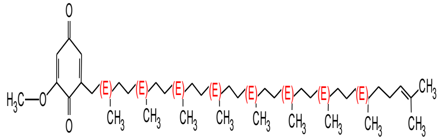	[B] Pseudomonas ovalis Ps. fluorescens Esch. coli
(2'E,6'E,10'E,14'E)-2-(8',9'-Dione-3',7',11',15'-tetramethylhexadeca-2',6',10',14'-tetraenyl)- 6-methyl-1,4-benzoquinone	19708	0.572	-44.926	3	C27 H34 O4	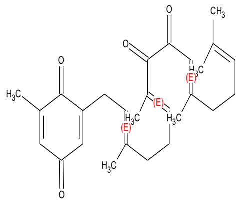	[A] brown alga Desmarestia menziesii
5,8-Dimethoxy-6-methyl-1,2-naphthoquinone	20181	0.572	-42.326	2	C13 H12 O4	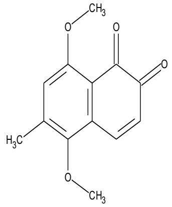	[synthetic]
(3R)-Mucroquinone	14901	0.571	-42.579	1	C17 H16 O6	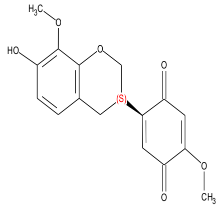	roots of Astragalus alexandrinus243 and A. trigonus243 (Fabaceae) - Thomson

Przewaquinone B	2711	0.570	-33.893	3	C18 H12 O4	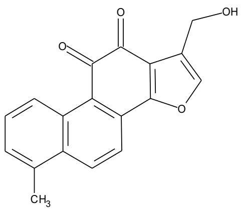	[Pl] Salvia miltiorrhiza	
d-tocopheryl-hydroquinone	38930	0.570	-20.566	1	C27 H48 O3	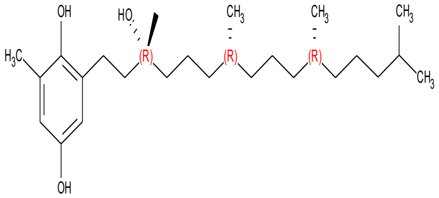	[A] red macroalga Callophycus serratus	2
Asterriquinone SU-5500	22933	0.570	-59.582	1	C34 H32 N2 O5	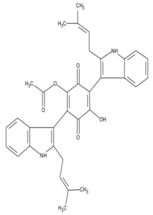	[F] Aspergillus candidus	
2,5-Dimethylbenzoquinone	9886	0.569	-16.888	1	C8 H8 O2	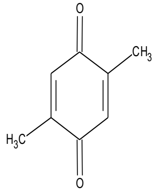		
2-Methoxy-6-(1-propyl)-1,4-benzoquinone	17672	0.569	-16.974	1	C11 H14 O2	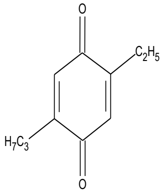		

Fumigatin	5410	0.569	-25.709	1	C8 H8 O4	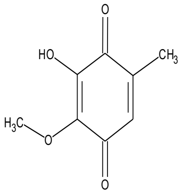	[F] Aspergillus fumigatus, Penicillium monoverticillata-stricta, P.spinulosum
Cochlioquinone-B	16523	0.568	-46.333	4	C28 H40 O6	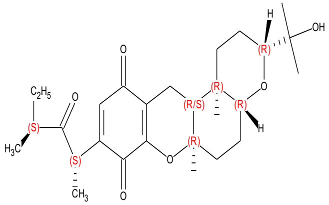	[F] Cochliobolus miyabeanus
(3R)-Claussequinone	14900	0.568	-40.271	1	C16 H14 O5	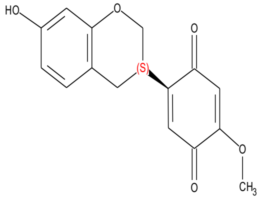	
Menzoquinone	36651	0.568	-48.239	1	C27 H36 O4	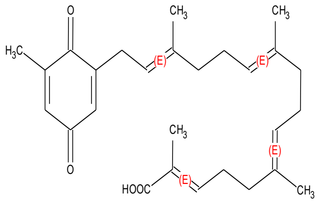	[A] brown alga Desmarestia menziesii
Ubiquinone Q10	15128	0.567	-49.203	1	C59 H90 O4	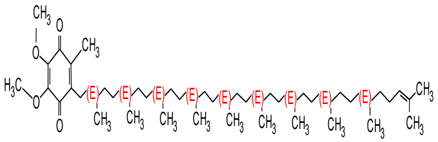	

(2'E,6'E,10'E,14'E)-2-(9'-Hydroxy-3',7',11',15'-tetramethylhexadeca-2',6',10',14'-tetraenyl)- 6-methyl-1,4-benzoquinone	20780	0.567	-34.925	1	C27 H38 O3	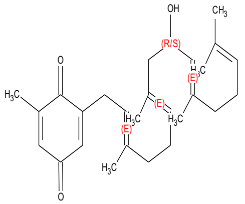	[A] brown alga Desmarestia menziesii
Sargaquinone	28678	0.566	-26.497	1	C27 H38 O2	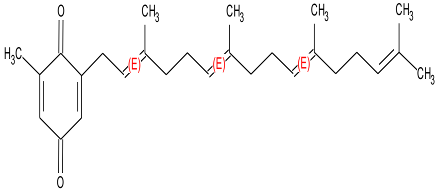	[A] brown alga Taonia atomaria; Dimocarpus fumatus
2,3-Dimethoxy-5-methyl-heptaprenyl-1,4-benzoquinone	9878	0.566	-42.326	1	C44 H66 O4	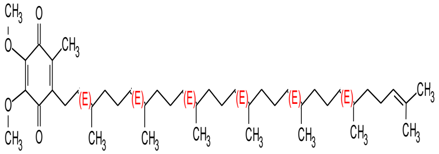	[B] Actinobacillus, Pasteurellaceae
9'-Methoxysargaquinone	14876	0.566	-31.478	1	C28 H40 O3	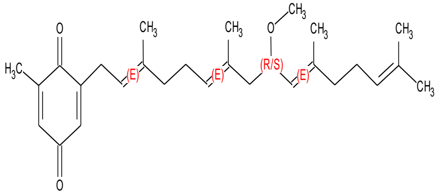	
10',11'-Dihydroxysargaquinone	14880	0.566	-41.029	1	C27 H40 O4	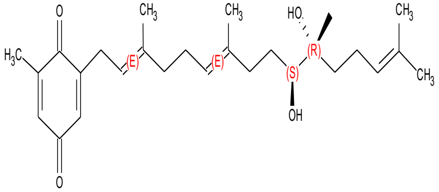	[A] brown alga Cystoseira crinita

Ubiquinone Q4	13303	0.566	-32.179	1	C29 H42 O4	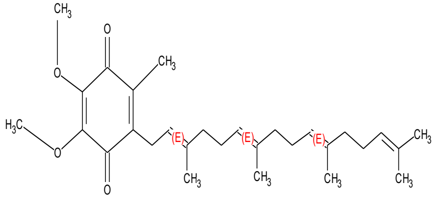	
Hibiscoquinone C	15171	0.565	-38.296	2	C14 H14 O3	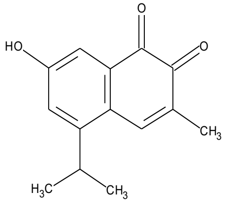	
Sargaquinone	14875	0.565	-26.951	1	C27 H38 O2	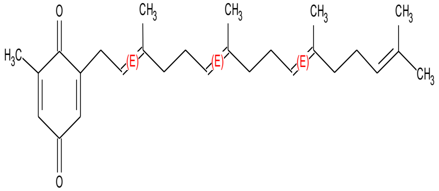	[A] marine brown algae Stypopodium flabelliforme, [A] Taonia atomaria
Fallaquinone	36928	0.565	-43.177	1	C27 H38 O4	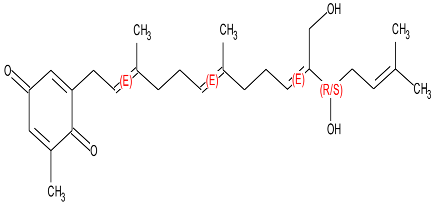	[B] brown alga Sargassum fallax
2-Hydroxy-3-methoxy-5-methyl-1,4-benzoquinone	3002	0.565	-26.838	1	C8 H8 O4	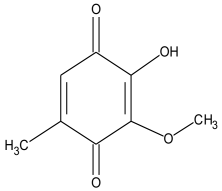	[F] Aspergillus fumigatus

Ubiquinone precursor	16043	0.565	-41.717	1	C57 H86 O2	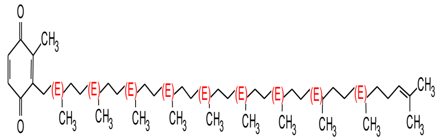	[B] Rhodospirillum rubrum
Cochlioquinone E	20047	0.565	-50.610	4	C28 H40 O7	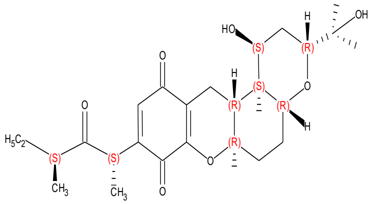	[F] plant pathogenic fungus Bipolaris cynodontis
(R)-Curcuquinone	14832	0.564	-18.836	1	C15 H20 O2	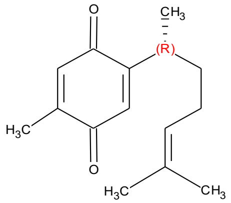	thomson
Rubrosinensiquinone C	40881	0.564	-48.552	3	C14 H8 O6	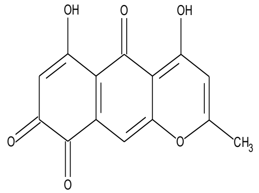	[L] Lethariella sinensis
Ubiquinone Q1	14829	0.564	-23.346	1	C14 H18 O4	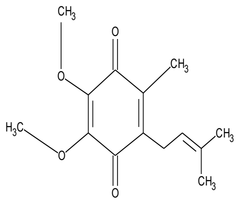	

Viomellein	5847	0.564	-72.434	3	C30 H24 O11	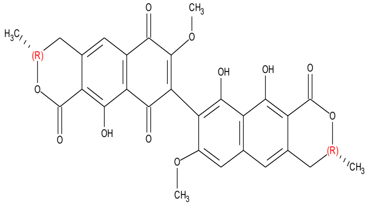	[F] Aspergillus sulphureus, A.melleus, A.ochraceus, Penicillium viridicatum, P.cyclopium, P.citreo-viride, Asp.ostianus Penic. citreo-viride biourge (Tue 553)
Tetrahydromethionaquinone	15136	0.561	-36.871	2	C46 H68 O2 S1	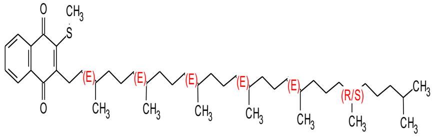	bacterium Hydrogenobacter thermophilus have now been published.35 It has also been isolated from H. halophilus. -thomson
Brasiliquinone-C	20596	0.561	-36.374	4	C21 H18 O5	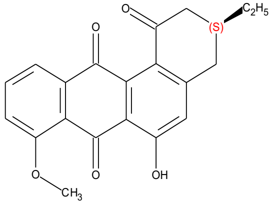	[B] Nocardia brasiliensis
2,3-Dimethoxy-6-propyl-1,4-benzoquinone	8916	0.560	-24.662	1	C11 H14 O4	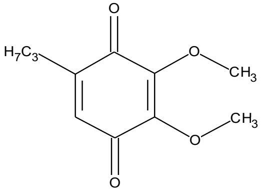	[F] Camarops microspora
5-(g,g-Dimethylallyl)-2-methylbenzoquinone	10014	0.559	-18.977	1	C12 H14 O2	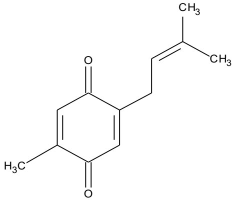	

5-Geranylgeranyl-2-methyl-1,4-benzoquinone	14874	0.559	-26.468	1	C27 H38 O2	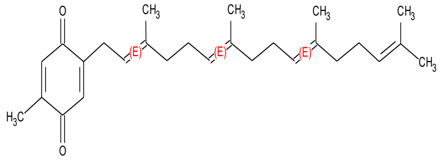	
Rubrocashmeriquinone	38592	0.558	-60.776	3	C14 H10 O6	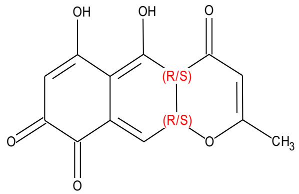	[L] Lethariella sernanderi, L. cashmeriana, L. sinensis
Sakyomicin C	4713	0.558	-53.692	4	C25 H26 O9	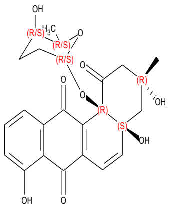	[B] Nocardia sp. 53, M53
Thymoquinone	10826	0.558	-17.061	1	C10 H12 O2	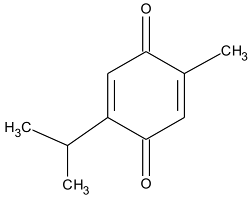	
3-Acetyl-5-hydroxy-2-methylnaphthoquinone	20160	0.556	-23.919	2	C13 H10 O4	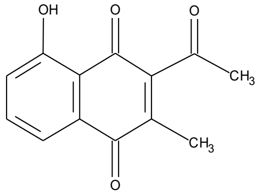	[synthetic]

2-Methoxy-5-methyl-1,4-benzoquinone	1975	0.555	-25.972	1	C8 H8 O3	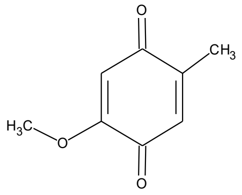	[F] Lenzites thermophila, Coprinus similis, Lentinus adhaerens	
5-Farnesyl-2-methyl-1,4-benzohydroquinone	30456	0.554	-23.743	1	C22 H32 O2	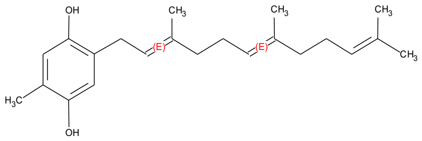	[F] marine fungus Penicillium sp.	2
Laurequinone	14833	0.554	-18.573	1	C15 H18 O2	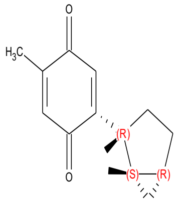		
Terrequinone A	31827	0.553	-53.666	1	C32 H30 N2 O3	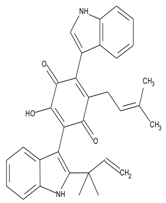	[F] Aspergillus terreus from the rhizosphere of sonoran desert plants	
Ubiquinone precursor	16676	0.552	-28.217	1	C28 H40 O3	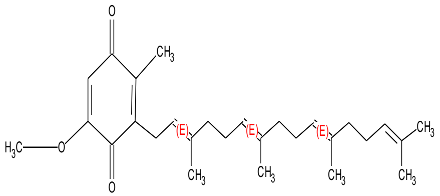		

Ubiquinone precursor	15833	0.552	-40.381	1	C58 H88 O3	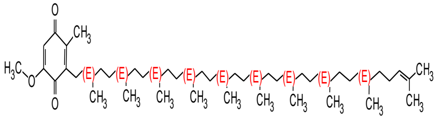	[B] Rhodospirillum rubrum
Ubiquinone precursor	14001	0.552	-29.144	1	C33 H48 O3	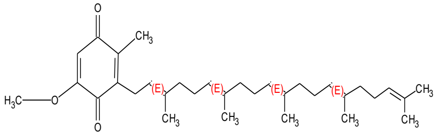	[B] Pseudomonas ovalis, Ps. fluorescens Esch coli
Ubiquinone precursor	16067	0.552	-34.515	1	C38 H56 O3	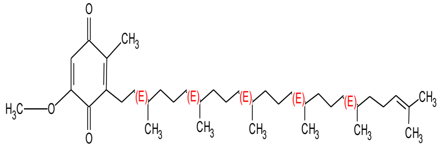	[B] Pseudomonas ovalis Ps. fluorescens Esch coli
8-Methoxy-3-methyl-1,2-naphthoquinone	10070	0.550	-36.991	2	C12 H10 O3	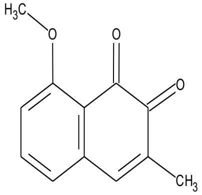	
3,4-Dehydroxanthomegnin	4026	0.550	-67.469	3	C30 H20 O12	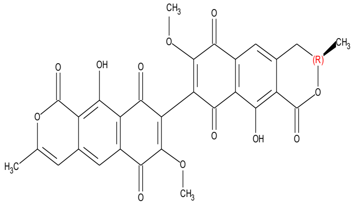	[F] Penicillium citreo-viride biourge (Tue 553), Nannizzia cajetani, Microsporum cookei

Cystoquinone	17988	0.549	-29.590	1	$C_{27} H_{34} O_{4}$	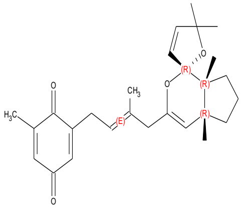	[A] alga Cystoseira amentacea (Bory) var. stricta (Montagne)
(S)-Lagopodin-A	2072	0.547	-27.739	1	$C_{15} H_{18} O_{3}$	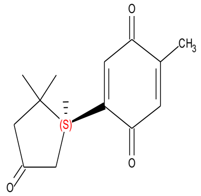	[F] Coprinus lagopus
Hymenoquinone	10443	0.547	-60.753	1	$C_{13} H_{8} O_{6}$	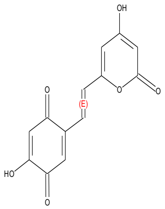	[F] Hymenochaete mougeotii
Cochlioquinone-A	14736	0.547	-51.624	4	$C_{30} H_{44} O_{8}$	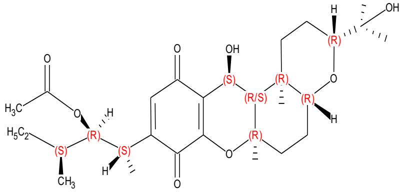	[F] Drechslera sacchari
Gonyleptidin	12358	0.546	-17.100	1	$C_{8} H_{8} O_{2}$	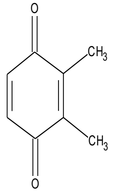	[x] Gonyleptideae

Ubiquinone precursor	15725	0.546	-44.747	1	C58 H88 O4	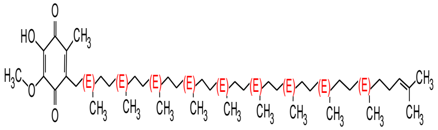	[B] Rhodospirillum rubrum
2,3-Dimethoxy-5-methyl-1,4-benzoquinone	14208	0.545	-25.481	1	C9 H10 O4	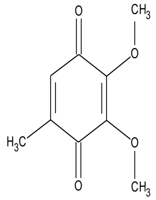	[An] millipede Floridobolus penneri; Rhapidostreptus innominatus; Phachybolus brachysternus; Aphistreptus levis; Metiche tanganyicense
Altersolanol-D	11433	0.545	-65.951	3	C16 H16 O8	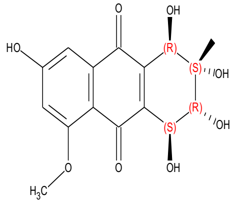	[F] Alternaria solani
Fumiquinone A	36774	0.544	-38.165	1	C12 H14 O6	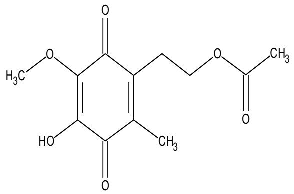	[F] Aspergillus fumigatus
3-Acetyl-5-hydroxy-7-methoxy-2-methylnaphthoquinone	21021	0.542	-28.584	2	C14 H12 O5	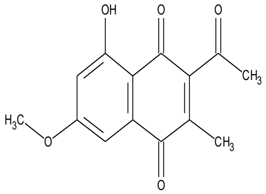	

Hyathellaquinone	25951	0.541	-35.122	1	C22 H30 O4	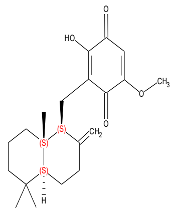	[F] discomycete Trichopezizella barbata SANK 25395
14-Epicochlioquinone-B	10899	0.540	-43.537	4	C28 H40 O6	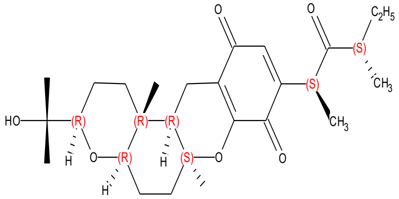	[F] (ascomycete) Neobulgaria pura
Rotundiquinone dimethyl ether	20218	0.537	-48.861	2	C24 H18 O6	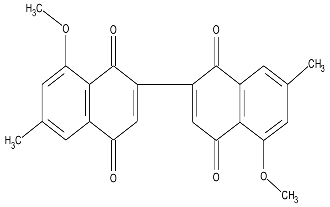	[synthetic]
Rubrogliocladin	3453	0.536	-24.404	1	C20 H26 O8	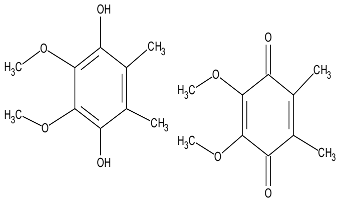	[F] Gliocladium roseum
Brasiliquinone-A	20594	0.535	-46.940	4	C26 H27 N1 O7	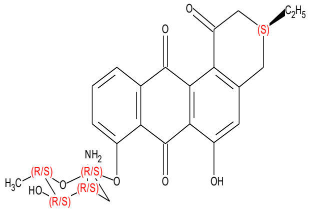	[B] Nocardia brasiliensis

Pycnanthuquinone C	34413	0.534	-35.710	2	C17 H22 O4	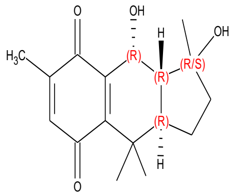	[A] brown alga Cystophora harveyi
Asterriquinone A-2	3169	0.534	-54.955	1	C39 H42 N2 O4	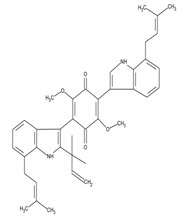	[F] Aspergillus terreus var. africanus (ifo 8835)
Asterriquinone C-1	3172	0.532	-48.951	1	C29 H26 N2 O4	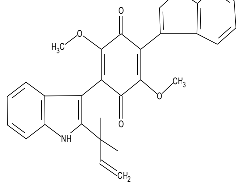	[F] Aspergillus terreus-africanus
5,8,8'-Trihydroxy-6,6'-dimethyl-2,2'-binaphthoquinone	20163	0.531	-33.323	2	C22 H14 O7	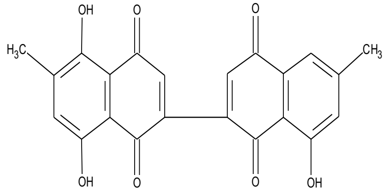	[synthetic]
3',4'-Dehydroviomellein	5982	0.530	-69.057	3	C30 H22 O11	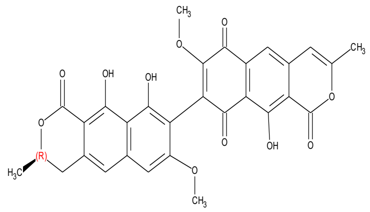	[F] Nannizzia cajetane uamh 3325

Diosquinone	10351	0.530	-44.204	2	C22 H14 O7	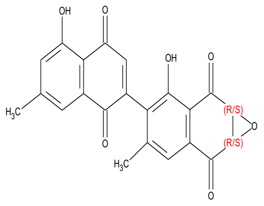	thomson
Deliquinone	22549	0.528	-36.500	1	C14 H18 O4	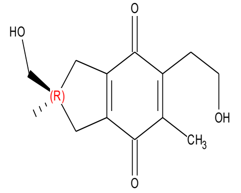	[F] Russula delica
3,6-Dihydroxythymoquinone	9941	0.528	-45.165	1	C10 H12 O4	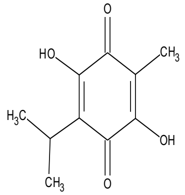	
Cochlioquinone D	20049	0.528	-43.744	4	C28 H38 O6	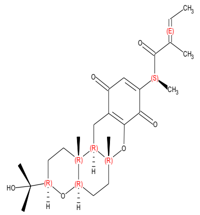	[F] plant pathogenic fungus Bipolaris cynodontis
Phytylplastoquinone	10685	0.528	-20.758	1	C28 H46 O2	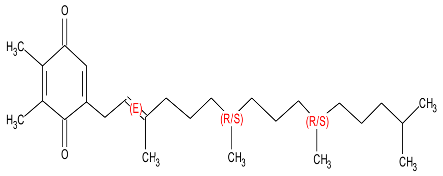	[A] Scenedesmus obliquus - Thomson

Rubrosinensiquinone B	40880	0.528	-70.606	3	C18 H15 N1 O8	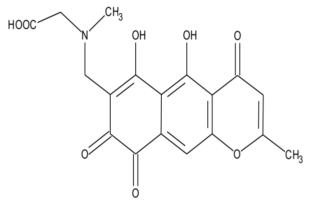	[L] Lethariella sinensis
Ventiloquinone-J	8775	0.527	-45.532	3	C17 H18 O6	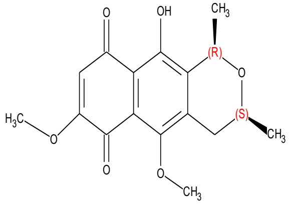	[Pl] Ventilago maderaspatana
Julichrome Q1,3	1913	0.526	-64.691	3	C38 H36 O15	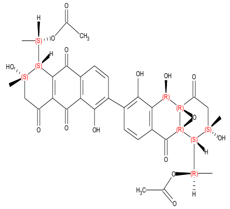	[B] Streptomyces shiodaensis
IFO 8835-10	6432	0.526	-52.721	1	C34 H34 N2 O4	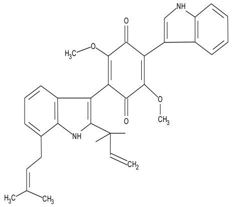	[F] Aspergillus terreus var. africanus (ifo 8835)
25-Dihydrosaframycin A	5977	0.523	-61.905	4	C29 H32 N4 O8	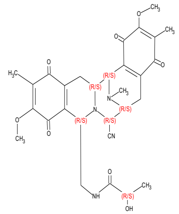	[B] Rhodococcus amidophilus ifm 144

Hinnuliquinone	1800	0.522	-65.452	1	$C_{32} H_{30} N_{2} O_{4}$	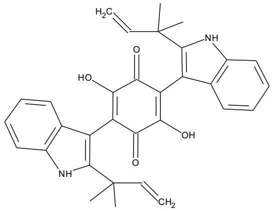	
Julichrome Q1,6	1914	0.522	-58.408	3	$C_{38} H_{36} O_{13}$	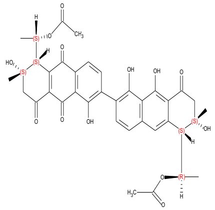	[B] Streptomyces shinodaensis
IFO 8835-8	6436	0.522	-50.051	1	$C_{34} H_{34} N_{2} O_{4}$	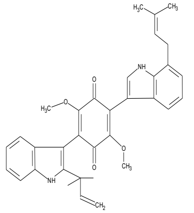	[F] Aspergillus terreus var. africanus (ifo 8835)
5-Hydroxy-6-methoxy-2,3-dimethyl-1,4-benzoquinone	10023	0.521	-24.893	1	$C_{9} H_{10} O_{4}$	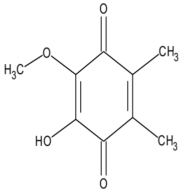	
Ardisiaquinone-C	10147	0.521	-65.245	1	$C_{31} H_{40} O_{9}$	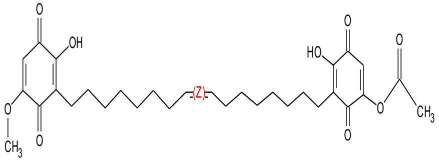	

IFO 8835-4	6435	0.520	-45.456	1	C34 H34 N2 O4	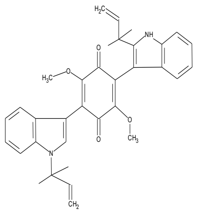	[F] Aspergillus terreus var. africanus (ifo 8835)
2,6-Di-tert.-butylbenzoquinone	23777	0.519	-16.176	1	C14 H20 O2	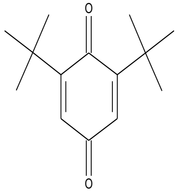	[D] odor compounds from lake of Galilee; alga Peridinium gatunense
Graphisquinone	14490	0.518	-31.391	1	C11 H10 O5	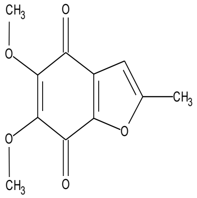	[L] lichen Graphis scripta, G. desquamescens
Methylenediboviquinone-3,4	17685	0.516	-82.513	1	C48 H64 O8	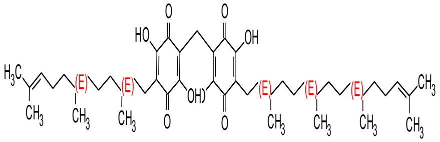	
Ilimaquinone	10452	0.516	-23.325	1	C22 H30 O3	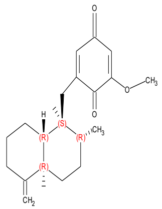	[An] Hippospongia metachriia, Sponge

2-Methoxy-6-propyl-hydroquinone	9918	0.515	-19.492	1	C10 H14 O3	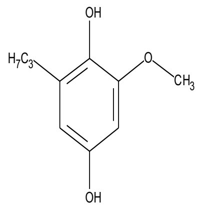	[F] Camarops microspora	2
2-Methoxy-6-propyl-1,4-benzoquinone	1976	0.515	-19.492	1	C10 H12 O3	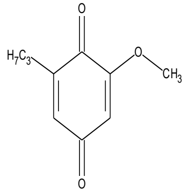	[F] Camarops microspora	
Asterriquinone-C-1 quinol	16237	0.515	-52.582	1	C29 H28 N2 O4	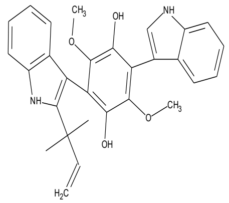	[F] Aspergillus terreus	2
Irisquinone	14819	0.514	-23.984	1	C24 H38 O3	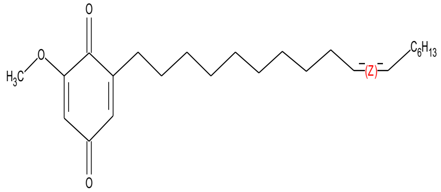	[Pl] Iris pseudacorus	
4,9-Dihydroxy-1,2,6,7,11,12-hexymethylperylene-3,10-quinone	15471	0.514	-62.433	4	C26 H22 O4	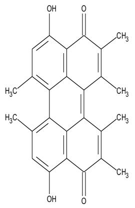		

Dietchequinone	14820	0.514	-27.182	1	C24 H38 O3	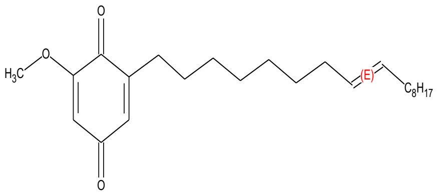	[Pl] Cyperus dietricheae	
Tetrahydrocyperaquinone	10817	0.514	-31.806	3	C14 H14 O4	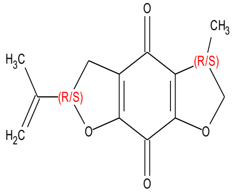		
3-Methoxy-2,5-toluquinone	6002	0.513	-19.361	1	C8 H8 O3	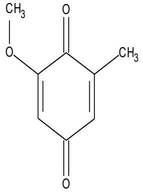	[F] Aspergillus sp. hpl y-30212	
Tocopheryl-hydroquinone	38929	0.513	-28.750	1	C28 H50 O3	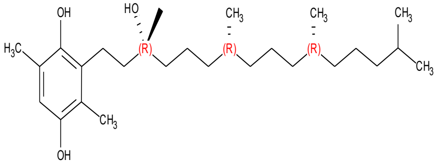	[A] red macroalga Callophycus serratus	2
Spinulosin	5747	0.512	-37.060	1	C8 H8 O5	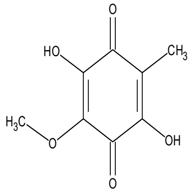	[F] Aspergillus fumigatus, Penicillium spinulosum, P.cinerascens P.monoverticillata-stricta	

Pisoquinone	13320	0.510	######	2	C35 H18 O16	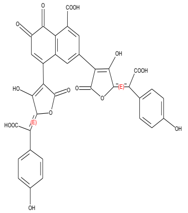	[F] gastromycete fungus Pisolithus arhizus
17-Methoxycochlioquinone A	31546	0.507	-47.817	4	C31 H46 O9	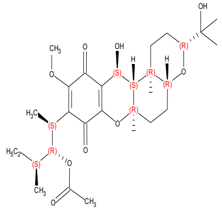	[F] Bipolaris brizae, Stachybotrys chartarum
Saframycin-C	4710	0.503	-57.384	4	C29 H33 N3 O9	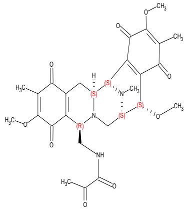	[B] Streptomyces lavendulae 314 FERM-p 3218, NRRL 11002
Saframycin-B	4709	0.503	-54.029	4	C28 H31 N3 O8	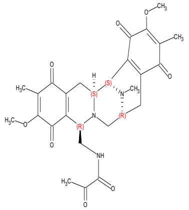	[B] Streptomyces lavendulae 314 FERM-p 3218, NRRL 11002
6-Hexanoyl-2,5,7,8-tetrahydroxy-1,4-naphthoquinone	15151	0.502	-56.820	2	C16 H16 O7	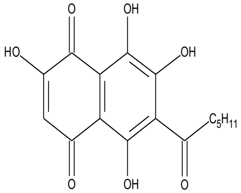	

2,3,5-Trimethylbenzoquinone	9871	0.498	-16.806	1	C9 H10 O2	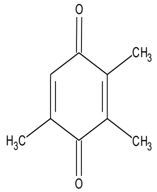	
Caldariellaquinone	15592	0.498	-21.165	1	C39 H66 O2 S2	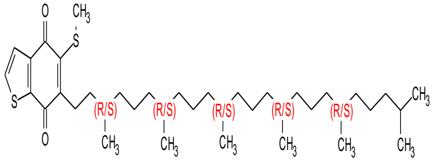	cultures of Sulfolobus ambivalens30 and S. acidocaldarius. THOMSON
Cyclospongiaquinone-1	14870	0.497	-35.534	4	C22 H30 O4	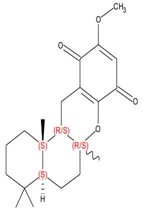	
Dihydrophytylplastoquinone	14895	0.497	-20.492	1	C28 H48 O2	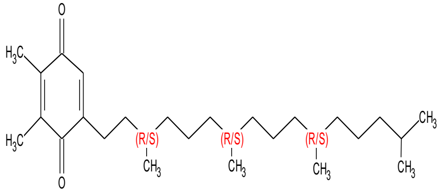	
Boviquinone-3	14857	0.496	-47.803	1	C21 H28 O4	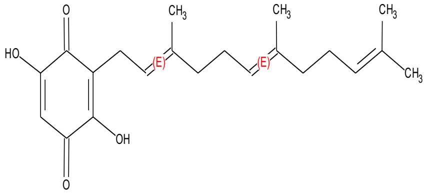	[F] Gomphidius rutilus, Chroogomphus helveticus, C. rutilus

5-Chloro-3,6-dihydroxy-2-methyl-1,4-benzoquinone	14825	0.496	-38.580	1	C7 H5 Cl1 O4	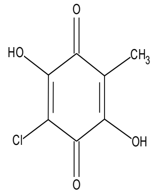	[F] Aspergillus terreus	
Asterriquinone-A-1 quinol	16234	0.496	-46.598	1	C34 H36 N2 O4	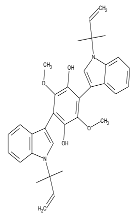	[F] Aspergillus terreus	2
2,3-Dimethoxy-5,6-dimethyl-1,4-benzoquinone	1030	0.495	-24.404	1	C10 H12 O4	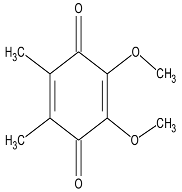	[F] Gliocladium roseum	
Asterriquinone monoacetate	11542	0.493	-53.314	2	C34 H32 N2 O5	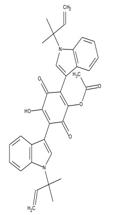	[F] Aspergillus terreus IFO 6123	
Fuscofusarin	10412	0.491	-64.455	3	C30 H20 O11	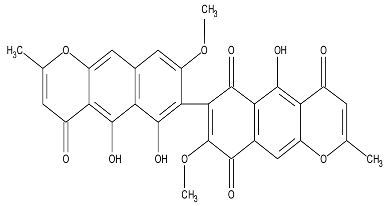	[F] Fusarium culmorum	

Plastoquinone-4	10693	0.490	-26.649	1	C28 H40 O2	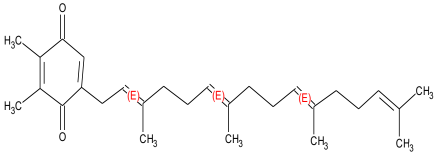	
IFO 8835-12	6433	0.490	-50.295	1	C29 H26 N2 O4	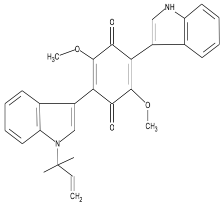	[F] Aspergillus terreus var. africanus (ifo 8835)
Plastoquinone-9	10695	0.490	-39.525	1	C53 H80 O2	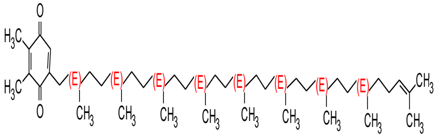	
2,5-Dihydroxy-1,4-benzoquinone	2997	0.489	-46.761	1	C6 H4 O4	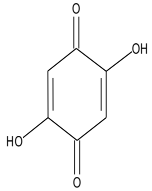	[F] Boletales
Hibiscoquinone D	15172	0.489	-58.613	2	C15 H12 O5	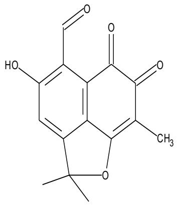	

3-Acetonyl-2,6-dimethyl-5-hydroxy-1,4-benzoquinone	32774	0.489	-38.141	1	C11 H12 O4	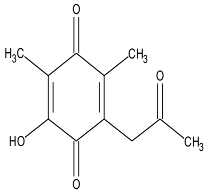	[F] marine Penicillium terrestre
Ubiquinone precursor	17032	0.488	-39.415	1	C53 H80 O3	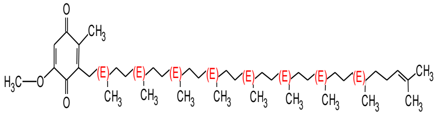	[B] Pseudomonas ovalis Ps. fluorescens Esch. coli
2,5-Dihydroxy-3,6-diphenyl-1,4-benzoquinone	1035	0.487	-41.665	1	C18 H12 O4	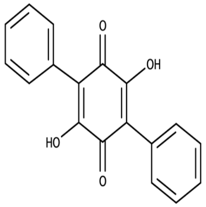	[F] Polyporus nidulans, Peniophora filamentosa, Lopharia papyracea, [F] Sticta coronata, Polyporus rutilans
Przewaquinone A	2710	0.487	-32.142	3	C19 H18 O4	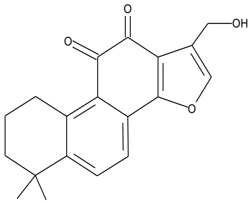	[Pl] Salvia miltiorrhiza
Ubiquinone precursor	16048	0.487	-35.188	1	C43 H64 O3	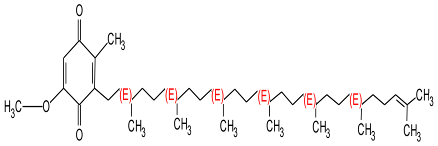	[B] Pseudomans ovalis, Pseudomonas fluorescens, Escherichia coli

Ubiquinone precursor	16470	0.486	-36.490	1	C48 H72 O3	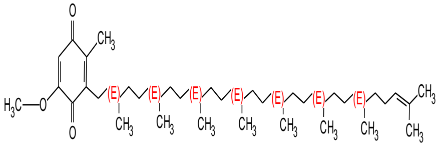	[B] Pseudomonas alkanolytica
Deflectin 2b	1372	0.484	-46.378	2	C26 H34 O5	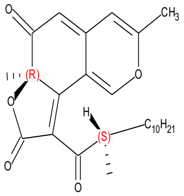	[F] Aspergillus deflectus
Ascocorynin	348	0.484	-51.411	1	C18 H12 O5	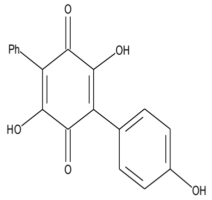	[F] Ascocoryne sarcoides wq 6979
49A	106	0.484	-50.311	4	C22 H26 N4 O5	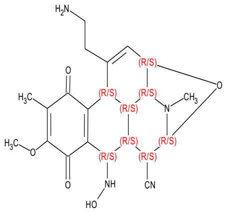	[B] Streptomyces flavogriseus 49 (ATCC 31386, FERM-p 4400)
Brasiliquinone-D	25073	0.483	-67.193	4	C28 H29 N1 O8	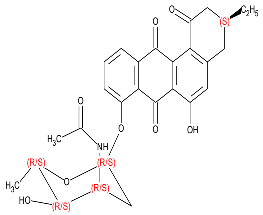	[B] Actinomycete Nocardia brasiliensis IFM 0667

2,5-Dimethoxy-1,4-benzoquinone	1038	0.483	-18.670	1	C8 H8 O4	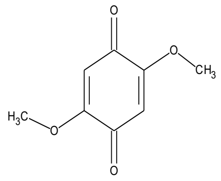	[F] Polyporus fumosus, Lenzites thermophila, Trichoderma pseudokoningii, [F] Gloeophyllum sepiarum
Didemethylasterriquinone D	17982	0.483	-58.508	1	C22 H14 N2 O4	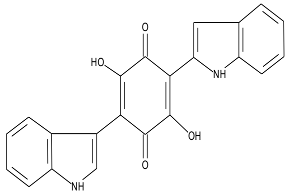	[F] fungus Chrysosporium merdarium P-5656
(3R)-Dihydroscabequinone	20459	0.483	-30.798	2	C15 H18 O4	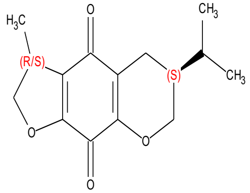	
3-Hydroxy-5-methoxy-2-methylbenzoquinone	9963	0.482	-28.280	1	C8 H8 O4	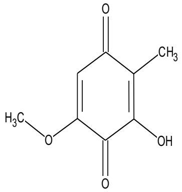	[F] Lenzites thermophila
2,5-Dihydroxy-3-(3,4-dihydroxyphenyl)-6-phenyl-1,4-benzoquinone	29610	0.481	-62.084	1	C18 H12 O6	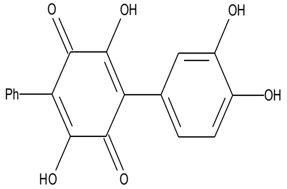	[F] Fusarium sp., Stilbella sp. strain 1586

Psoralenquinone	14923	0.481	-34.883	2	C11 H4 O5	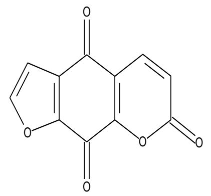	
L-783281	24833	0.480	-61.120	1	C32 H30 N2 O4	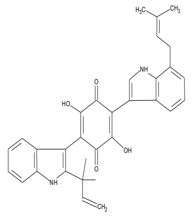	[F] fungus Pseudomassaria sp.
2-Hydroxy-5-methoxy-3-pentadecenyl-1,4-benzoquinone	9908	0.480	-32.095	1	C22 H34 O4	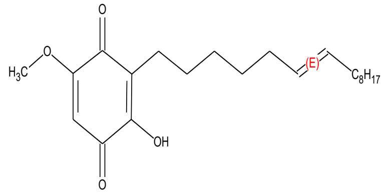	
5,6-Dimethoxy-2-methyl-1,4-naphthoquinone	10008	0.479	-23.375	2	C13 H12 O4	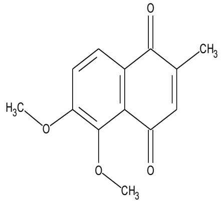	
2,5-Dimethyl-3-methoxy-1,4-benzoquinone	14205	0.478	-17.872	1	C9 H10 O3	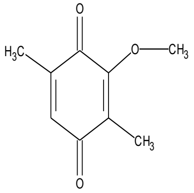	[An] millipede Floridobolus penneri

Yerrinquinone	8803	0.478	-39.081	2	C14 H12 O7	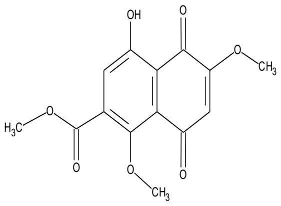	[F] fungus-infected wood of Diospyros montana- se thomson
6-(1-Acetoxyethyl)2,7-dimethoxyjuglone	8000	0.478	-49.746	2	C16 H16 O7	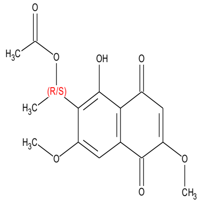	[F] (fungus) Kirschsteiniothelia sp.; Hendersonula toruloidea; [F] endophyte from (Pl) Schinus molle
Fumiquinone B	35583	0.476	-17.691	1	C8 H8 O5	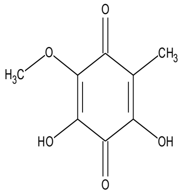	[F] Aspergillus fumigatus
iso-Asterriquinone	12531	0.476	-60.540	1	C32 H30 N2 O4	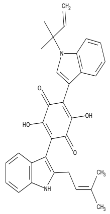	[F] Aspergillus terreus IFO 6123
Shanorellin	10774	0.475	-33.295	1	C9 H10 O4	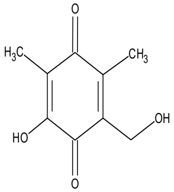	[F] Shanorella spirothrica

Oosporein	5597	0.475	-57.268	2	C14 H10 O8	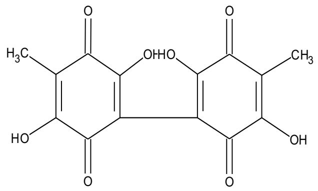	[F] Penicillium phoeniceum, P.rubrum, Oospora coloreus, Chaetomium aureum, C.trilaterale, Acemonium spp., Beauveria bossiana, Oospora colorans, Acremonium sp., Phelebia sp.,
Maesaquinone monoacetate	10531	0.472	-40.688	1	C28 H44 O5	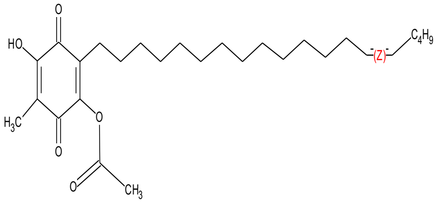	as a monoacetate in fresh fruit, bark and leaves of Maesa lanceolata 55; 70 (n = 9) in aerial parts of M. lanceolata244 (Myrsinaceae). - thomson
Mavioquinone	14822	0.472	-22.971	1	C28 H48 O3	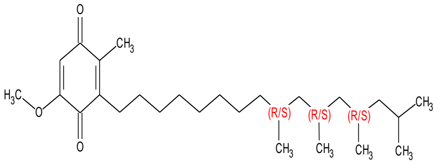	
(R)-Stenocarpoquinone A	15046	0.472	-37.947	3	C15 H14 O4	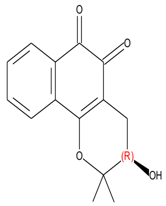	
Lagopodin-B	4451	0.471	-34.308	1	C15 H18 O4	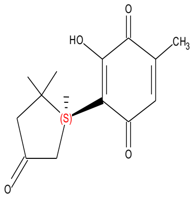	[F] Coprinus lagopus

4-Methoxy-1,2-naphthoquinone	20474	0.471	-37.464	2	C11 H8 O3	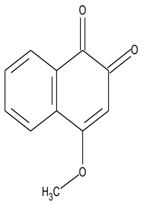	[synthetic]	
IFO 8835-16	6434	0.470	-53.380	1	C29 H26 N2 O4	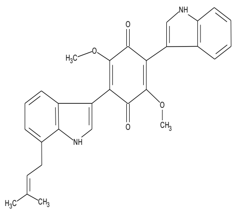	[F] Aspergillus terreus var. africanus (ifo 8835)	
Leucohymenoquinone	10506	0.470	-44.511	1	C13 H10 O6	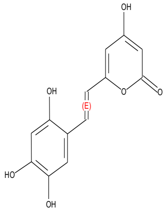	[F] Hymenochaete mougeotii	2
Saframycin AR3	4707	0.470	-60.760	4	C28 H33 N3 O8	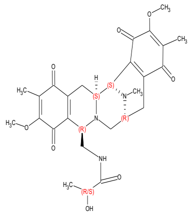	[B] Streptomyces lavendulae, Rhodococcus amidophilus ifm 144	
4'-Hydroxyphlebiarubrone	4050	0.468	-51.104	1	C19 H12 O5	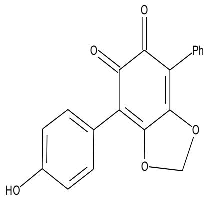	[F] Punctularia atropurpuascens (ha 193-82 & cbs 407.70).	

3-Acetyl-5,7-dimethoxy-2-methylnaphthoquinone	21022	0.468	-34.865	2	C15 H14 O5	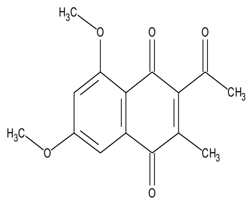	
Asterriquinone-D	382	0.467	-52.078	1	C24 H18 N2 O4	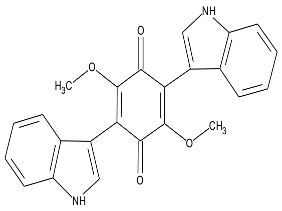	[F]Aspergillus terreus var. africanus (ifo 8835)
Mutaquinone-A	23233	0.466	-40.087	1	C23 H34 O4	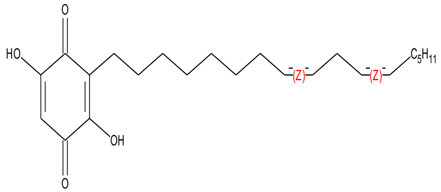	[F] wood-rotting basidiomycete Hapalopilus mutans
Aurofusarin	10164	0.466	-75.112	3	C30 H18 O12	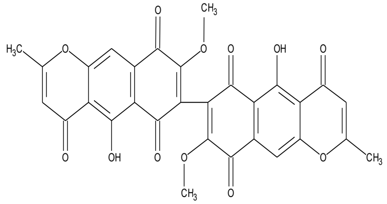	[F] Fusarium culmorum, Hypomyces vasellus
Altersolanol-F	11435	0.464	-62.643	3	C16 H16 O8	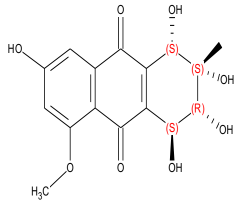	[F] Alternaria solani

Asterriquinone	10157	0.460	-56.588	1	C32 H30 N2 O4	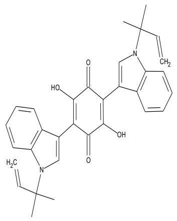	[F] Aspergillus terreus, A. candidus
Napyradiomycin B2	2330	0.459	-45.251	3	C25 H28 Cl2 O5	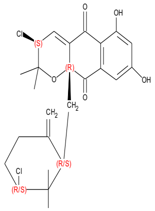	[B] Chainia rubra mg802-af1
Dimethoxy-p-xylohydroquinone	11890	0.459	-25.127	1	C10 H14 O4	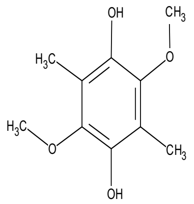	[F] Nectria coryoli
a-Tocopherolquinone	10083	0.457	-26.836	1	C29 H50 O3	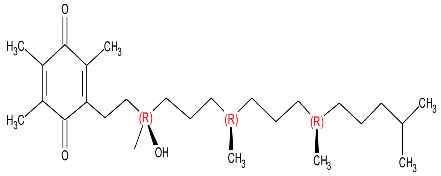	[A] Codium iyengarii [F] endophytic Annulohypoxylon boveri var. microspora BCRC 34012
Ecklonoquinone B	14831	0.456	-39.391	3	C25 H30 O6	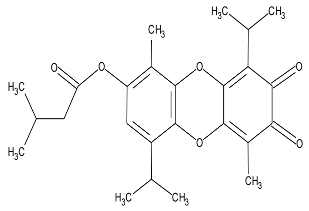	thomson

Ecklonoquinone A	14830	0.455	-39.407	3	C25 H30 O6	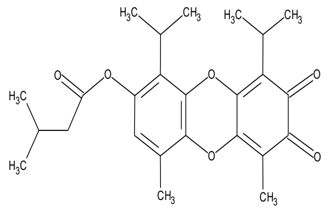	thomson
Furanonaphthoquinone	7978	0.455	-45.519	2	C22 H26 O5	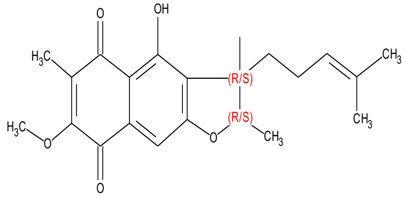	[B] Streptomyces cinnamonensis
Endocrocin 6,8-dimethyl ether	15339	0.455	-52.385	3	C18 H14 O7	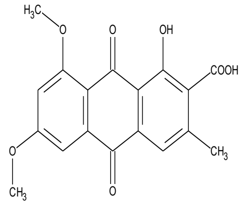	[F] Cortinarius armillatus, C. miniatopus
3-Hydroxytoluquinone	27905	0.453	-19.802	1	C7 H6 O3	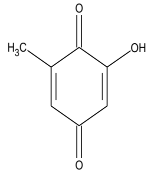	[F] Aspergillus fumigatus Fres.
Mavioquinone	14821	0.452	-21.761	1	C28 H48 O3	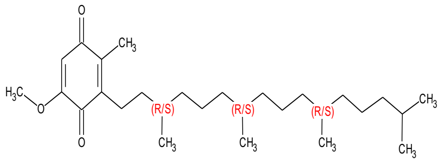	

Halenaquinone	15591	0.452	-42.298	4	C20 H12 O5	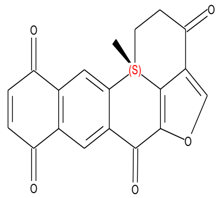	n an Adocia Sp.414 sponge, the quinol occurs in Xestospongia sapra; in the sponge Xestospongia cf. Carbonaria - THOMSON
Betulachrysoquinone	17523	0.452	-32.628	1	C20 H32 O4	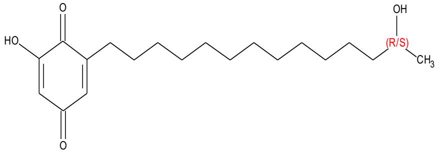	
Boviquinone-4	10192	0.450	-50.358	1	C26 H36 O4	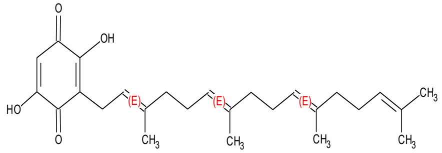	
3',4',4''-Trihydroxyphlebiarubrone	5981	0.448	-70.373	1	C19 H12 O7	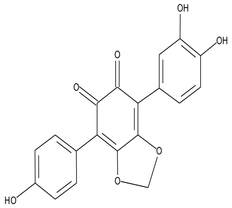	[F] Punctularia atropurpurascens (ha 193-82 & cbs 407.70), Penicillium strigosozonata (cbs 308.78)
6-Methyldihydrophytylplastoquinone	14896	0.447	-19.059	1	C29 H50 O2	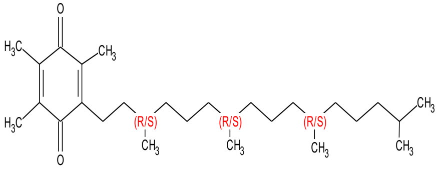	

5,5',8-Trihydroxy-7,7'dimethyl-2,2'-binaphthoquinone	20162	0.446	-33.909	2	C22 H14 O7	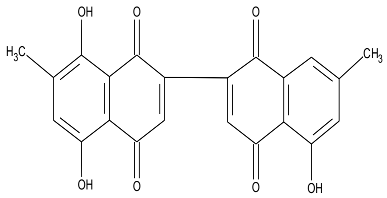	[Pl]; synthetic
Saframycin-D	4711	0.445	-59.784	4	C28 H31 N3 O9	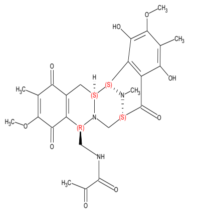	[B] Streptomyces lavendulae 314 FERM-p 3218, NRRL 11002
Salmoquinone	37150	0.445	-12.057	1	C17 H18 O7	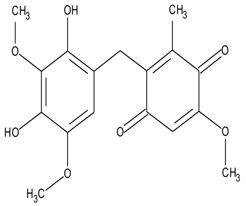	[F] Antrodia salmonea
3-Libocedroxythymoquinone	9967	0.445	-32.951	1	C32 H40 O6	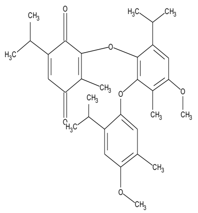	
Amorphaquinone	14902	0.444	-36.548	1	C18 H18 O7	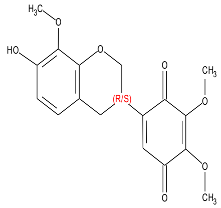	

3,6-Dihydroxy-2-methyl-1,4-benzoquinone	14824	0.443	-44.191	1	C7 H6 O4	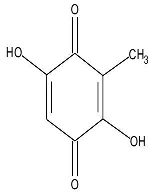	[F]
(3S)-Abruquinone B	14904	0.443	-38.664	1	C20 H22 O8	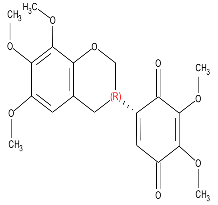	
Dehydrocyclospongiaquinone-1	14871	0.442	-33.898	4	C22 H28 O4	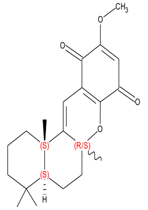	
Rubiflavin	2839	0.442	-72.232	4	C41 H50 N2 O10	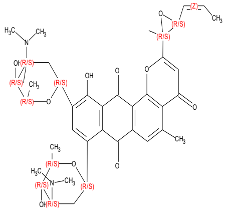	[B] Streptomyces sp. sc 3728, S.griseus
Xylariaquinone A	34863	0.442	-29.874	1	C15 H12 O5	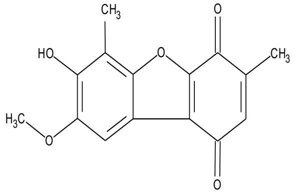	[F] endophytic Xylaria sp.

Spongiaquinone	10786	0.441	-30.385	1	C22 H30 O4	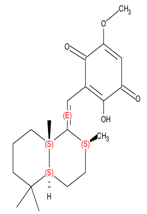	[An] Stelospongia conulata, Sponge
(3S)-Abruquinone A	14903	0.441	-36.763	1	C19 H20 O7	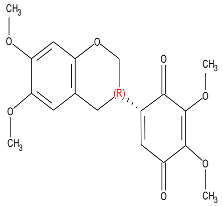	
Asterriquinone SU-5503	22934	0.441	-67.301	1	C33 H30 N2 O6	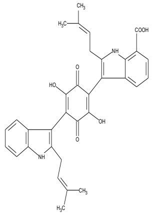	[F] Aspergillus candidus
Mutaquinone-D	23236	0.440	-45.876	1	C21 H34 O4	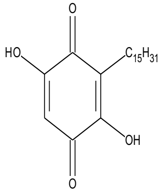	[F] wood-rotting basidiomycete Hapalopilus mutans
(3S)-Abruquinone C	14905	0.440	-45.889	1	C19 H20 O8	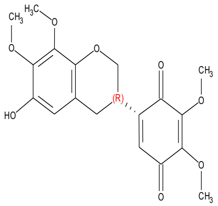	

2-Ethyl-3,6-dihydroxy-1,4-benzoquinone	9904	0.440	-42.269	1	C8 H8 O4	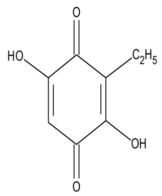	
Mutaquinone-B	23234	0.440	-45.614	1	C23 H36 O4	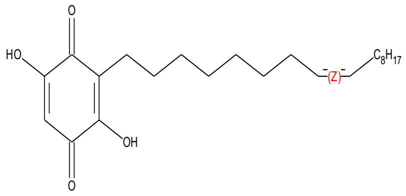	[F] wood-rotting basidiomycete Hapalopilus mutans
Mutaquinone-C	23235	0.440	-45.853	1	C23 H38 O4	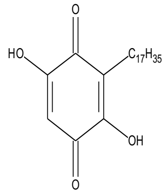	[F] wood-rotting basidiomycete Hapalopilus mutans
Ardisiaquinone-B	10146	0.440	-69.921	1	C29 H38 O8	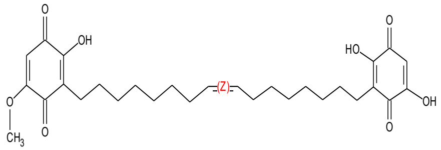	
Asterriquinone SU-5228	22932	0.438	-61.385	1	C27 H22 N2 O4	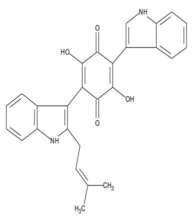	[F] Aspergillus candidus

Ardisiaquinone-A	10145	0.438	-58.342	1	C30 H40 O8	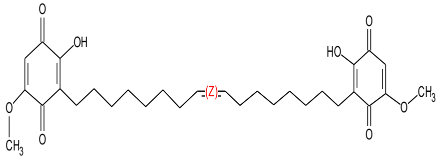	roots of seedlings of Sorghum bicolor69 (Gramineae). - thomson
Ubiquinone Q9	16491	0.437	-45.979	1	C54 H82 O4	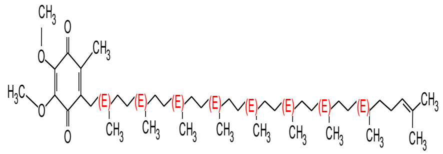	[B] Pseudomonas chlororaphis I-112
Breviquinone	14916	0.437	-29.335	1	C15 H16 O4	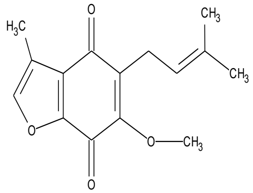	
Ubiquinone-Q8	16055	0.437	-42.686	1	C49 H74 O4	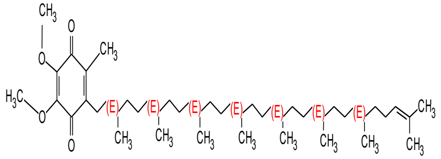	[B] Acetobacter aurantius
2-Hydroxy-5-methoxy-3-(8'Z, 11'Z,14'-pentadecatrien)yl-1,4-benzoquinone	22501	0.437	-36.910	1	C22 H30 O4	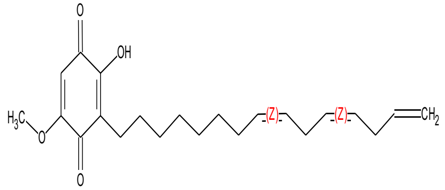	[Pl] etiolated Sorghum seedlings

2,6-Dimethoxy-1,4-benzoquinone	9893	0.437	-21.729	1	C8 H8 O4	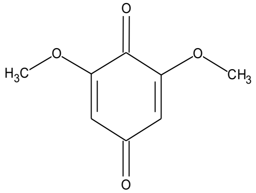	[Pl] widespread in woody tissues; [F] marine fungus Dendryphiella salina [Pl] Lannea nigritana
Saframycin-A	2860	0.436	-63.633	4	C29 H30 N4 O8	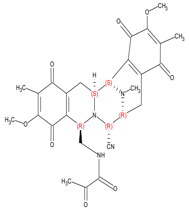	[B] Streptomyces lavendulae 314 FERM-p 3218, NRRL 11002
2,3-Dihydroxy-5-methyl-1,4-benzoquinone	1029	0.435	6.987	1	C7 H6 O4	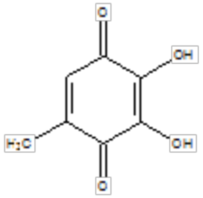	[F]Aspergillus fumigatus
Deflectin 2a	1371	0.435	-53.664	2	C24 H30 O5	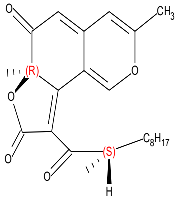	[F] Aspergillus deflectus
Dnacin-B	12091	0.433	-77.514	1	C19 H24 N4 O5	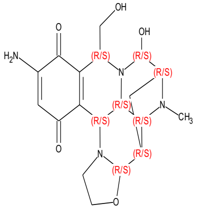	[B] Actinosynnema pretiosum C-14482, Nocardia sp. C-14482 (N-1001) (ATCC 31309, FERM-p 4130, IFO 13725) and its mutant (N-1020) (ATCC 31487, FERM-p 4779, IFO 13887)

Asterriquinone CT1	20524	0.432	-68.532	1	C32 H26 N2 O4	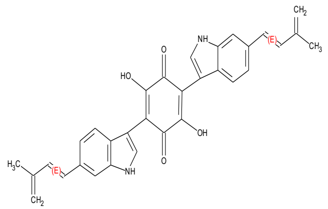	[F] Aspergillus sp., Humicola sp., Botryotrichum sp.
2-Hydroxy-3-methyl-5-methoxy-p-benzoquinone	37545	0.432	-30.459	1	C8 H8 O4	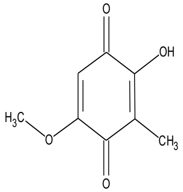	[F] Antrodia salmonea
2,3-Dimethoxy-5-methyl-6-IX,X-tetrahydrofarnesylfarnesyl-geranyl-geranyl-1,4-benzoquinone	4947	0.431	-38.212	1	C64 H102 O4	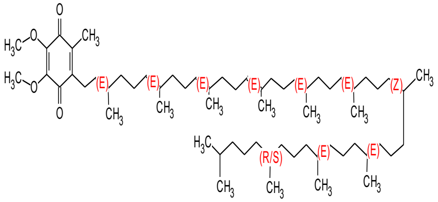	[F] Chaetomium funicola js 525
1,4-Naphthoquinone	14927	0.430	-19.067	2	C10 H6 O2	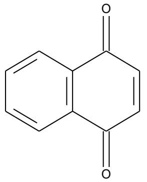	husks of unripe fruit of Juglans nigra 1 and J. regia1 (Juglandaceae). It is known2 that 1 is an intermediate, derived from shikimate, in the biosynthesis of juglone, the characteristic quinone
3,4,3',4'-Bisdehydroxanthomegnin	5992	0.429	-65.174	3	C30 H18 O12	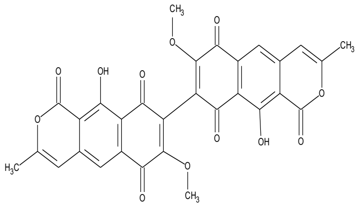	[F] Nannizzia cajetane uamh 3325

Hydroxybreviquinone	14917	0.428	-37.201	1	C15 H16 O5	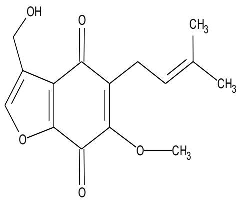	
Isocochliodinol	1871	0.428	-66.857	1	C32 H30 N2 O4	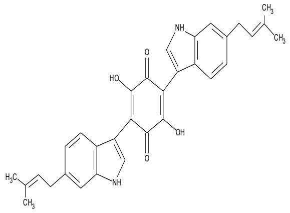	[F] Chaetomium murorum
Neocochliodinol	2359	0.427	-63.055	1	C32 H30 N2 O4	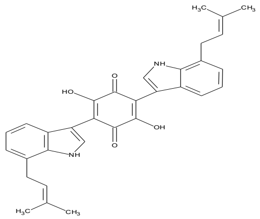	[F] Chaetomium murorum, C. amygdalisporum
Altersolanol-E	11434	0.427	-36.332	3	C16 H16 O8	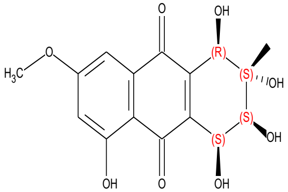	[F] Alternaria solani
Homotrichione	10436	0.427	-96.006	2	C19 H18 O8	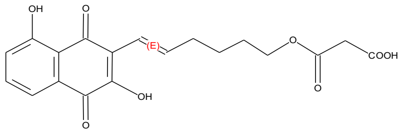	[F] Trichia floriformis, Metatrichia vesparium

Cochliodinol	1293	0.426	-63.024	1	C32 H30 N2 O4	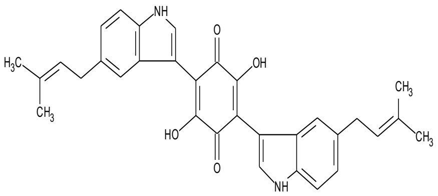	[F] Chaetomium globosum, Chaetomium cochlioides
neo-Asterriquinone	13064	0.426	-61.422	1	C32 H30 N2 O4	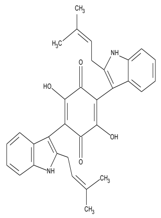	[F] Aspergillus terreus IFO 6123, Humicola sp., Botryotrichum sp.
Asterriquinone CT2	20525	0.425	-67.059	1	C32 H28 N2 O4	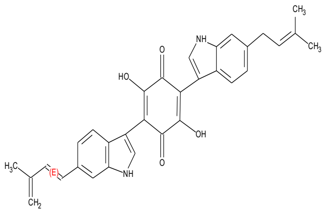	[F] Aspergillus sp., Humicola sp., Botryotrichum sp.
ent-Astropaquinone C	42285	0.425	-38.498	3	C16 H16 O6	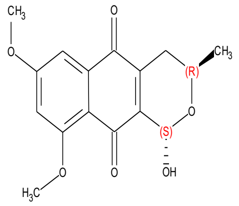	[F] marine Torula herbarum
Asterriquinone SU-5501	22935	0.425	-57.941	1	C32 H30 N2 O4	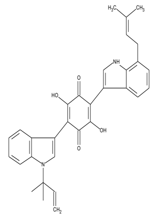	[F] Aspergillus candidus

3,3',8,8'-Tetramethoxy-6,6'-dimethyl-2,2'-binaphthoquinone	20159	0.424	-57.828	2	C26 H22 O8	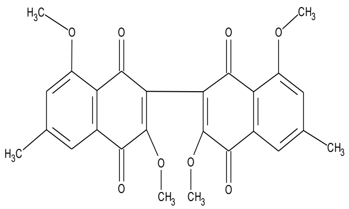	[synthetic]
Cyclospongiaquinone-2	14872	0.422	-25.339	4	C22 H30 O4	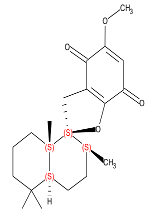	
Astropaquinone C	39516	0.420	-40.496	3	C16 H16 O6	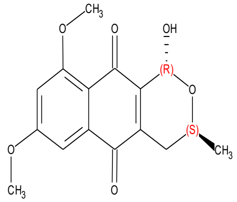	[F] freshwater fungus, Astrosphaeriella papuana YMF 1.01181
2,5-Dihydroxy-3-phenyl-6-(3,4,5-trihydroxyphenyl)-1,4-benzoquinone	29609	0.420	-53.375	1	C18 H12 O7	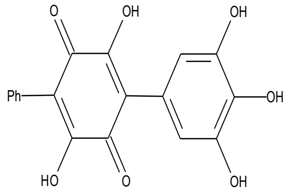	[F] Fusarium sp., Stilbella sp. strain 1586
Astropaquinone A	39514	0.417	-37.230	2	C16 H16 O5	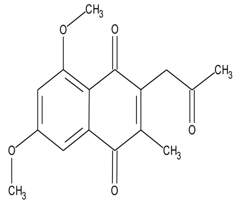	[F] freshwater fungus, Astrosphaeriella papuana YMF 1.01181

Polygonaquinone	10701	0.414	-41.090	1	C28 H48 O4	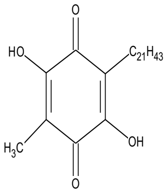	
Maesaquinone	10530	0.411	-41.492	1	C26 H42 O4	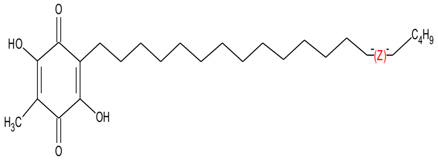	as a monoacetate in fresh fruit, bark and leaves of Maesa lanceolata 55; 70 (n = 9) in aerial parts of M. lanceolata244 (Myrsinaceae). - thomson
Mavioquinone	2171	0.408	-20.632	1	C29 H50 O3	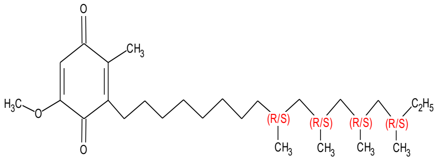	[B] Mycobacterium avium
2,5-Dimethoxy-3,6-dimethyl-1,4-benzoquinone	1039	0.407	-16.126	1	C10 H12 O4	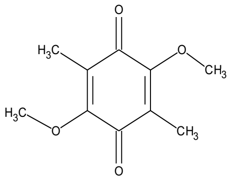	[F] Nectria coryli
Xanthomegnin	5862	0.406	-67.072	4	C30 H22 O12	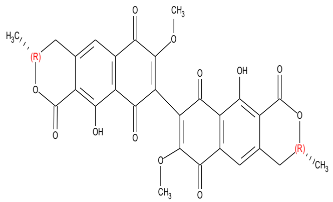	[F] endophytic Coniothyrium sp. CAFT93 [F] Aspergillus ochraceus, A.melleus, A.sulphureus, Penicillium viridicatum, P.cyclopium, Trichophyton rubrum, T. megnini, T. violaceum,

6-Ethyl-1,4-naphthoquinone	14930	0.406	-20.148	2	C12 H10 O2	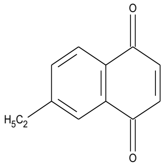	
ent-Astropaquinone B	42284	0.405	-33.234	3	C17 H18 O6	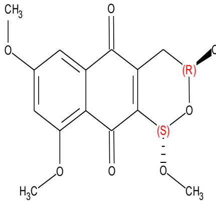	[F] marine Torula herbarum
3-Hydroxythymoquinone	9966	0.405	-17.730	1	C10 H12 O3	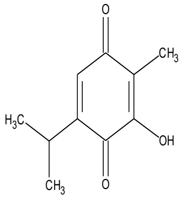	[F] aerial parts of Blumea gariepina (Compositae) se thomson
Bryebinalquinone	14909	0.404	-48.633	1	C18 H14 O8	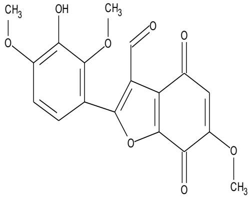	
Przewaquinone F	15550	0.404	-45.005	3	C18 H16 O5	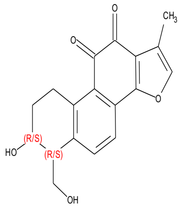	

Epicochlioquinone-A	9257	0.404	-51.765	4	C30 H44 O8	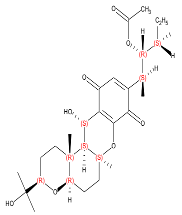	[F] Stachybotrys bisbyi SANK 17777 (FERM bp 3341)
Tauranin	3629	0.403	-27.041	4	C22 H30 O4	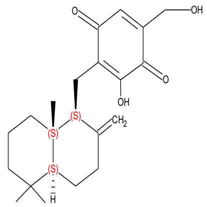	[F] Oospora auranthiaca
Dihydrocyperaquinone	10346	0.403	-29.745	1	C14 H12 O4	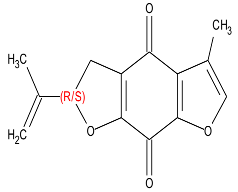	
Hydroxylagopodin B	1817	0.402	-46.191	1	C15 H18 O5	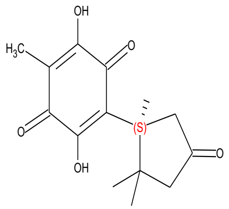	[F] Coprinus macrorhizus-microsporus
O-Methylpulviquinone-A	20997	0.401	-79.567	2	C23 H14 O9	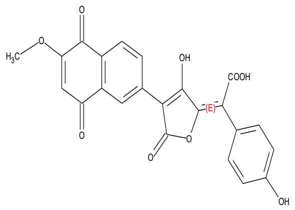	[artifact?]

Hydroxycyperaquinone	10440	0.399	-31.276	1	C14 H10 O5	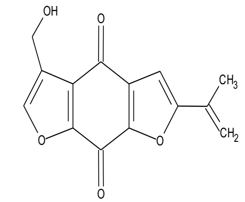	in underground parts of Mariscus ligularis,17 Cyperus surinamensis17 and other Cyperus Spp.174 (all Cyperaceae). - thomson
5-Chlorodermorubin	3070	0.397	-62.698	3	C17 H11 Cl1 O8	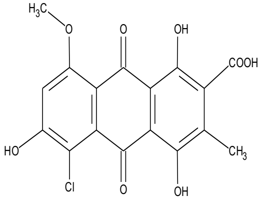	[F] basidiomycete; Dermocybe sanguinea; Dermocybe semisanguinea
3-Hexyl-2-hydroxy-6-pentylbenzoquinone	33535	0.396	-19.153	1	C17 H26 O3	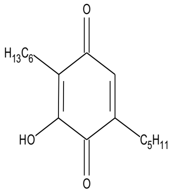	[B] Pseudomonas sp. Ki19
Ilimaquinone	14869	0.396	-38.785	4	C22 H30 O4	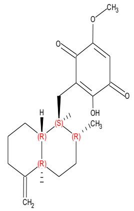	in Spongia,133 Hyatella,125 Smenospongia,126 Fenestraspongia, 128 Fasciospongia,254 and Dactylospongial27.251 spp. sponges; - Thomson
2,5,7-Trimethoxy-naphthoquinone	28191	0.394	-38.984	2	C13 H12 O5	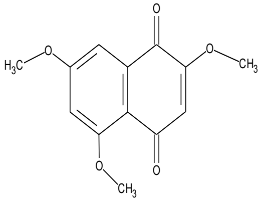	[B] Streptomyces sp. 12396

6-Hydroxy-5-methoxy-2-methyl-1,4-naphthoquinone	10042	0.394	-25.287	2	$C_{12}H_{10}O_4$	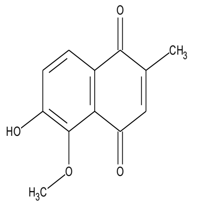	heartwood of Diospyros celebica89 (Ebenaceae). Thomson
6-Methyl-1,4-naphthoquinone	10045	0.393	-19.726	2	$C_{11}H_8O_2$	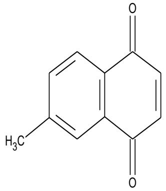	[F] Marasmius graminum, Marasmius gramineum
Cassumunaquinone 2	15055	0.391	-48.171	2	$C_{20}H_{18}O_6$	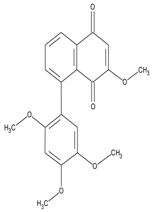	thomson
Astropaquinone B	39515	0.391	-31.816	3	$C_{17}H_{18}O_6$	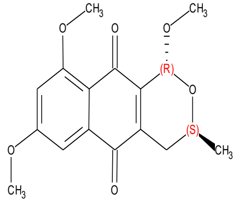	[F] freshwater fungus, Astrosphaeriella papuana YMF 1.01181
Safracin A	2850	0.390	-56.250	4	$C_{28}H_{36}N_4O_6$	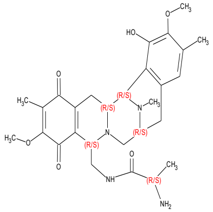	[B] Pseudomonas fluorescens a2-2 (FERM-bp 14, FERM-p 5618 IFO 14128). p. fluorescens p 321

Tanshindol B	15547	0.388	-39.278	3	C18 H16 O5	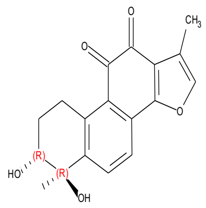	
Ubiquinone Q3	15428	0.387	-31.407	1	C24 H34 O4	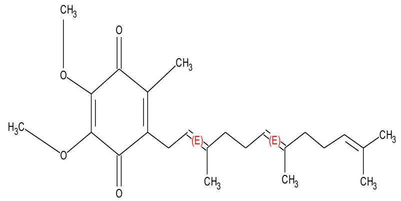	
Ubiquinone Q2	16030	0.386	-28.080	1	C19 H26 O4	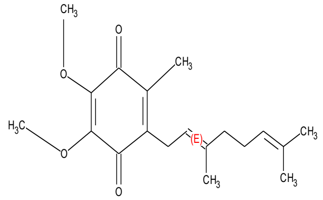	
Mimocin	2221	0.385	-42.854	2	C15 H14 N2 O5	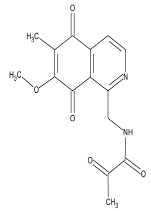	[B] Streptomyces lavendulae 314 (FERM-p 3218, NRRL 11002)
Bostrycin	555	0.384	-48.176	2	C16 H16 O8	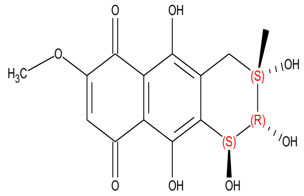	[P] [F] Bostrychonema alpestre, Polygonium filiforme, Nigrospora oryzae, Alt.eichorniae, Arthrinium phaeospermum

2-O,8-O-Dimethyljavanicin	4965	0.384	-42.888	2	C16 H16 O7	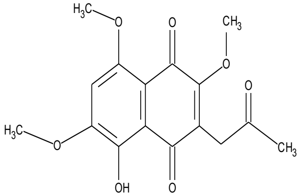	[F] Fusarium solani; semisynth.
Prezwaquinone	15545	0.383	-34.172	3	C18 H16 O4	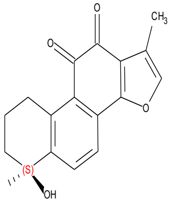	
Ventiloquinone K	15194	0.382	-41.859	3	C17 H18 O7	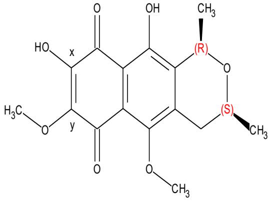	thomson
Tecomaquinone-I	15231	0.381	-31.533	3	C30 H24 O4	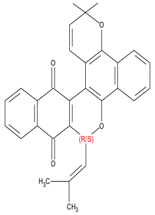	
Tanshindol C	15548	0.380	-42.158	3	C18 H16 O5	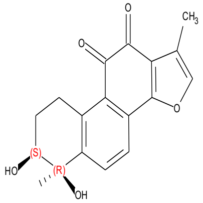	

5,6-Dihydroxy-2,3-dimethyl-1,4-benzoquinone	10007	0.380	-18.379	1	C8 H8 O4	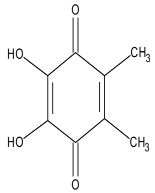	
Proansamycin B-M1	4661	0.378	-85.874	2	C35 H47 N1 O10	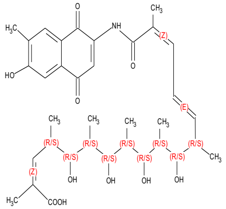	[B] Nocardia mediterranei f 1/24
(3R)-Scabequinone	20458	0.378	-29.267	2	C15 H16 O4	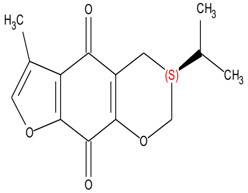	
2-Methyl-1,4-naphthoquinone	14928	0.376	-19.048	2	C11 H8 O2	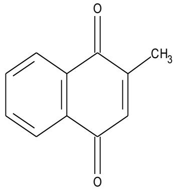	husks of unripe fruit of Juglans nigra 1 and J. regia1 (Juglandaceae). It is known2 that 1 is an intermediate, derived from shikimate, in the biosynthesis of juglone, the characteristic quinone
Helicobasidin	6418	0.374	-37.603	1	C15 H20 O4	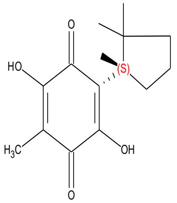	[F] Helicobasidium mompa

Rugulin	13568	0.374	######	4	C32 H24 O10	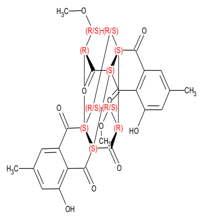	[F] Penicillium rugulosum
Javanicin 8-methyl ether	4427	0.373	-38.617	2	C16 H16 O6	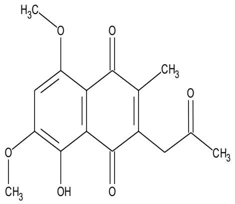	[F] Fusarium moniliforme mrc 602 f. solani
(5S,7S)-5,6,7,8-Tetrahydro-5,7-dihydroxy-2-methoxy-7-methyl-1,4- anthraquinone	8843	0.372	-57.027	3	C16 H16 O7	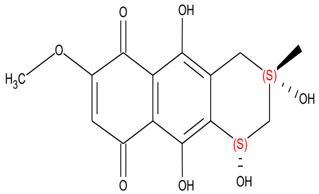	[F] Dermocybe sp.
b-Methylpyrano-1,4-naphthoquinone	17337	0.371	-26.794	3	C14 H10 O3	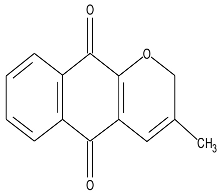	
1,6-Dimethyl-7-methoxyisoquinoline-5,8-quinone	15553	0.371	-27.156	2	C12 H11 N1 O3	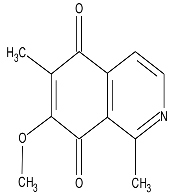	

1,4-Naphthoquinone-8-hydroxy-3-[(3S)-acetoxy]butyric acid	25840	0.367	-58.731	2	C16 H14 O7	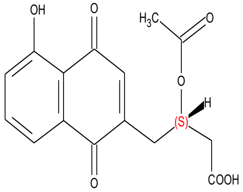	[B] Streptomyces coelicolor mutant A3(2)
1,4-Dihydroxy-2,5-dimethoxy-7-methylanthraquinone	7886	0.365	-65.368	3	C17 H14 O6	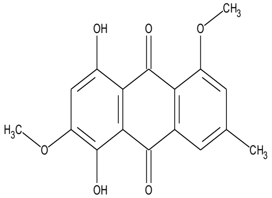	[F] Dermocybe WAT 22963
Diboviquinone-4,4	14887	0.365	-70.189	1	C52 H70 O8	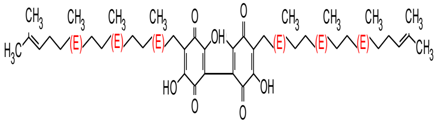	
Diboviquinone-3,4	14886	0.365	-68.183	1	C47 H62 O8	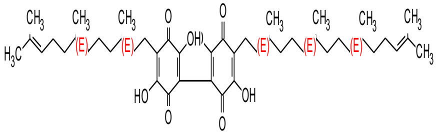	
Granaticin methyl ester	6411	0.364	-57.087	4	C23 H24 O11	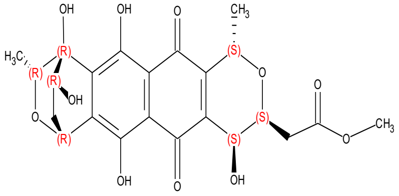	[B] Streptomyces lateritius (zimet 43646)

Cassumunaquinone 1	15054	0.363	-43.505	2	C19 H16 O5	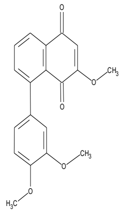	thomson
Stenocarpoquinone B	15047	0.363	-31.315	2	C15 H14 O4	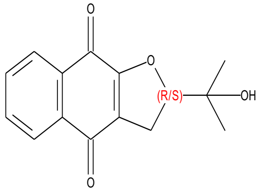	
Dicinnaquinone	7266	0.362	######	2	C14 H8 N2 O10	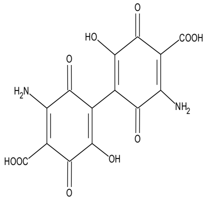	[B] Streptomyces griseoflavus ssp. thermodiastaticus Tue 2486
WS 5995-A	956	0.356	-37.453	4	C19 H12 O6	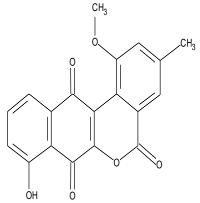	[B] Streptomyces auranticolor 5995 (FERM-p 5365)
Murrayaquinone B	15586	0.356	-28.424	1	C19 H19 N1 O3	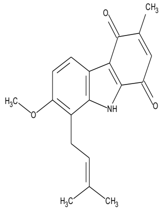	in roots and stem bark of Murraya koenigii; THOMSON

8-Hydroxy-2,5,6-trimethoxy-7-(2-oxopropyl)-1,4- naphthoquinone	5045	0.355	-49.145	2	C16 H16 O7	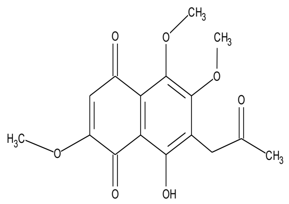	[F] Fusarium solani
Hexahydromenaquinone MK-9(II,III,IX-H6)	10556	0.353	-33.702	2	C56 H86 O2	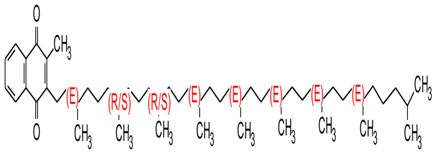	[B] Archaebacterium, Natonobacterium, Actinobacillus, Pasteurellaceae
3,3'-Bidiomelquinone	15110	0.353	-47.378	2	C24 H18 O8	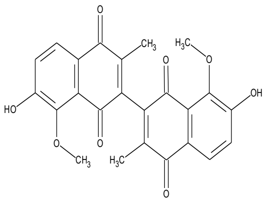	
Menaquinone MK-9(II,III-H4)	5507	0.351	-36.878	2	C56 H84 O2	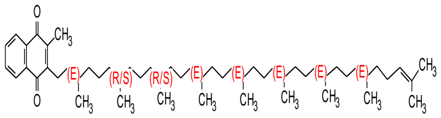	[B] Archaebacterium, Natonobacterium, Actinobacillus, Pasteurellaceae, Glycomyces rutgersensis
2-Methoxycarbonyl-3-prenyl-1,4-naphthoquinone	15038	0.350	-29.671	2	C17 H16 O4	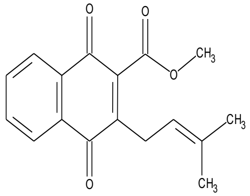	

(3R)-Hydroxyscabequinone	20460	0.350	-34.770	2	C15 H16 O5	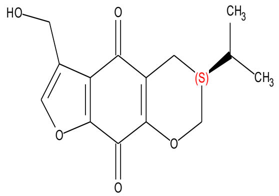	
Diboviquinone-4,4	16595	0.347	-46.134	1	C52 H72 O8	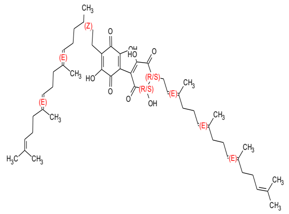	[F] Gomphidius rutilus
Ventiloquinone-G	8774	0.347	-47.016	3	C15 H14 O6	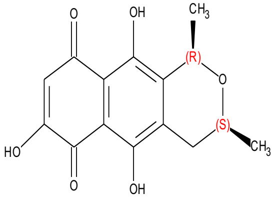	[Pl] Ventilago maderaspatana
g-Naphthocyclinone	2030	0.346	-70.769	3	C35 H30 O14	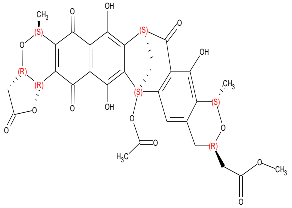	[B] Streptomyces arenae Tue 495
Conicaquinone	20462	0.345	-37.383	1	C13 H8 O5	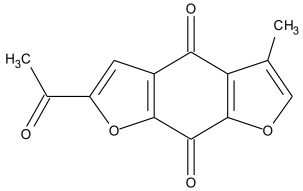	

Rhodoquinone-9	10744	0.344	-47.320	1	C53 H81 N1 O3	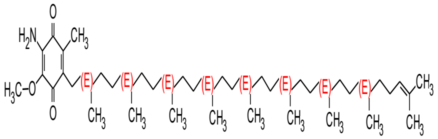	
Chlorobiumquinone	10224	0.344	-45.771	2	C46 H62 O3	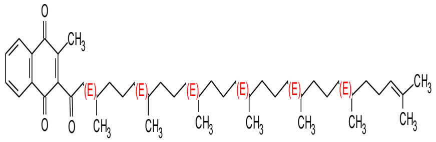	
Isomarticin	3348	0.344	-68.493	2	C18 H16 O9	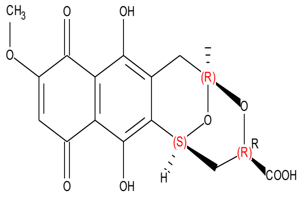	[F] Fusarium martici, Fus.solani, Fus.javanicum, Neocosmospora vasinfecta
Rhodoquinone-10	10743	0.344	-50.663	3	C58 H89 N1 O3	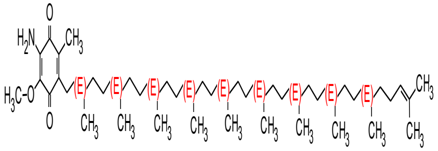	
Rhodoquinone-8	17479	0.343	-42.665	1	C48 H73 N1 O3	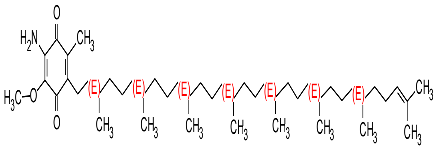	[B] Brachymonas denitrificans

Mycochrysone	10599	0.342	-54.354	2	C20 H12 O7	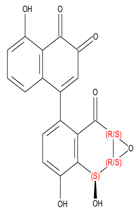	[F] Discomycete
Menaquinone MK-8(II,III-H4)	10553	0.341	-33.893	2	C51 H76 O2	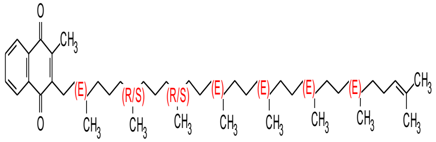	[B] Archaebacterium, Natonobacterium, Actinobacillus, Pasteurellaceae
8-Hydroxy-2,5-dimethoxy-6-methyl-7-(2-oxopropyl)-1,4-naphthoquinone	5046	0.340	-37.377	2	C16 H16 O6	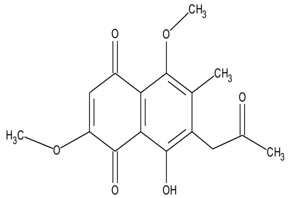	[F] Fusarium solani; semisynth.
Hexahydromenaquinone MK-9(II,III,VIII-H6)	10558	0.339	-35.051	2	C56 H86 O2	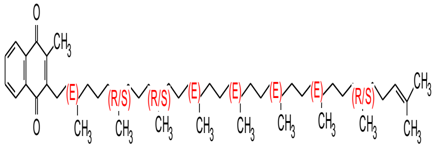	[B] Archaebacterium, Natonobacterium, Actinobacillus, Pasteurellaceae
Marticin	2168	0.339	-69.380	3	C18 H16 O9	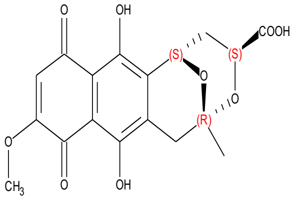	[F] Fusarium martici, Fus.solani, Fus.javanicum, Neocosmospora vasinfecta

Demethylmenaquinone	10309	0.339	-35.847	2	C45 H62 O2	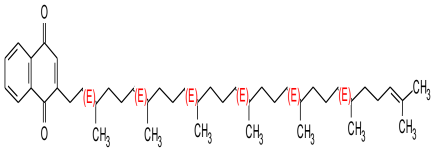	[B] Actinobacillus, Pasteurellaceae
3,4-Dehydroxanthomegnin-diacetat	7946	0.338	-73.263	3	C34 H24 O14	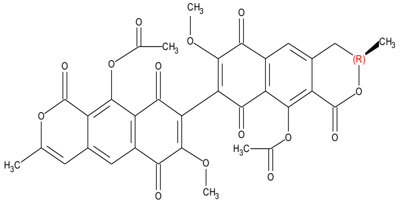	[semisynth.]
Hibiscoquinone B	15170	0.337	-29.661	2	C14 H14 O4	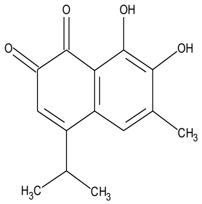	
Griseusin B	1766	0.336	-55.097	3	C22 H22 O10	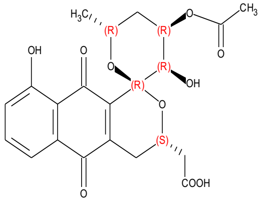	[B] Streptomyces griseus k-63
2-Dodecyl-naphthoquinone	20108	0.335	-20.695	2	C22 H30 O2	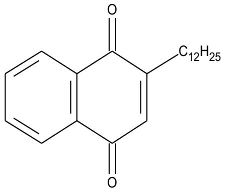	[synthetic]

2-Hexylnaphthoquinone	20198	0.334	-19.828	2	C16 H18 O2	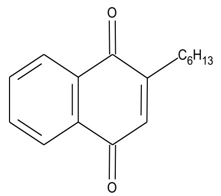	[synthetic]
Methionaquinone-7	24552	0.334	-42.298	2	C46 H64 O2 S1	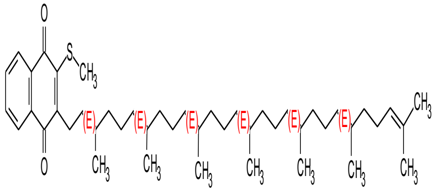	[B] sulfur-turf mats; Thermoplasma acidophilum HO-62
Purpuromycin	2737	0.334	-83.513	2	C26 H18 O13	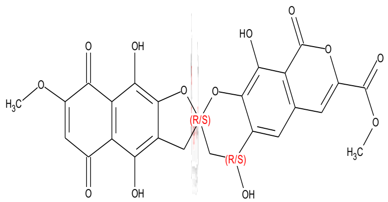	[B] Actinoplanes ianthinogenes, Actinoplanes ianthogenes-octamycini
Kwanzoquinone B	39183	0.330	-24.552	3	C18 H14 O4	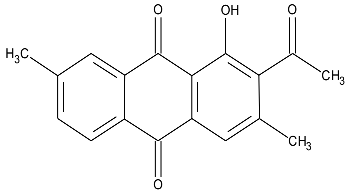	[Pl] Homerocallus fulva
K-82 A	2042	0.330	-53.020	2	C22 H14 N4 O4	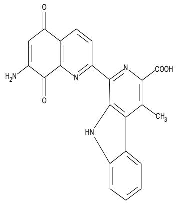	[B] Streptomyces lavendulae C-22030 S. lavendulae k-82 (FERM-p 4479, IFO 13837)

Demethylcyperaquinone	10308	0.329	-26.573	1	C13 H8 O4	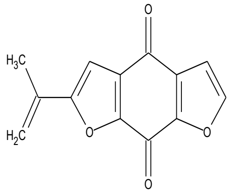	
d-Actinorhodin	7103	0.328	-86.423	4	C33 H20 O16	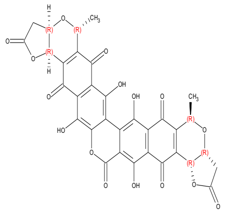	[B] Streptomyces coelicolor
1-Deoxybostrycin	2970	0.328	-45.934	3	C16 H16 O7	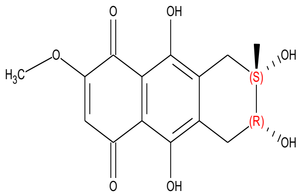	[F] Alternaria eichhorniae
Nepenthon A	24205	0.327	-44.647	2	C13 H10 O7	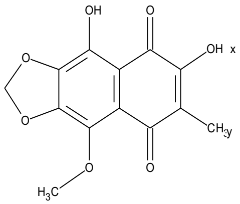	[Pl] Nepenthes rafflesiana
II-Dihydromenaquinone-10, MK-10(II-H2)	10562	0.326	-42.789	2	C61 H90 O2	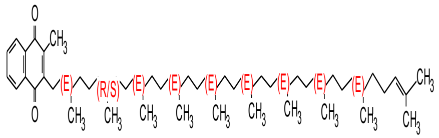	[B] thomson

II-Dihydromenaquinone-7	10561	0.325	-32.780	2	C46 H66 O2	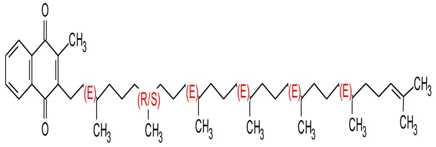	[B] Halococcus morrhuae,19 Natronobacterium gregori;2S _ Thomson
Methylenediboviquinone-3,4	16916	0.325	-64.906	1	C50 H68 O8	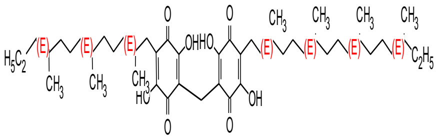	[F] Gomphidius rutilus
Menaquinone MK-8(II-H2)	10554	0.325	-37.138	2	C51 H74 O2	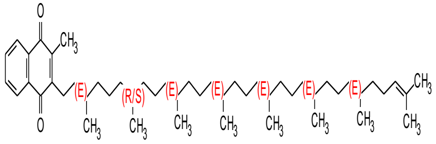	[B] Archaebacterium, Natonobacterium, Actinobacillus, Pasteurellaceae
Quinone A	5670	0.325	-30.598	2	C51 H74 O2	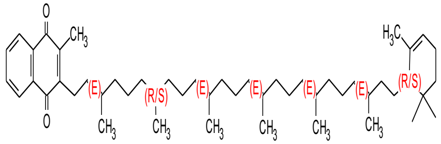	[B] Nocardia brasiliensis
Griseusin A	4379	0.323	-57.817	4	C22 H20 O10	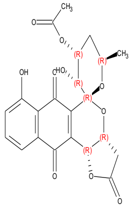	[B] Streptomyces griseus k-63

Fusarubin methyl acetal	15178	0.323	-47.160	3	C16 H16 O7	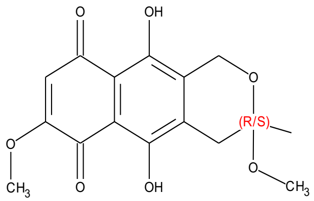	[F]fusarium - thomson	
Fusarubin	10411	0.323	-51.815	3	C15 H14 O7	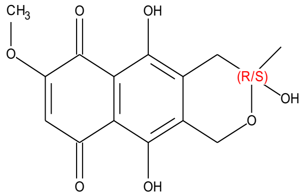	[F] Fusarium solani, Fusarium decemcellulare, Fus.martici-pisi, Fus.javanicum, Neocosmospora vasinfecta	
7-Hydroxyroyleanone	10812	0.322	-18.413	3	C20 H28 O4	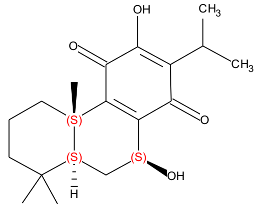		
Lurlenic acid	21505	0.322	-61.826	1	C30 H44 O8	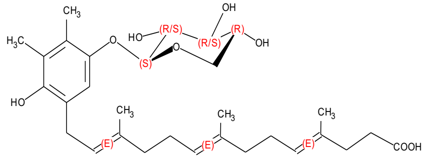	[A] Chlamydomonas allensworthii	2
Fusarubin ethyl acetal	15179	0.322	-46.204	3	C17 H18 O7	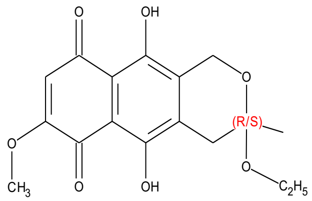	[F]fusarium - thomson	

Aclacinomycin B	5080	0.322	-80.639	4	C42 H51 N1 O15	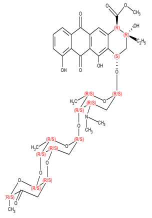	[B] Streptomyces galilaeus ma144-m1 (ATCC 31133, FERM-p 2455)
Deoxybryaquinone	14908	0.321	-38.351	1	C17 H12 O6	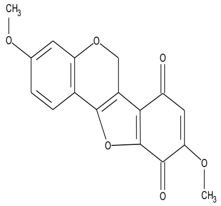	
Murrayaquinone A	15585	0.319	-26.316	1	C13 H9 N1 O2	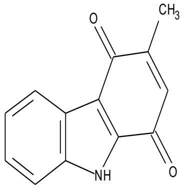	in roots and stem bark of Murraya koenigii; THOMSON
Menaquinone MK-5(V-H2)	10550	0.319	-28.070	2	C36 H50 O2	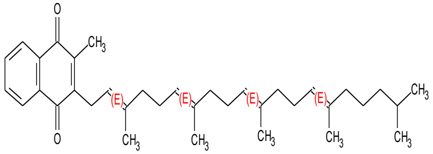	[B] Archaebacterium, Natonobacterium, Actinobacillus, Pasteurellaceae
Menaquinone	10564	0.319	-33.436	2	C41 H56 O2	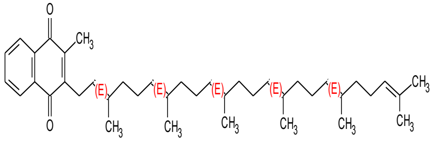	[B] marine Bacterium Hel 21

2-Methyl-3-VI,VII-tetrahydroheptaprenyl-1,4-naphthoquinone	3996	0.318	-29.782	2	C46 H68 O2	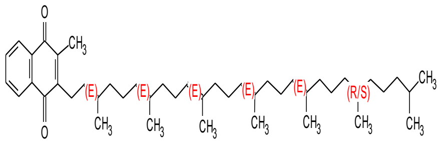	[B] Thermoleophilum album
Menaquinone	10565	0.318	-38.291	2	C56 H80 O2	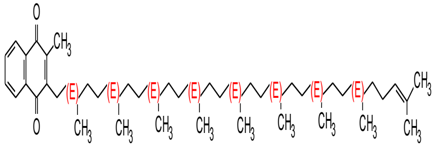	[B] - Actinomadura olivaceus; Actinomadura angiospora - thomson
Menaquinone MK-6(VI-H2)	10551	0.318	-33.744	2	C41 H58 O2	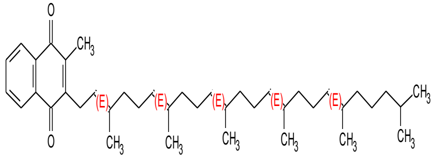	[B] Archaebacterium, Natonobacterium, Actinobacillus, Pasteurellaceae
Bryaquinone	14907	0.317	-44.466	1	C17 H12 O7	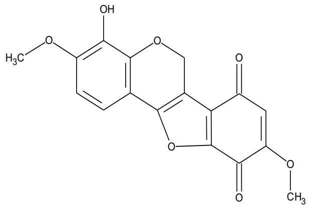	
Solanoquinone	15039	0.316	-21.191	2	C28 H42 O2	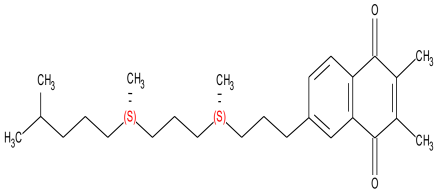	

2-Hydroxy-1,3,4-trimethoxyanthraquinone	15375	0.312	-34.712	3	C17 H14 O6	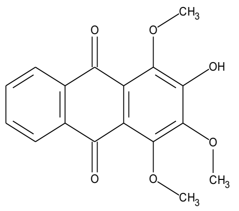	
2-{14-[3-(1,5-Dimethylhexyl)cyclopentyl]-3,7,11-trimethyl-tetradecyl}-3-methyl-1,4-naphthoquinone	7931	0.311	-23.457	2	C41 H66 O2	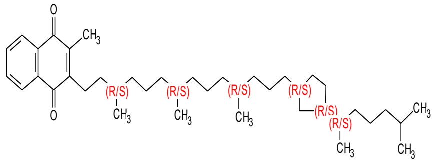	[B] archaebacterium Pyrobaculum organotrophum
2-Hydroxyethyl-3-methyl-1,4-naphthoquinone	26242	0.309	-29.934	2	C13 H12 O3	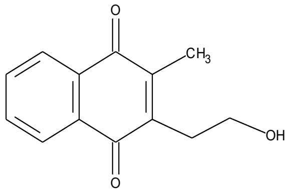	[B] Actinoplanes capillaceus
Safracin B	4705	0.308	-58.138	4	C28 H36 N4 O7	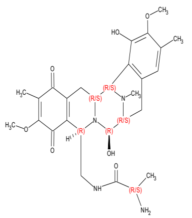	[B] Pseudomonas fluorescens a2-2 (FERM-bp 14, FERM-p 5618 IFO 14128). p. fluorescens p 321
Menaquinone	10563	0.307	-47.008	2	C66 H98 O2	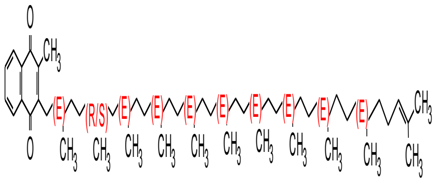	[B] thomson

Cyperaquinone	10286	0.306	-26.592	1	C14 H10 O4	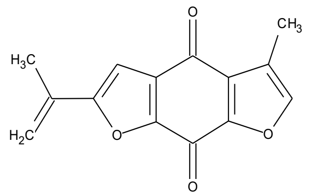	in underground parts of Mariscus ligularis,17 Cyperus surinamensis17 and other Cyperus Spp.174 (all Cyperaceae). - thomson
3-Methoxy-2-methyl-9H-carbazole-1,4-quinone	25203	0.305	-34.090	1	C14 H11 N1 O3	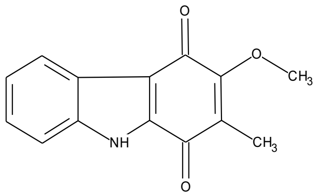	[B] Streptomyces sp. CMU-JT005
Tecomaquinone II	15043	0.305	-41.599	2	C30 H26 O5	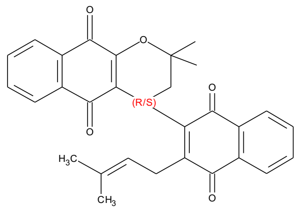	wood of Tabebuia incana302 (Bignoniaceae) and Tectona grandis438 (Verbenaceae). - thomson
Dihydromenaquinone MK-9(II-H2)	10560	0.304	-40.854	2	C56 H82 O2	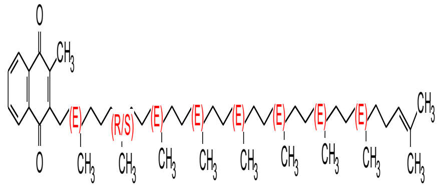	[B] Archaebacterium, Natonobacterium, Actinobacillus, Pasteurellaceae
1,6,8-Trihydroxy-3-methylanthraquinone-2-carboxylic acid	2967	0.304	-50.379	3	C16 H10 O7	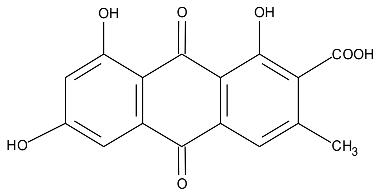	[L] [F] Aspergillus sp., Nephromopsis endocrocea, Lichen, Claviceps purpurea, Asp. aculeatus, Cetraria cucullata, Cortinarius armillatus

Murrayaquinone C	15587	0.303	-17.405	1	C24 H27 N1 O3	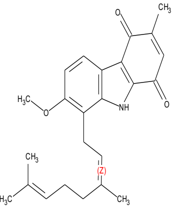	in roots and stem bark of Murraya koenigii; THOMSON
O-Methylherbarin	4587	0.303	-37.443	3	C17 H18 O6	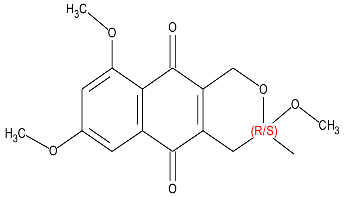	[Y] Torula herbarum
6-O-Methyldermorubin	3085	0.302	-50.227	3	C18 H14 O8	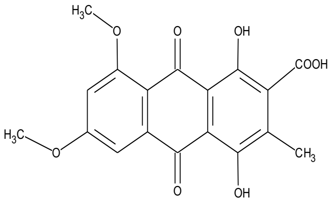	[F] Cortinarius armillatus; Cortinarius miniatopus
Dicyperaquinone D	14921	0.302	-59.503	1	C28 H20 O10	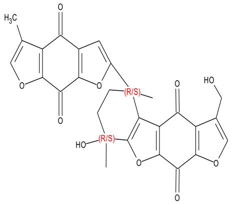	
2,3-Dimethyl-1,4-naphthoquinone	14931	0.302	-18.179	2	C12 H10 O2	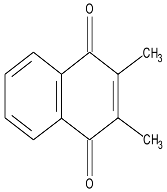	

Rotundiquinone	15064	0.301	-31.370	2	C22 H14 O6	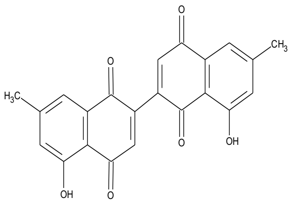	
Olivovariin	2493	0.300	-67.062	2	C14 H12 O7	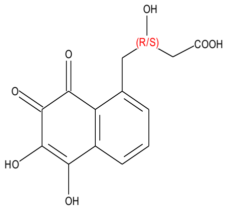	[B] Actinomadura olivovariabilis
2,3-Didodecyl-naphthoquinone	20107	0.300	-23.320	2	C34 H54 O2	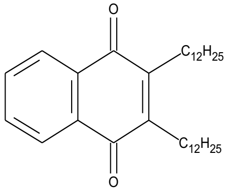	[synthetic]
Dicyperaquinone A	14918	0.300	-46.399	1	C28 H18 O8	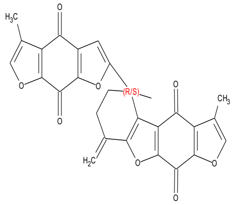	
2-Hexyl-3-methyl-naphthoquinone	31966	0.300	-19.064	2	C17 H20 O2	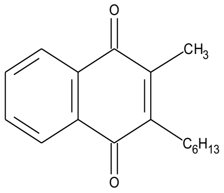	[synthetic]

Benzanthrin A	512	0.300	-55.588	4	C35 H42 N2 O9	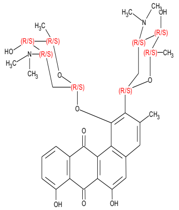	[B] Nocardia lurida
Menaquinone MK-7	9469	0.299	-34.460	2	C46 H64 O2	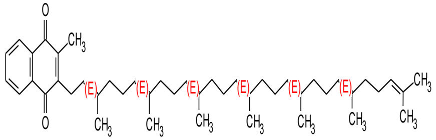	[B] Archaebacterium, Natonobacterium, Actinobacillus, Pasteurellaceae, [B] B.brevis, B.subtilis, Mycobacterium sp., marine Streptomyces sp. B 5530, B7939
Menaquinone MK-7(VII-H2)	10552	0.299	-33.620	2	C46 H66 O2	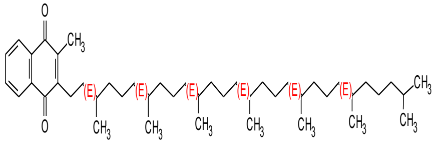	[B] Archaebacterium, Natonobacterium, Actinobacillus, Pasteurellaceae
Menaquinone MK-8(VIII-H2)	10555	0.299	-35.637	1	C51 H74 O2	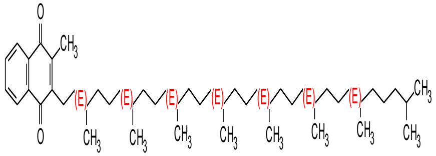	[B] Archaebacterium, Natonobacterium, Actinobacillus, Pasteurellaceae
2-Methyl-3-(g,g-dimethylallyl)-1,4-naphthoquinone	9920	0.299	-19.694	2	C16 H16 O2	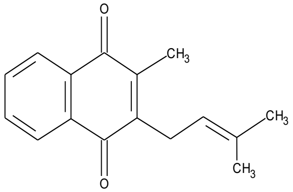	

Menaquinone MK-8	5506	0.298	-37.986	2	C51 H72 O2	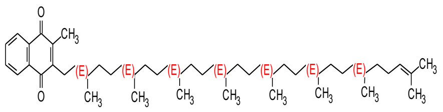	[B] Archaebacterium, Natonobacterium, Actinobacillus, Pasteurellaceae
4,7-Dihydro-6-methoxy-2,5-dimethyl-2H-isoindole-4,7-dione	1111	0.298	-40.588	1	C11 H11 N1 O3	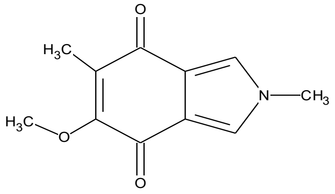	[An] sponge Reniera sp.
Dicyperaquinone B	14919	0.297	-48.231	1	C28 H18 O8	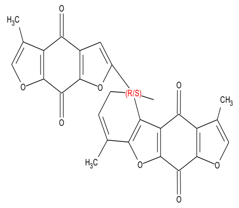	
6,7-Diethoxy-2-methylnaphthoquinone	20227	0.297	-26.641	2	C15 H16 O4	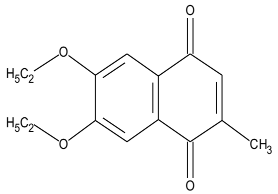	[synthetic]
Dicyperaquinone E	14922	0.294	-66.324	1	C28 H20 O11	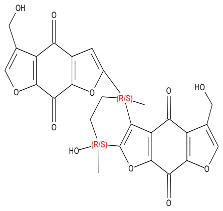	

Dicyperaquinone C	14920	0.294	-53.517	1	C28 H20 O9	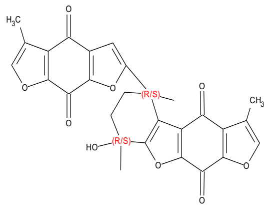	
2,3,6-Trimethyl-1,4-naphthoquinone	17215	0.293	-18.447	2	C13 H12 O2	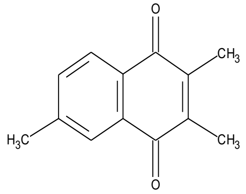	
Phylloquinone	10684	0.289	-20.083	2	C31 H46 O2	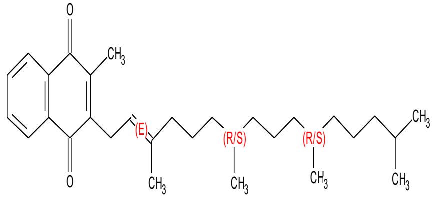	[B] thomson
Menaquinone-10 (II-, III-H4)	20213	0.287	-42.523	2	C61 H92 O2	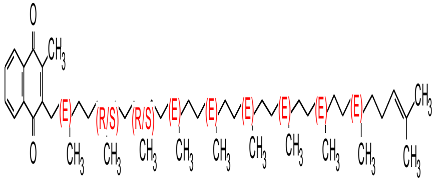	[B] Glycomyces rutgersensis
8-Hydroxy-5,6-dimethoxy-2-methyl-3-(2-oxopropyl)-1,4-naphthoquinone	5047	0.286	-34.032	2	C16 H16 O6	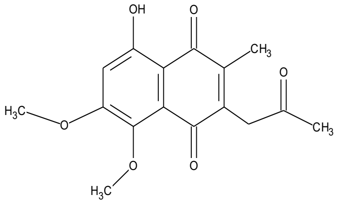	[F] Fusarium solani

(3'R,P)-Anhydropseudophlegmacin-9,10-quinone-1,6',8'-tri-O-methyl ether	23603	0.285	-77.976	3	C33 H28 O10	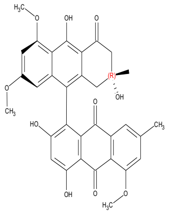	[F] fungus Dermocybe sp.
1,6,8-Trimethoxy-3-propanoylanthraquinone	15370	0.285	-44.703	3	C20 H18 O6	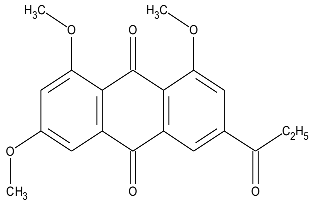	
SM-196-A	8662	0.280	-39.971	4	C20 H20 O5	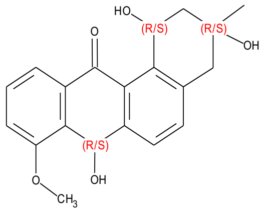	[B] Streptomyces sp
Scorpinone	27343	0.274	-38.141	3	C16 H13 N1 O4	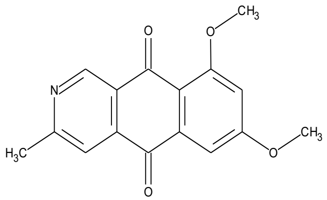	[F] Bispora-like tropical fungus
5,5'-Dihydroxy-7,7'-dimethyl-6,6'-binaphthyl-1,4	20177	0.274	-34.865	2	C22 H14 O6	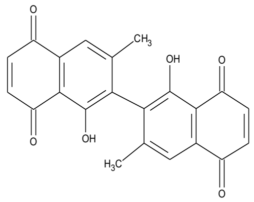	[synthetic]

Herbarin	1787	0.270	-39.840	3	C16 H16 O6	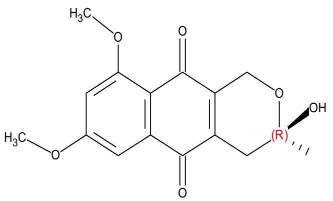	[Y] Torula herbarum [F] endolichenic fungus Corynespora sp. BA-10763 on lichen U. cavernosa
AM 3867-I	1636	0.263	-39.848	3	C18 H16 O6	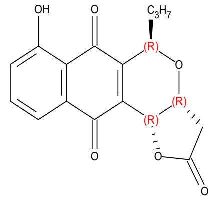	[B] Streptomyces roseofulvus am-3867 (FERM-p 4359), S.roseoflavus
Kalamycin	2044	0.263	-39.738	3	C16 H12 O6	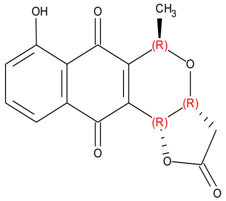	[B] Streptomyces coelicolor, S.tanashiensis
b'-Dihydrodiospyrin	15065	0.261	-36.650	2	C22 H16 O6	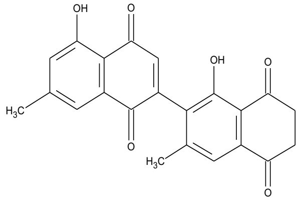	
6-Bromo-5-hydroxy-6-methylnaphthoquinone	20182	0.261	-20.175	2	C11 H7 Br1 O3	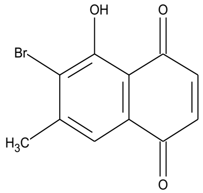	[synthetic]

Lawsone methyl ether	10498	0.259	-27.348	2	C11 H8 O3	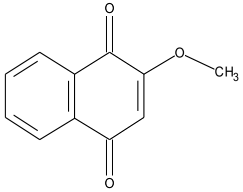	
2,5-Di-(N-(-)-prolyl)-p-benzoquinone	41462	0.258	-78.123	1	C16 H18 N2 O6	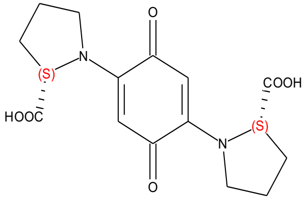	[Pl] coloured nectar of the bird's coca cola tree, Leucosceptrum canum
2-Amino-3-carboxy-1,4-naphthoquinone	24266	0.255	-49.245	2	C11 H7 N1 O4	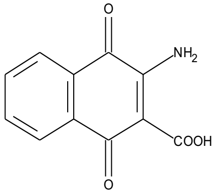	[B] Propionibacterium freudenreichii
Julimycin B-II	10484	0.254	-89.014	3	C38 H34 O14	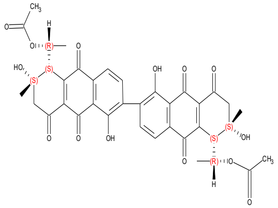	[B] Streptomyces shinodaensis
2,3-Diprenyl-1,4-naphthoquinone	15037	0.253	-19.657	2	C20 H22 O2	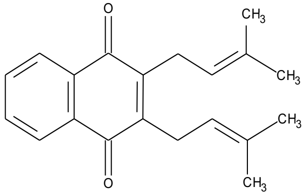	

Phthyocol	9563	0.252	-30.196	2	C11 H8 O3	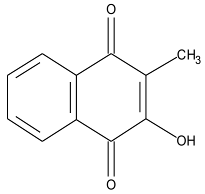	[B] Mycobacterium tuberculosis
Methylmenaquinone MMK-8	19347	0.252	-33.712	2	C52 H74 O2	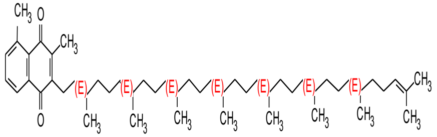	[B] (Archaebacterium) Natonobacterium [B] gregoryi, Actinobacillus, Pasteurellaceae
Dimethylmenaquinone	1443	0.251	-32.909	2	C43 H60 O2	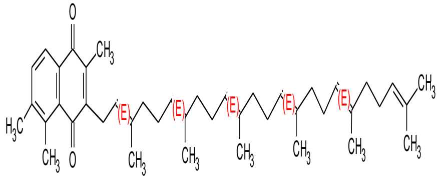	[B] Eubacterium lentum
2-trans-Thermoplasmaquinone	26833	0.251	-35.109	2	C47 H66 O2	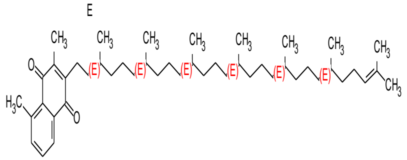	[B] Thermoplasma acidophilum HO-62
F8	5387	0.250	-29.551	2	C14 H12 O4	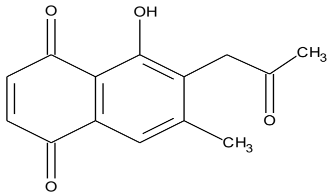	[F] Fungus, Fusarium sp.

Cadalene-1,4-quinone	15164	0.250	-16.559	2	C15 H16 O2	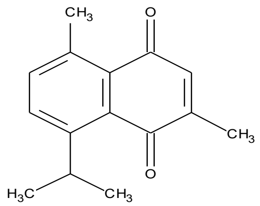	
Methylmenaquinone MMK-8(VIII-H2)	19348	0.250	-35.487	2	C52 H76 O2	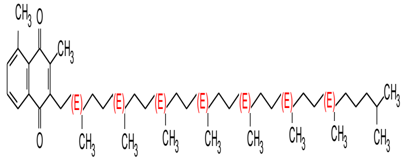	[B] (Archaebacterium) Natonobacterium [B] gregoryi, Actinobacillus, Pasteurellaceae
2-cis-Thermoplasmaquinone	26834	0.250	-35.823	2	C47 H66 O2	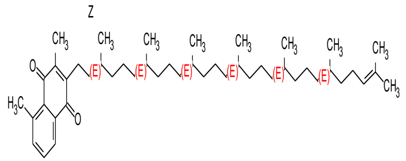	[B] Thermoplasma acidophilum HO-62
2-Hydroxymethyl-1-methoxy-anthraquinone	42431	0.250	-30.275	3	C16 H12 O4	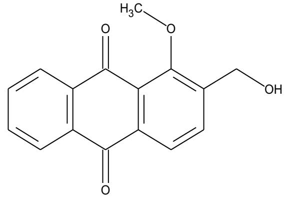	[Pl] Prismatomeris malayana
Sakyomicin A	2861	0.249	-47.919	4	C25 H26 O10	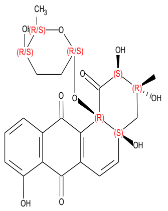	[B] Nocardia sp. 53, M53

Anhydroflavomannin-9,10-quinone-6,6',8'-tri-O-methyl ether	17505	0.246	-65.400	3	C33 H28 O10	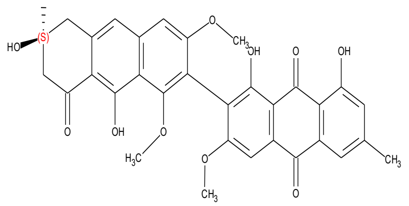	[F] Dermocybe sp. WAT 24274
Pulviquinone-A	17759	0.244	-60.230	2	C22 H12 O9	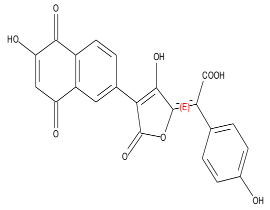	
Dimethylmenaquinone DMMK-8(VIII-H2)	19349	0.242	-35.450	2	C53 H78 O2	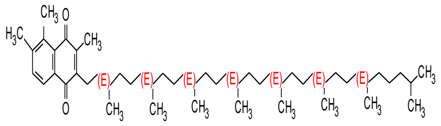	[B] (Archaebacterium) Natonobacterium [B] gregoryi, Actinobacillus, Pasteurellaceae
5,5',6,6'-Tetrahydroxy-2,2'-dimethyl-2,2'-binaphthoquinone	20179	0.242	-37.706	2	C22 H14 O8	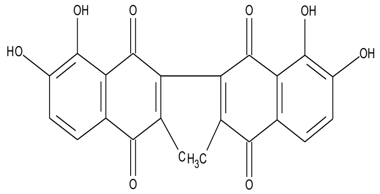	[synthetic]
5,5',8-Trihydroxy-2,2'-dimethyl-6,6'-binaphthoquinone	20165	0.241	-33.242	2	C22 H14 O7	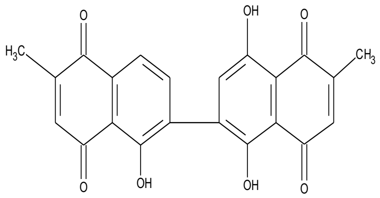	[synthetic]

Dimethylmenaquinone DMMK-8	19350	0.240	-37.081	2	C53 H76 O2	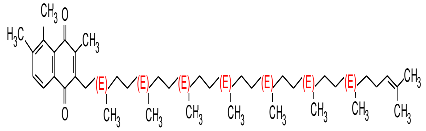	[B] (Archaebacterium) Natonobacterium [B] gregoryi, Actinobacillus, Pasteurellaceae
(+)-Chloromycorrhizin A	3910	0.239	-29.860	1	C14 H14 Cl2 O4	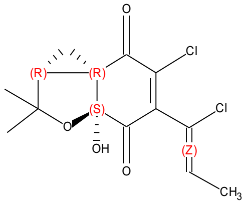	[F] (ascomycete) Lachnum papyraceum + CaBr2, Cochliobolus lunatus, [F] Monotropa hypopitys, Gilmaniella humicola
Streptovaricin U	6766	0.239	-77.178	2	C36 H49 N1 O10	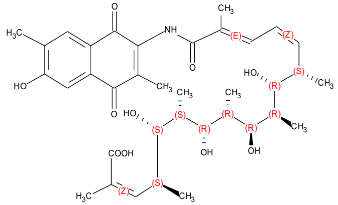	[B] Streptomyces spectabilis
2-Butyryl-1,8-dihydroxy-3-methylanthraquinone	38482	0.238	-27.484	3	C19 H16 O5	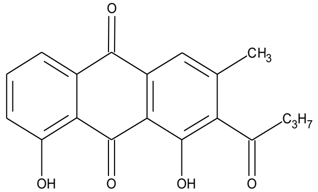	[B] terrestrial Streptomyces sp. Eg5
Sanguinolentaquinone	34718	0.237	-56.263	1	C13 H16 N2 O4	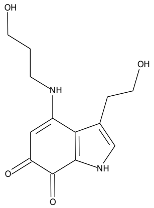	[F] Mycena sanguinolenta

Ventiloquinone B	15196	0.237	-39.473	3	C18 H18 O7	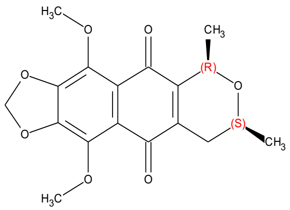	thomson
8-O-Methyl-2-hydroxyjavanicin	5049	0.235	-39.638	2	C15 H14 O7	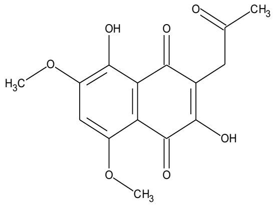	[F] Fusarium oxysporum
Ancistroquinone	15126	0.234	-26.744	2	C12 H10 O5	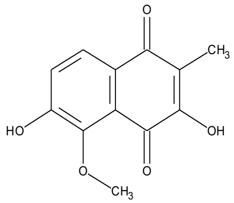	
6,8-Dihydroxy-1-methoxy-3-methylanthraquinone	14237	0.232	-55.987	3	C16 H12 O5	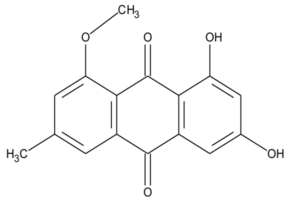	[F] endophytic Coniothyrium sp. CAFT93 [F] fungus Phialophora alba
Dehydroherbarin	4213	0.231	-29.490	3	C16 H14 O5	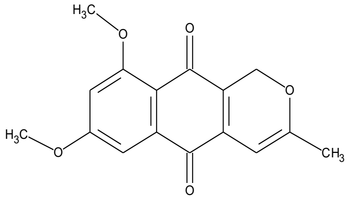	[Y] Torula herbarum

2-Acetylnaphthol[2,3-b]furan-4,9-quinone	15052	0.230	-26.985	2	C14 H8 O4	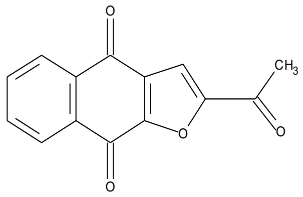	
7-Acetonyl-5-hydroxy-6-methyl-1,4-naphthoquinone	23219	0.229	-30.995	2	C14 H12 O4	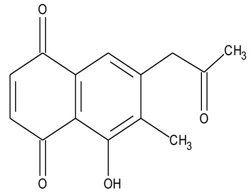	[F] Fusarium sp.
Atrovenetin	5200	0.229	-51.852	3	C19 H18 O6	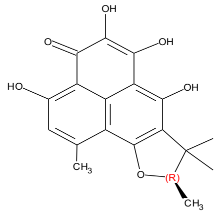	[F] Gremmeniella abietina, Penicillium Herquer, P. atrovetum
(5S,6R,8R,10R)-Sarubicin A	2873	0.226	-35.931	2	C13 H14 N2 O6	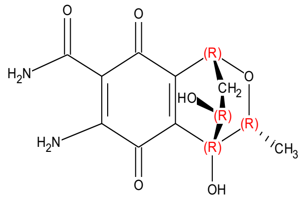	[B] Streptomyces sp. ja 2861, reS. to s. violaceoruber, S. helicus uc-5837
2,7-Dihydroxy-5-methyl-1,4-naphthoquinone	1040	0.226	-42.124	2	C11 H8 O4	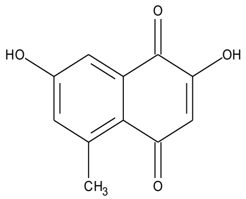	[F] Verticillium lamellicola

Ventiloquinone-E	8773	0.223	-33.775	3	C18 H20 O6	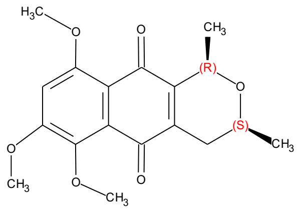	[Pl] Ventilago maderaspatana
Lambertellin	2074	0.223	-29.438	3	C14 H8 O5	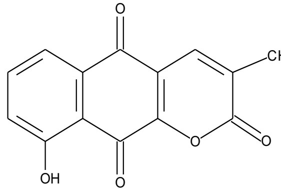	[F] Lambertella nicoriae, Lambertella carnimaris, Pseudospiropes simplex
8-Hydroxy-l,6-dimethyl-3-methylanthraquinone	16084	0.220	-50.794	3	C17 H14 O5	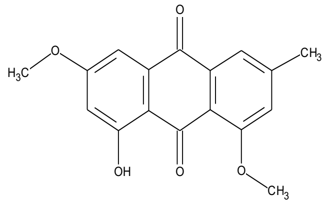	[F] Achaetomium cristalliferum
2,3-Diacetoxy-1,4-dihydroxyanthraquinone	20132	0.219	-56.791	3	C18 H12 O8	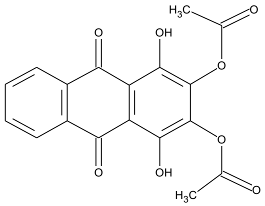	[synthetic]
6-Bromo-5-hydroxy-2-methylnaphthoquinone	20183	0.219	-19.797	2	C11 H7 Br1 O3	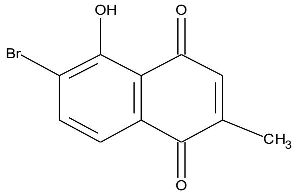	[synthetic]

Deoxynyboquinone	42043	0.217	-34.248	3	C15 H12 N2 O4	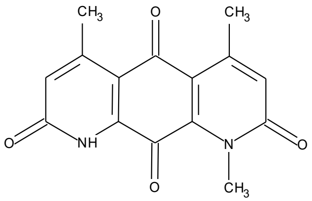	[B] deep-sea actinomycete Pseudonocardia sp. SCSIO 01299
Phenicin	2606	0.216	3.048	1	C14 H10 O6	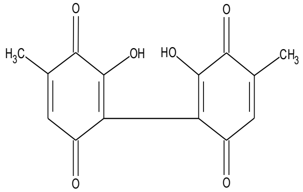	[F] Penicillium phoeniceum, P.rubrum, P.chermesinum
(1S,3S,4S)-Nanaomycin D	4552	0.216	-37.716	3	C16 H12 O6	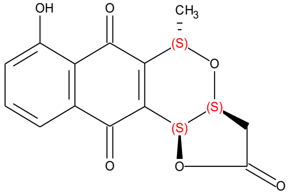	[B] Streptomyces rosa var. notoensis os-3966 (FERM-p 2209)
1-Bromo-4-hydroxy-5,7-dimethoxy-2-methylanthraquinone	20124	0.216	-30.845	3	C17 H13 Br1 O5	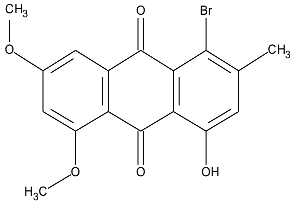	[synthetic]
Cryptosporin	11939	0.215	-27.626	3	C14 H12 O6	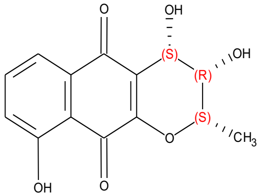	[F] Cryptosporium pinicola

6-Methylxanthopurpurin-3-methyl ether	10048	0.211	-46.296	3	C16 H12 O4	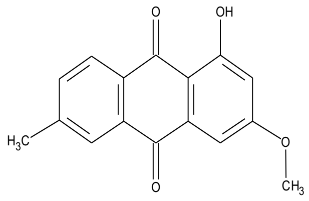	
Cinnaquinone	7210	0.210	-45.674	1	C7 H5 N1 O5	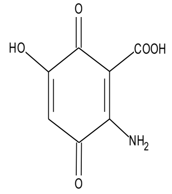	[B] Streptomyces griseoflavus ssp. thermodiastaticus Tue 2486
Sakyomicin D	4714	0.209	-40.523	4	C19 H18 O8	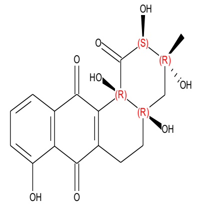	[B] Nocardia sp. 53, M53
SCH 38519	5708	0.209	-50.229	3	C24 H25 N1 O8	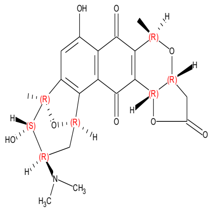	[B] Thermomonospora sp.
(-)-Kinamycin A	4444	0.209	-57.518	2	C24 H20 N2 O10	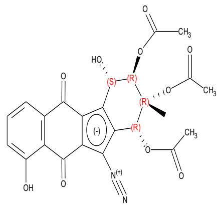	[B] Streptomyces murayamaensis

Sarubicin B	2874	0.206	-57.754	2	C13 H10 N2 O4	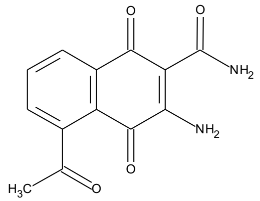	[B] Streptomyces violaceoruber ja 2861
Coronatoquinone	26075	0.204	-42.040	3	C15 H12 O8	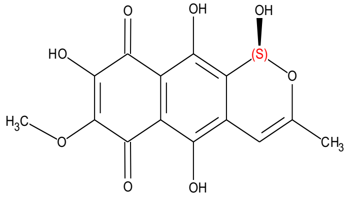	[L] lichen Pseudocyphellaria coronata
2-(1-Hydroxyethyl)naphthol[2,3-b]furan-4,9-quinone	15051	0.201	-31.152	2	C14 H10 O4	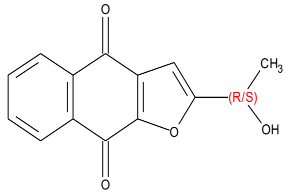	
F7	5386	0.199	-37.141	2	C15 H14 O5	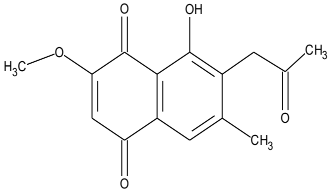	[F] Fungus, Fusarium sp.
8-Hydroxy-2,5,6-trimethoxy-3-(2-oxopropyl)-1,4- naphthoquinone	5044	0.199	-38.275	2	C16 H16 O7	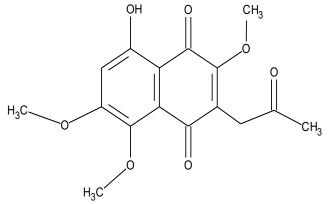	[F] Fusarium solani

Sakyomicin B	4712	0.199	-37.154	4	C19 H16 O8	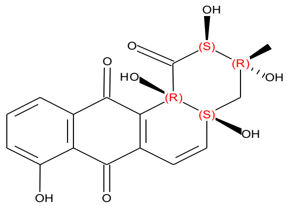	[B] Nocardia sp. 53, M53
5-Chloro-6,8-dihydroxy-1-methoxy-3-methylanthraquinone	14238	0.194	-44.613	3	C16 H11 Cl1 O5	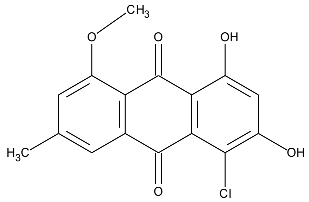	[L] [F] fungus Phialophora alba, lichen Nephroma laevigatum
a-Ethylfurano-1,4-naphthoquinone	10080	0.194	-24.412	3	C14 H10 O3	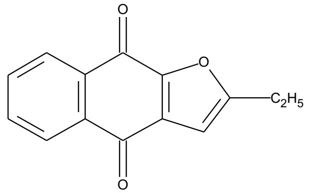	
Julichrome Q3,4	4431	0.189	-91.401	3	C38 H36 O16	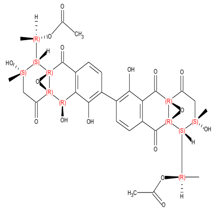	[B] Streptomyces shinodaensis
2-Isopropenylnaphtho[2,3-b]furan-4,9-quinone	15053	0.189	-22.950	2	C15 H10 O3	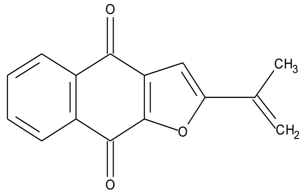	

1-Hydroxy-3-methoxy-6-methyl-anthraquinone 1-O-b-D-gentiobioside	11475	0.186	-72.155	3	C28 H32 O14	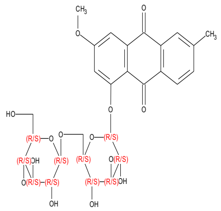	[F] fungus Dermocybe splendida Horak
Anthraquinone-2-aldehyde	10130	0.186	-24.163	3	C15 H8 O3	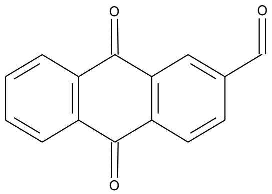	
Anthraquinone-2-carboxylic acid	10131	0.183	-39.239	3	C15 H8 O4	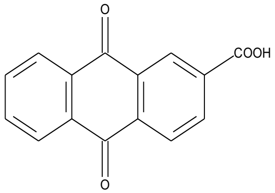	
Ventiloquinone H	15188	0.182	-30.097	3	C17 H18 O6	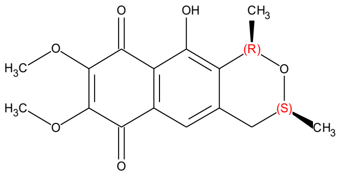	thomson
1,3,7-Trimethoxy-6-methylanthraquinone	3934	0.180	-43.747	3	C18 H16 O5	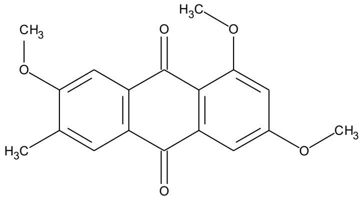	[F] Macrosporium porri

Ventiloquinone F	15187	0.179	-30.139	3	$C_{16} H_{16} O_{5}$	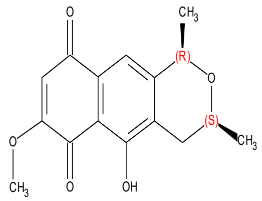	thomson	
Diazaquinomycin B	1418	0.178	-34.744	3	$C_{20} H_{24} N_{2} O_{4}$	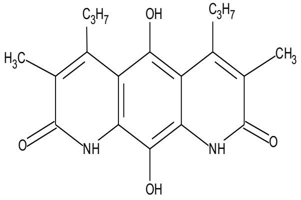	[B] Streptomyces sp. om-704	2
Fusarnaphthoquinone C	38691	0.176	-75.020	3	$C_{29} H_{26} O_{11}$	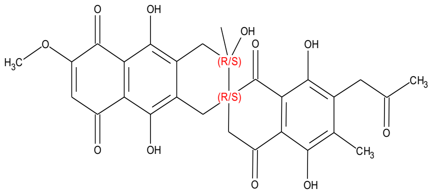	[F] Fusarium spp. PSU-F14 and PSU-F135	
Anhydrojavanicin	8086	0.176	-27.240	2	$C_{15} H_{12} O_{5}$	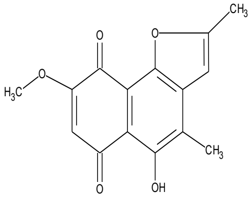	[F] Fusarium solani	
Mollisin	10586	0.172	-25.659	2	$C_{14} H_{10} Cl_{2} O_{4}$	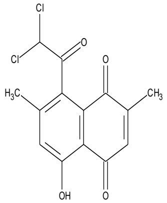	[F] Mollisia caesia, Mollisia fellens	

2,5 (or 3,5)-Dihydroxy-1,3,4 (or 1,2,4)-trimethoxyanthraquinone	15442	0.170	-35.419	3	C17 H14 O7	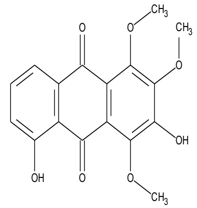	
O-Demethylanhydrofusarubin	13141	0.169	-51.070	3	C14 H10 O6	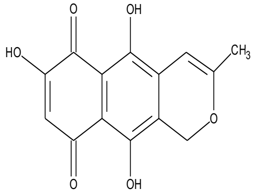	[F] Gibberella fujikuroi
Yoronomycin	3891	0.169	-32.260	4	C19 H16 O8	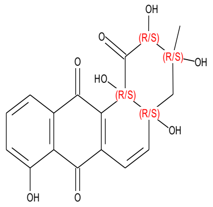	[B] Streptomyces phaeochromogenes var. yoronensis FERM-p 2893
Kwanzoquinone A monoacetate	39182	0.168	-34.463	3	C20 H16 O5	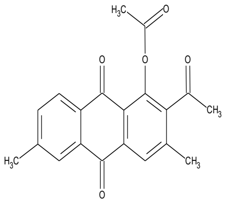	[Pl] Homerocallus fulva
Kwanzoquinone B monoacetate	39184	0.166	-35.810	3	C20 H16 O5	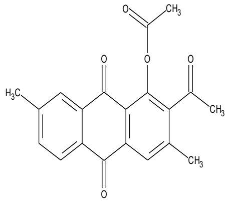	[Pl] Homerocallus fulva

Coniothranthraquinone	41086	0.165	-33.867	3	C15 H14 O5	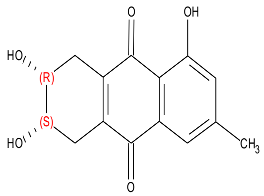	[F] marine-derived Trichoderma aureoviride PSU-F95
4-Deoxyanhydrofusarubin	6964	0.163	-29.965	3	C15 H12 O5	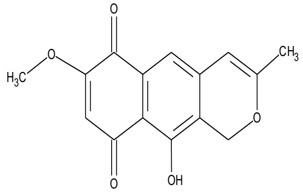	[F] Nectria haematococca
Anhydrofusarubin-lactol	5899	0.163	-47.706	3	C15 H12 O7	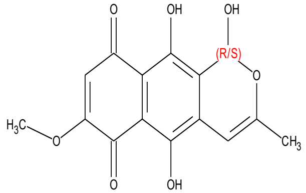	[F] Fusarium solani
4-Hydroxypiloquinone	9995	0.163	-31.935	3	C21 H20 O6	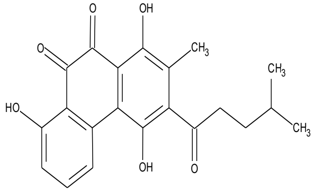	[B] Streptomyces pilosus
Antibiotic Zgg	3144	0.162	-54.005	4	C22 H16 O8	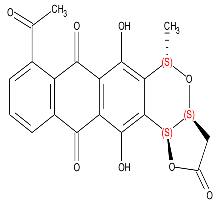	[B] Streptomyces thermoviolaceus subst. pigens var. wr-141

Desoxyfrenolicin	4228	0.161	-45.299	3	C18 H18 O6	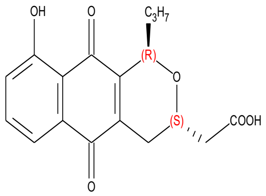	[B] Streptomyces roseofulvus am-3867 (FERM-p 4359), S.fradiae, S.roseoflavus
9-Hydroxy-1,4-anthraquinone	20232	0.161	-20.813	3	C14 H8 O3	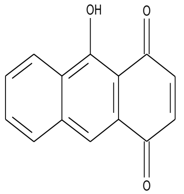	[synthetic]
2-Hydroxy-6-methoxy-3,5-dimethyl-1,4-benzoquinone	15750	0.160	-35.398	2	C13 H12 O4	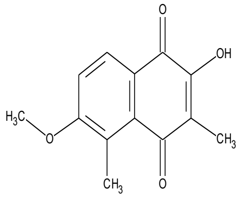	[F] Phoma wasabiae
(3S,3'S,P)-Anhydrophlegmacin-9,10-quinone 8'-O-methyl ether	16219	0.156	-72.547	3	C33 H28 O10	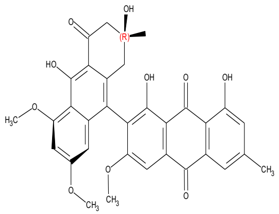	[F] Cortinarius percomis, C. sinapicolor Cleland
Fumaquinone	32235	0.155	-33.069	2	C17 H18 O5	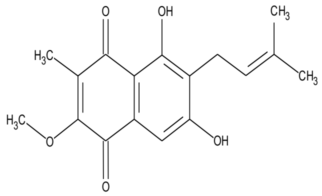	[B] Streptomyces fumanus LL-F42248

Altersolanol B	254	0.152	-36.227	3	C16 H16 O6	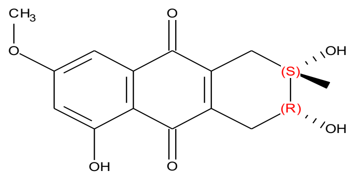	[F] Alternaria porri; Dichotomophthora lutea, Dactylaria lutea, Alt.solani
Anhydrofusarubin	5184	0.151	-45.477	3	C15 H12 O6	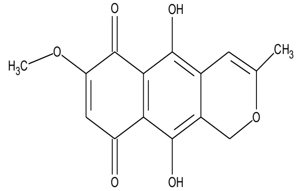	[F] (fungus) Fusarium decemcellulare, F. solani
Trichodermaquinone	41084	0.151	-44.280	3	C15 H14 O6	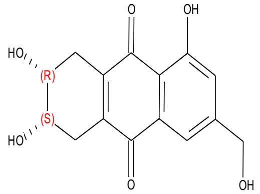	[F] marine-derived Trichoderma aureoviride PSU-F95
Nanaomycin-betaA	4553	0.149	-30.325	3	C16 H16 O5	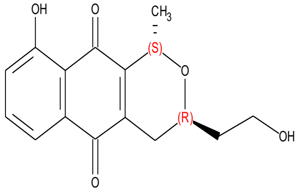	[B] Streptomyces sp. om-173 (FERM-p 6509), Streptomyces roseofulvus
(+)-Mycorrhizin A	1951	0.148	-26.347	1	C14 H15 Cl1 O4	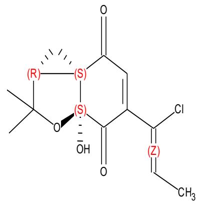	[F] (ascomycete) Lachnum papyraceum + CaBr2, Cochliobolus lunatus, [F] "ectenoomycorrhizal fungus", Monotropa hypopitys, Gilmaniella humicola

Murayaanthraquinone	15275	0.148	-52.900	3	C26 H16 N2 O7	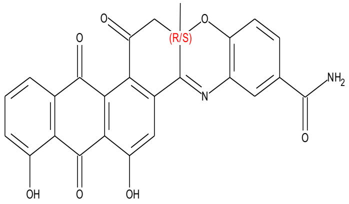	[B] Streptomyces murayamaensis mutant
Anhydrofusarubin lactone	6154	0.148	-57.641	3	C15 H10 O7	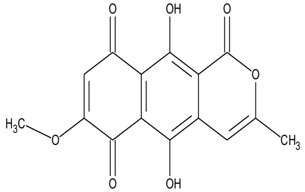	[F] Fusarium solani, Nectria haematococca
7-Hydroxy-1,8-dimethoxy-2,3-methylene-dioxyanthraquinone	1185	0.147	-39.882	3	C17 H12 O7	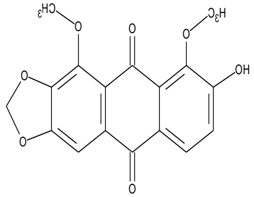	[An] Tubastraea micrantha
A-7884	8812	0.147	-93.141	4	C37 H42 O14	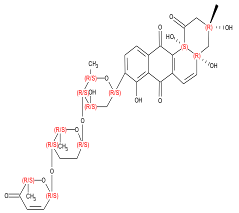	[B] Streptomyces griseovirides, marine [B] S. sp. B6728
Nanaomycin C	4551	0.147	-42.035	3	C16 H15 N1 O5	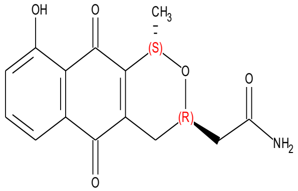	[B] Streptomyces rosa var. notoensis os-3966 (FERM-p 2209)

(-)-Phyllostine	7613	0.144	-28.695	1	C7 H6 O4	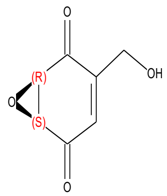	[F] endophytic Cryptosporiopsis sp. CAFT122-1 [F] Penicillium megasporum nhl 2977, NRRL 2232, ATCC 48997, Phyllosticta sp., [B] P.urticae, Ophiosphaerella herpotricha
4,11-Dihydroxy-5-methoxy-2,9-dimethyldinaphtho[1,2-b:2',3'-d]furan-7,12-quinone	15068	0.144	-30.343	4	C23 H16 O6	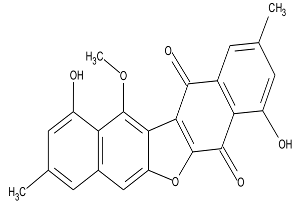	
3,4,6,9-Tetrahydro-10-hydroxy-7-methoxy-3-methyl-1,6,9-trioxo-1H-naphtho-[2,3-c]pyran	9933	0.144	-49.006	3	C15 H12 O6	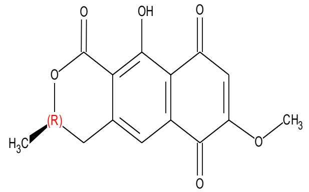	[F] Penicillium citreo-viride
Norbikaverin	13119	0.139	-62.638	4	C19 H12 O8	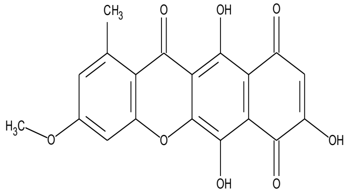	[F] Gibberella fujikuroi, Fusarium solani-Pisi
Ventiloquinone C	15193	0.137	-42.592	3	C16 H16 O7	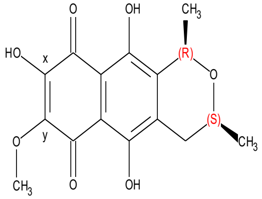	thomson

3,5,7-Trihydroxy-2-methoxy-1,4-naphthoquinone	8947	0.136	-12.146	2	C11 H8 O6	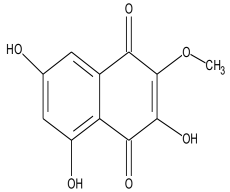	[F] Cercospora melonis	
Ventiloquinone D	15192	0.136	-42.321	3	C17 H18 O7	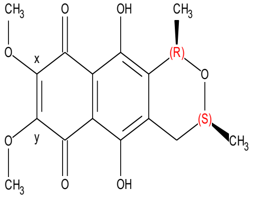	thomson	
Iso-aeroplysinin-1	9804	0.136	-30.806	1	C9 H9 Br2 N1 O3	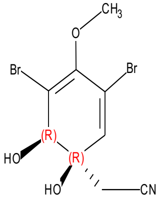	[An] Aplysia aerophorba, Verongia archeri, Ianthella sp., Sponge	2
1,2-Methylenedioxyanthraquinone	1964	0.135	-28.949	3	C15 H8 O4	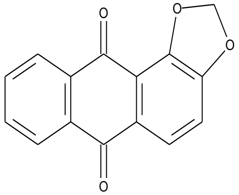	[Pl] Morinda parviflora	
2-Hydroxy-1,6-dimethoxy-3-methylanthraquinone	9906	0.135	-30.388	3	C17 H14 O5	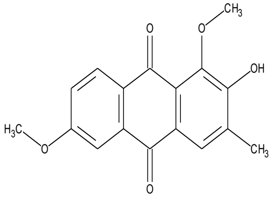		

Anhydrophlegmacinquinone B2	17205	0.135	-57.943	3	C32 H26 O10	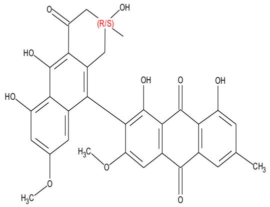	[Pl] Cassia torosa, Senna multiglandulosa; [F] Cortinarius sp.
Anhydrophlegmacinquinone A2	15336	0.135	-56.097	3	C32 H26 O10	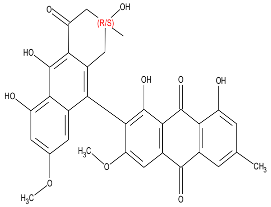	[Pl] Cassia torosa, Senna multiglandulosa [F] Cortinarius odorifer
Phosphatoquinone-B	23210	0.131	-31.950	2	C21 H24 O4	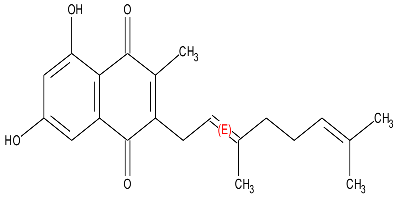	[B] Streptomyces sp. TA-0363
Brasiliquinone-B	20595	0.130	-32.197	4	C20 H16 O5	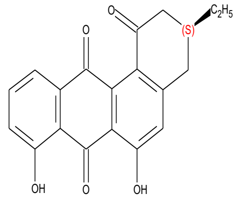	[B] Nocardia brasiliensis
Lepiotaquinone	26202	0.129	-37.115	1	C12 H10 N2 O3	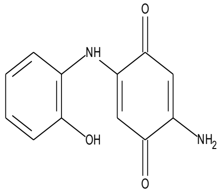	[F] Lepiota americana (Agaricales)

Saframycin S	2854	0.128	-58.597	4	C28 H31 N3 O9	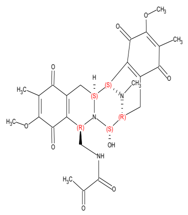	[B] Streptomyces lavendulae 314 (FERM-p 3218, NRRL 11002)
1,3,6,8-Tetrahydroxy-2-(1'-hydroxy-3'-oxobutyl)-anthraquinone	21341	0.126	-71.898	3	C18 H14 O8	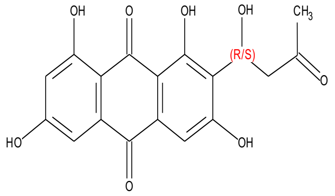	[F] Aspergillus parasiticus
MM-061	28702	0.123	-55.630	3	C20 H18 O9	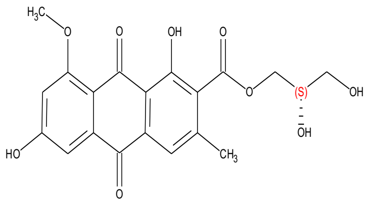	[F] marine fungi [F] Aspergillus variecolor B-17 [F] marine Aspergillus glaucus
Ventiloquinone-O	23288	0.123	-42.854	3	C16 H14 O7	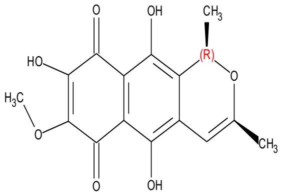	[Pl] Ventilago goughii
Panepoxydione	10668	0.121	-28.261	1	C11 H12 O4	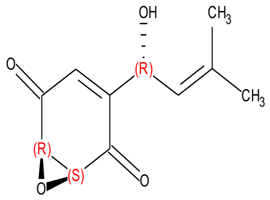	[F] Panus rudis, Penicillium conchatus

F2	5385	0.119	-39.748	2	C15 H14 O5	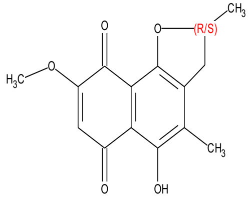	[F] Fusarium solani; "Munissi MUF2"	
(+)-Aeroplysinin-1	9790	0.119	-21.204	1	C9 H9 Br2 N1 O3	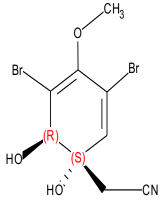	[An] Aplysia aerophorba, Verongia archeri, Ianthella sp., Sponge	2
Dianhydroflavomannin-9,10-quinone-6,6'-di-O-methyl ether	17557	0.118	-51.109	3	C32 H24 O9	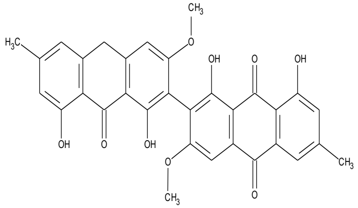		
9-Chloro-10-hydroxy-1,4-anthraquinone	20138	0.118	-21.868	3	C14 H7 Cl1 O3	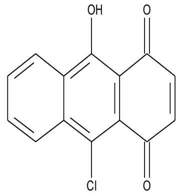	[synthetic]	
(-)-Kinamycin C	4445	0.118	-66.818	2	C24 H20 N2 O10	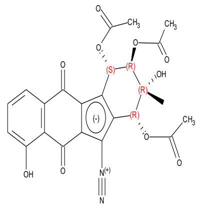	[B] Streptomyces murayamaensis	

Bikaverin	521	0.117	-56.678	4	C20 H14 O8	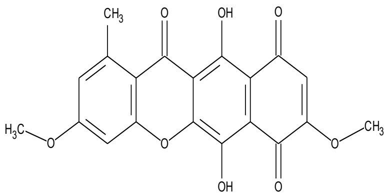	[F] Gibberella fujikuroi, Fusarium moniliforme, Fus.oxysporum, Mycogone jaapii, Fus.lycopersicii, Fus.vasinfectum, Fus.solanipisi
1-Hydroxy-8-methoxyanthraquinone	5906	0.116	-27.823	3	C15 H10 O4	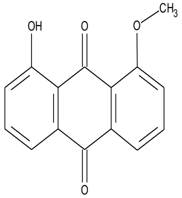	[F] Leptographium wageneri
5-Deoxyfusarubin	8994	0.116	-31.262	3	C15 H14 O6	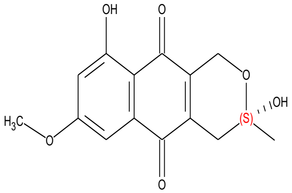	[F] (fungus) Nectria haematococca
Rubellin A	2838	0.115	-74.723	3	C30 H22 O9	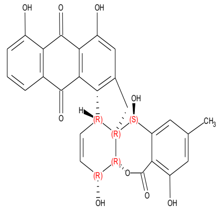	[F] Mycosphaerella rubella
2,9-Epoxydeliquinone	22550	0.113	-36.319	1	C14 H18 O5	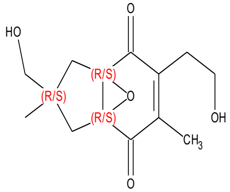	[F] Russula delica

1-Hydroxy-5-methoxy-anthraquinone	20121	0.113	-28.296	3	C15 H10 O4	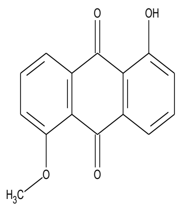	[synthetic]
5-Geranyl-3,6-dihydroxy-2-methyl-1,4-naphthoquinone	15102	0.112	-38.000	2	C21 H24 O4	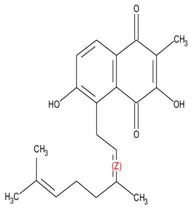	
(-)-Kinamycin D	4446	0.112	-60.220	2	C22 H18 N2 O9	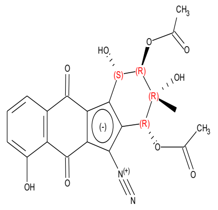	[B] Streptomyces murayamaensis
Anthraquinone	17209	0.112	-19.770	3	C14 H8 O2	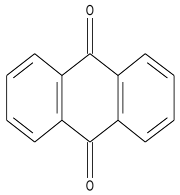	
2,5,7-Trihydroxy-1,4-naphthoquinone	3987	0.109	-42.043	2	C10 H6 O5	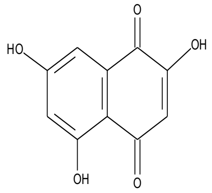	[F] Aspergillus niger; Verticillium dahliae; Phialophora lagerbergii

1,3-Dihydroxy-8-methoxy-6-methoxymethylanthraquinone	3936	0.109	-48.061	3	C17 H14 O6	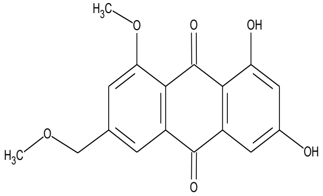	[Pl] Melanoxylon brauna
Gonioquinone	38447	0.106	-40.717	2	C12 H8 O6	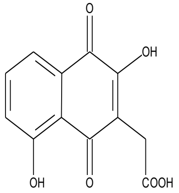	[Pl] Goniothalamus cheliensis Hu
Coleone-U-quinone	17190	0.104	-27.831	3	C20 H24 O6	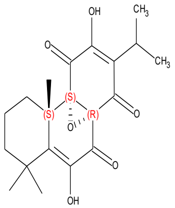	
8-Hydroxy-2-isopropenylnaphtho[2,3-b]furan-4,9-quinone	15094	0.104	-29.073	2	C14 H8 O5	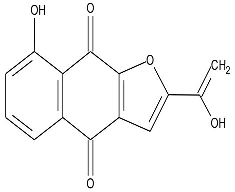	
Tectoquinone	10813	0.104	-18.311	3	C15 H10 O2	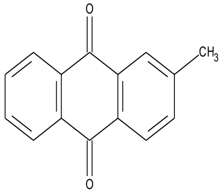	roots of Rubia cordifolia, Z R. tinctorum, 3, 7 R. oncotricha,18 Prismatomeris tetrandra4 and Morinda ojficinalis5 (all Rubiaceae), and in the fern Lygodium flexuosum6.- thomson

2,5,7-Trihydroxy-3-methoxy-1,4-naphthoquinone	8919	0.102	-14.882	2	C11 H8 O6	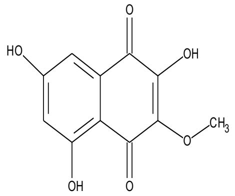	[F] Cercospora melonis
4-Hydroxy-3-hydroxymethyl-5,6-dimethoxyanthraquinone	15326	0.102	-36.542	3	C17 H14 O6	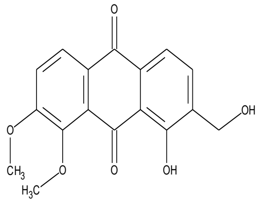	
2-Hydroxyanthraquinone-3-aldehyde	15287	0.101	-25.095	3	C15 H8 O4	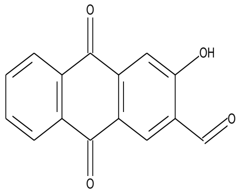	
Ventiloquinone-L	23286	0.100	-24.982	3	C16 H16 O5	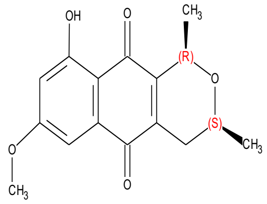	[Pl] Ventilago goughii
Helicquinone	22225	0.097	-24.444	2	C15 H14 O4	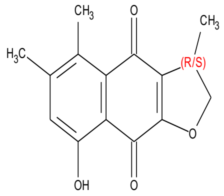	[Pl] Helicteras angustifolia

2-Hydroxymethylanthraquinone	9913	0.096	-29.506	3	C15 H10 O3	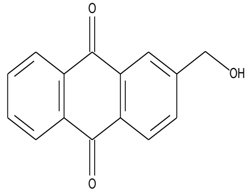	[F] rhizomes of Curcuma domestica (Zingiberaceae). Se thomson
6-O-Demethyl-5-deoxyfusarubin	7023	0.096	-37.635	3	C14 H12 O6	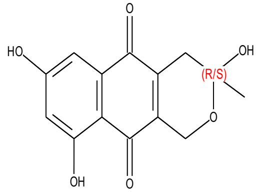	[F] Nectria haematococca
7,7'-Bi(3-ethyl-2,6-dihydroxynaphthazarin)	15149	0.091	-49.489	2	C24 H18 O12	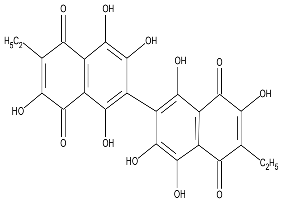	
Anhydroflavomannin-5,8-quinone-6,6'-di-O-methyl ether	17504	0.089	-58.844	3	C32 H26 O10	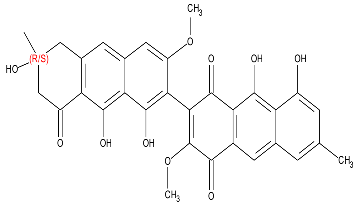	
6-O-Demethyl-5-deoxyanhydrofusarubin	7021	0.087	-31.517	3	C14 H10 O5	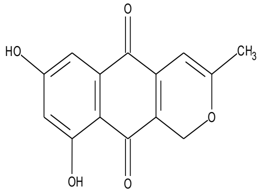	[F] Nectria haematococca; ascomycete Trichopezizella nidulus

Vismiaquinone B	15366	0.086	-30.391	3	C21 H20 O6	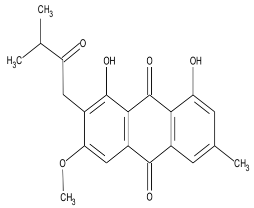	thomson
5,10-Dihydroxy-1,4-anthraquinone	20237	0.081	-21.816	3	C14 H8 O4	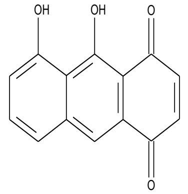	[synthetic]
5-Hydroxy-2,7-dimethoxy-1,4-naphthoquinone	4072	0.081	-30.803	2	C12 H10 O5	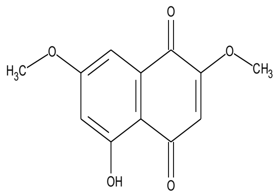	[B] Actinomyces sp. no. 12396
Sinapiquinone	24040	0.080	-81.300	3	C33 H24 O10	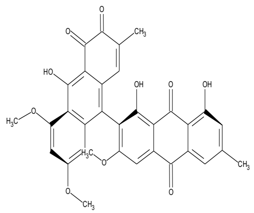	[F] Cortinarius sinapicolor
10-Hydroxy-5-methoxy-2-methyl-1,4-anthraquinone	20236	0.079	-24.599	3	C16 H12 O4	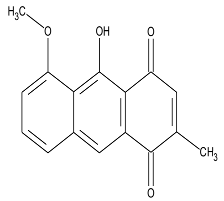	[synthetic]

2-Hydroxyanthraquinone	9912	0.077	-28.766	3	C14 H8 O3	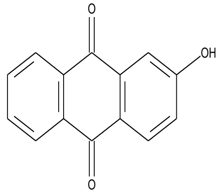	
4-Deoxyfusarubin	6965	0.076	-37.073	3	C15 H14 O6	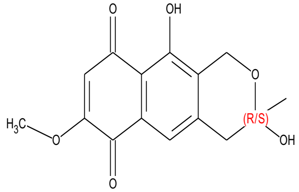	[F] Nectria haematococca; "Munissi MUF2"
4,4'-Bis(1,3,8-trihydroxy-3-methyl-6-methoxyanthraquinone)	22500	0.075	70.840	3	C32 H22 O12	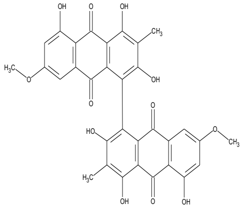	[Pl] Cassia hirsuta
Bisnorbadioquinone-A	15230	0.073	######	2	C34 H18 O14	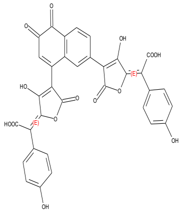	
3-O,9-O-Dimethylfusarubin	7957	0.073	-36.075	3	C17 H18 O7	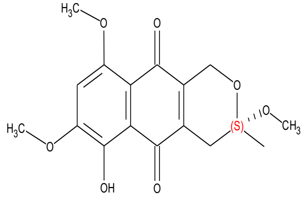	[F] Fusarium oxysporum; semisynth.

Ledgerquinone	15376	0.072	-37.826	3	C17 H12 O6	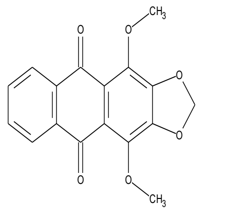	
5-Deoxy-3,4-anhydrofusarubin	8993	0.072	-26.292	3	C15 H12 O5	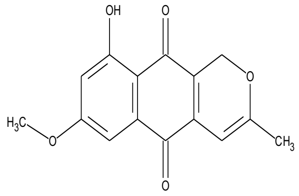	[F] (fungus) Nectria haematococca; ascomycete Trichopezizella nidulus
3,8-Dihydroxy-1-methoxy-9,10-anthraquinone	4981	0.070	-40.959	3	C15 H10 O5	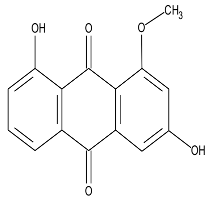	[B] Xenorhabdus luminescens (entomopathogenic bact.)
Naphthoquinone 4	22942	0.070	-73.156	3	26 H32 N2 O9 S1	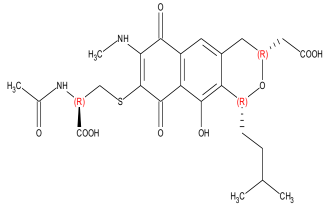	[B] Streptomyces sp.
1,3-Dimethoxy-8-hydroxy-9,10-anthraquinone	19155	0.070	-36.288	3	C16 H12 O5	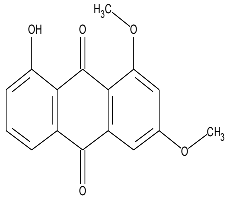	[B] entomopathogenic bacterium Photorhabdus luminescens

Versicolorin A	4850	0.069	-52.963	3	C18 H10 O7	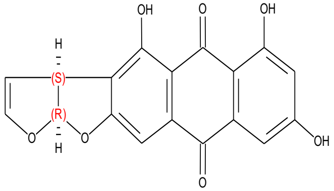	[F] Aspergillus versicolor, A. parasiticus
(-)-Kinamycin B	2062	0.068	-44.374	2	C20 H16 N2 O8	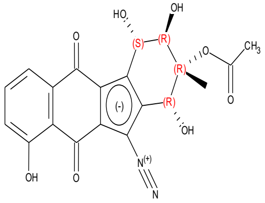	[B] Streptomyces murayamaensis
3-Hydroxy-2-methylanthraquinone	9961	0.067	-31.601	3	C15 H10 O3	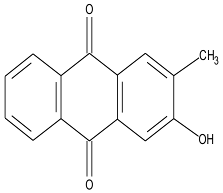	
(2'S)-Hydroxy-(3S,3'S,P)-anhydrophlegmacin-9,10-quinone 8'-O-methyl ether	23635	0.067	-39.404	3	C33 H28 O11	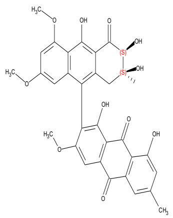	[F] Cortinarius sinapicolor Cleland
6-(1-Hydroxyethyl)-2,7-dimethoxyjuglone	8001	0.066	-38.743	2	C14 H14 O6	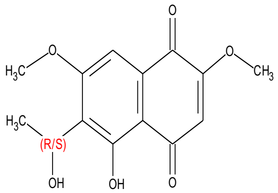	[F] Kirschsteiniothelia sp.; Guignardia laricina gm-7, Hendersonula toruloidea

2-Methoxy-3-methylanthraquinone	17208	0.064	-24.612	3	C16 H12 O3	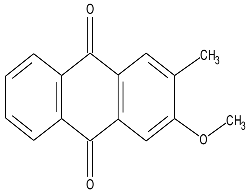	[Pl] Coprosma tenuicauli, C. linariifolia (Rubiaceae)
g-Actinorhodin	9308	0.064	-72.914	3	C32 H22 O14	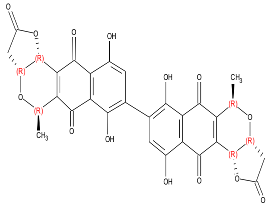	[B] Streptomyces coelicolor
Haloquinone	6414	0.064	-30.246	3	C17 H12 O5	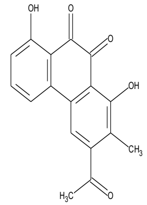	[B] Streptomyces venezuelae subsp. xanthophaeus Tue 2115
Questin	10729	0.064	-41.132	3	C16 H12 O5	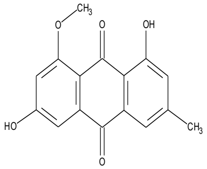	[F] Penicillium frequentans
Methyl 3,4,8-trihydroxy-1-methylanthraquinone-2-carboxylate 4-methyl ether	15343	0.063	-30.493	3	C18 H14 O7	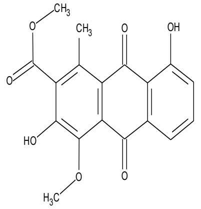	

2-(2,3-Dihydro-5-methyl-6-oxopyran-2-yl)-5,8-dihydroxy-1,4-naphthoquinone	15123	0.063	-36.658	2	C16 H12 O6	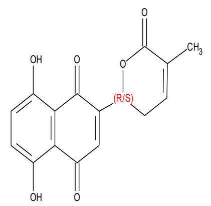	
2-Methoxy-anthraquinone	20249	0.063	-26.177	3	C15 H10 O3	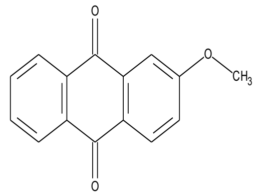	[synthetic]
anthraquinones 9	41622	0.063	-74.077	4	C27 H28 O13	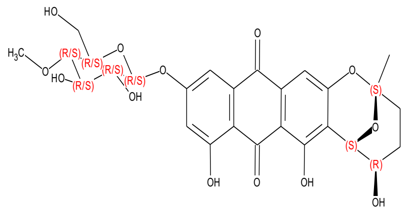	[F] scale insect fungus Aschersonia coffeae Henn. BCC 28712
6-(1-Ethoxyethyl)-5-hydroxy-2,7-dimethoxy-1,4-naphthoquinone	6070	0.062	-34.200	2	C16 H18 O6	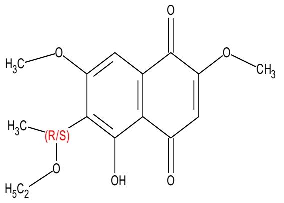	[F] Guignardia laricina gm-7
Ventiloquinone A	15195	0.061	-31.215	3	C17 H16 O7	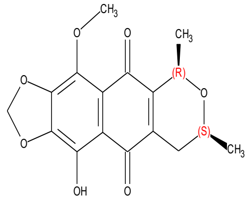	thomson

7,10-Dihydroxy-5-methoxy-2-methyl-1,4-anthraquinone	4088	0.060	-34.663	3	C16 H12 O5	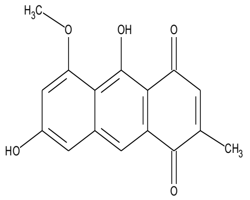	[F] Aspergillus ruber ifo 6004; Asp. cristatus (Eurotium cristatum)
5,6-Dihydroxy-2-methylnaphthoquinone	20180	0.060	-20.477	2	C11 H8 O4	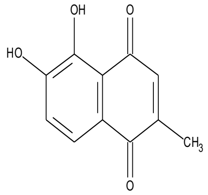	[synthetic]
2-Amino-3,6-dioxo-4,5-epoxycyclohexane-carboxylic acid	1048	0.060	-51.390	1	C7 H5 N1 O5	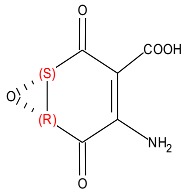	[B] Streptomyces baarnensis 13120 (FERM-p 3938) S. fulvoviolaceus 851 (FERM-p 4223)
Anhydroflavomannin-9,10-quinone 6,6'-dimethyl ether	15334	0.059	-66.185	3	C32 H26 O10	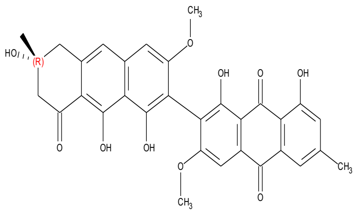	[F] Dermocybe cinnamomeolutea
Eleuthraquinone B	40368	0.059	-44.175	3	C18 H14 O8	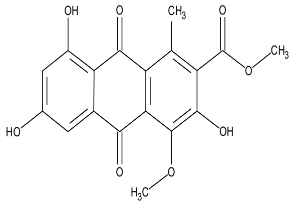	[Pl] Eleutherine americana

Draculone	29307	0.058	-42.067	3	C16 H10 O8	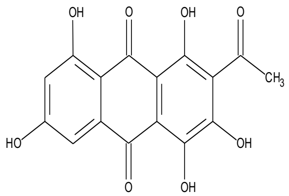	[L] lichen Melanotheca cruenta (= Trypethelium cruentum = Pyrenula cruenta)
8-Hydroxy-6-hydroxymethyl-1,3-dimethoxyanthraquinone	4106	0.058	-47.567	3	C17 H14 O6	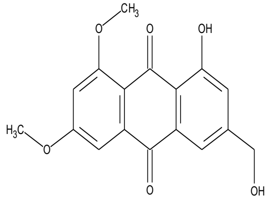	[Pl] Melanoxylon brauna
Norjavanicin	2429	0.058	-29.595	2	C14 H12 O6	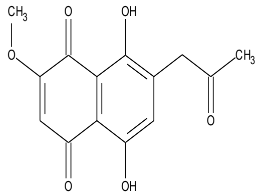	[F] Fusarium decemcellulare, Fus.martici-pisi, Neocosmospora vasinfecta, Fusarium martiela, Fusarium solani, Necosmospora africana, Necosmospora haematococca
SF-2418	5725	0.057	-29.401	3	C19 H16 O5	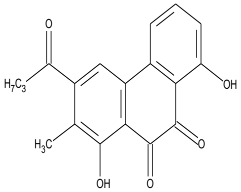	[B] Streptomyces sp. sf-2418; S. murayamaensis
Y 1005	6841	0.057	-38.601	2	C16 H16 O7	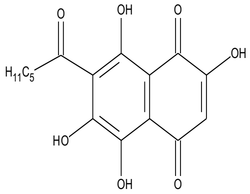	[Y] fusiform yeast strain by1

Piloquinone	10689	0.057	-30.254	3	C21 H20 O5	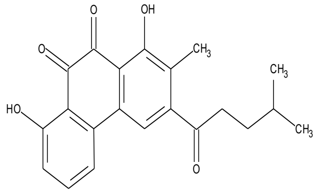	[B] Streptomyces pilosus
6-Ethyl-5,7-dihydroxy-2-methoxy-naphthoquinone	8004	0.056	-18.219	2	C13 H12 O5	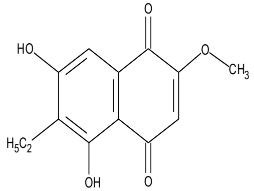	[F] (fungus) Kirschsteiniothelia sp.
6-Ethyl-2,7-dimethoxyjuglone	8003	0.053	-29.023	2	C14 H14 O5	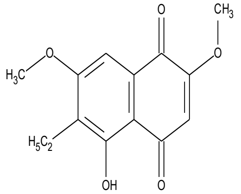	[F] (fungus) Kirschsteiniothelia sp., Hendersonula toruloidea [F] Phaeosphaeria sp. BCC8292 [F] Perenniporia sp. from larva of Euops chinesis
(±)-Versicolorin C	3932	0.051	-55.675	3	C18 H12 O7	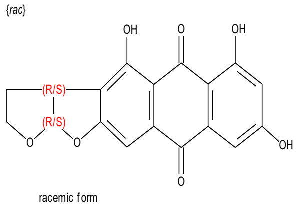	[F] Aspergillus versicolor
5-Hydroxy-1,3-dimethoxy-7-methylanthraquinone	42315	0.051	-37.406	3	C17 H14 O5	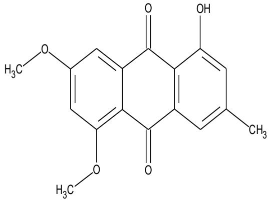	[F] endophytic Aspergillus wentii from red alga

4-Deoxyjavanicin	6966	0.051	-38.934	2	C15 H14 O5	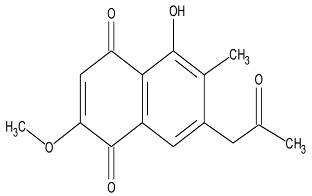	[F] Nectria haematococca
1,5',8'-Trihydroxy-3,3'-dimethyl-2,2'-binaphthyl-5,8,1',4'-diquinone	20176	0.051	-33.071	2	C22 H14 O7	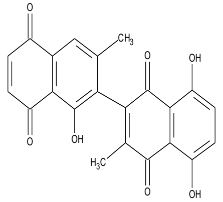	[synthetic]
3-Bromo-4-hydroxy-5,10-dioxo-1-antrha-cenecarboxylic acid	91	0.050	-12.416	3	C15H7Br1O5	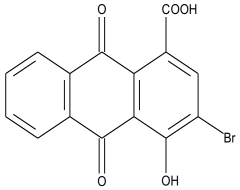	[x]Tubeastraea micrantha
Versicolorin-B	4851	0.050	-53.758	3	C18 H12 O7	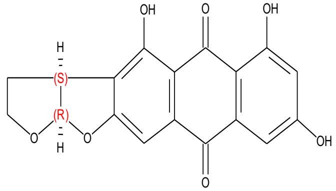	[F] Aspergillus versicolor
1-Hydroxy-6,8-dimothoxy-3-methylanthraquinone	15876	0.050	-37.110	3	C17 H14 O5	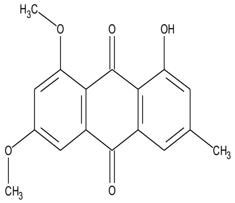	[F] A. cristalliferum, Aspergillus wentii

1-Hydroxy-6,8-dimethoxy-3-methylanthraquinone	20122	0.050	-37.112	3	$C_{17}H_{14}O_{5}$	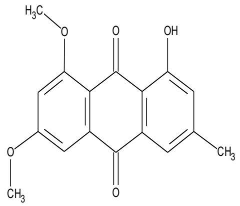	[synthetic]
5,5',8,8'-Tetrahydroxy-7,7'dimethyl-2,2'-binaphthoquinone	20161	0.049	-32.470	2	$C_{22}H_{14}O_{8}$	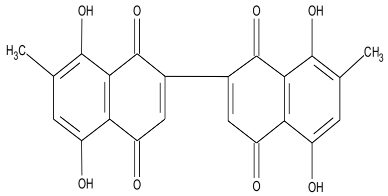	[synthetic]
1,3-Dihydroxy-5,7,8-trimethoxy-2-methylanthraquinone	15440	0.048	-38.979	3	$C_{18}H_{16}O_{7}$	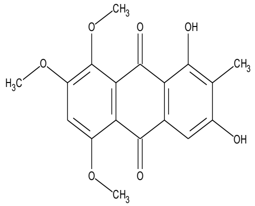	
Basidifferquinone-C	9116	0.047	-55.097	3	$C_{23}H_{14}O_{7}$	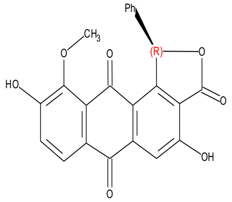	[B] Streptomyces sp. b-412
(S)-2,5,7-Trihydroxy-3-(5'-hydroxyhexyl)-1,4-naphthoquinone	4910	0.047	-46.782	2	$C_{16}H_{18}O_{6}$	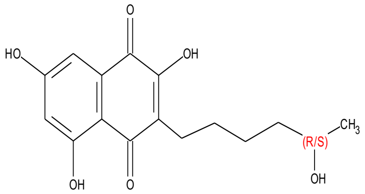	[F] Penicillium sp. 511

Pradimicin O	8585	0.047	-81.589	4	C29 H25 N1 O11	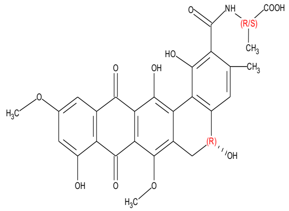	[B] blocked mutant of Actinomadura hibisca p157-2
Ventiloquinone-M	23287	0.045	-39.664	3	C16 H14 O7	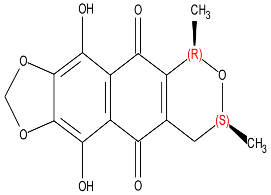	[Pl] Ventilago goughii
5,10-Dihydroxy-7-methoxy-1,4-anthraquinone	20230	0.044	-25.838	3	C15 H10 O5	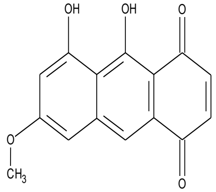	[synthetic]
1-Hydroxy-5,7-dimethoxy-2-methylanthraquinone	20157	0.043	-35.085	3	C17 H14 O5	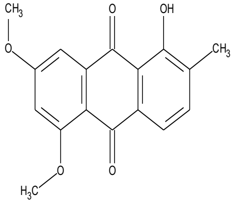	[synthetic]
1-Hydroxyanthraquinone-3-caboxylic acid	15285	0.042	-38.333	3	C15 H8 O5	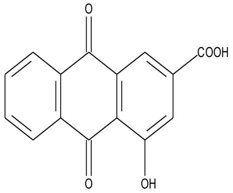	

Methyl 3,4,8-trihydroxy-1-methylanthraquinone-2-carboxylate 3,4-dimethyl ether	15344	0.040	-32.410	3	C19 H16 O7	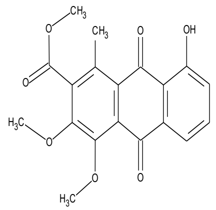	
10-Hydroxy-2,5-dimethoxy-7-methyl-1,4-anthraquinone	20228	0.040	-37.902	3	C17 H14 O5	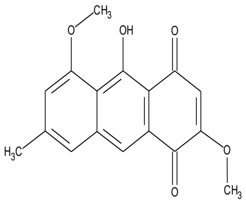	[synthetic]
Javanicunine B	33858	0.036	-39.900	1	C24 H30 N2 O5	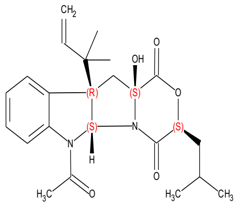	[F] Eupenicillium javanicum
SF 2446A1	5718	0.034	-80.909	4	C34 H35 N1 O15	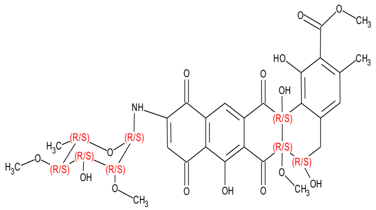	[B] Streptomyces sp. sf2446
1-Chloro-4-hydroxy-2-methylanthraquinone	20158	0.033	-21.196	3	C15 H9 Cl1 O3	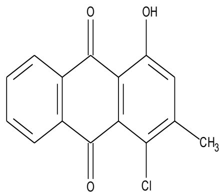	[synthetic]

Cordeaxione	10258	0.032	-33.568	2	C14 H12 O7	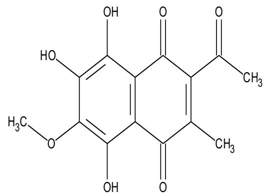	
1-Chloro-4-hydroxy-3-methylanthraquinone	20148	0.028	-22.682	3	C15 H9 Cl1 O3	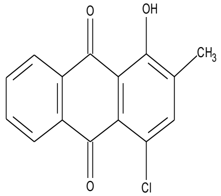	[synthetic]
Benzanthrin B	3200	0.027	-34.219	4	C35 H42 N2 O9	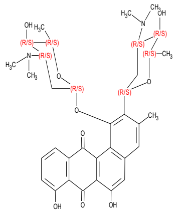	[B] Nocardia lurida
3-Hydroxy-8-O-acetyl-1-methylanthraquinone 2-carboxylic acid	31972	0.027	-45.138	3	C18 H12 O7	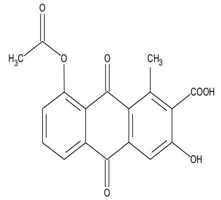	[synthetic] derivative
9-O-Methylfusarubin	8022	0.025	-37.033	3	C16 H16 O7	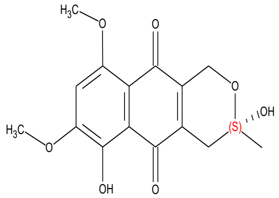	[F] Fusarium acutatum; Chickpea wilt; Fusarium oxysporum

trans-4'-Hydroxyanhydroflavomannin-9,10-quinone-6,6'-di-O-methyl ether	17611	0.024	-51.182	3	C32 H26 O11	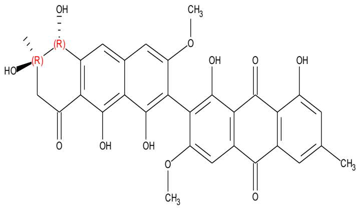	
Biruloquinone	8141	0.022	-36.227	4	C17 H10 O7	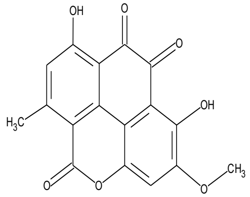	[L] Mycosphaerella rubella; Parmelia birulae
Komodoquinone A	30421	0.022	-76.036	4	C27 H31 N1 O10	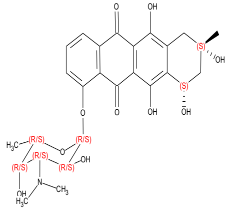	[B] marine Streptomyces sp. KS3
F9	5388	0.021	-28.078	2	C14 H12 O5	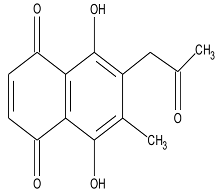	[F] Fungus, Fusarium sp.
3,5,8-Trihydroxy-6-methoxy-2-(5-oxohexa-1,3-dienyl)-1,4-naphthoquinone	23243	0.020	-39.302	2	C17 H14 O7	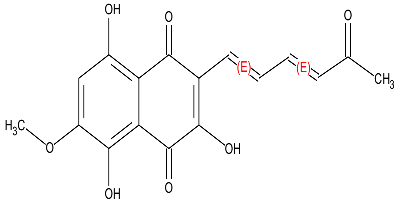	[F] insect pathogenic fungus Cordyceps unilateralis BCC1869

Rubellin C	6710	0.019	-60.414	3	C30 H22 O9	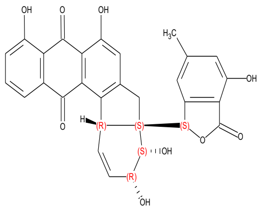	[F] Mycosphaerella rubella
3-Hydroxy-8-methoxy-1-propylanthraquinone	37933	0.019	-34.164	3	C18 H16 O4	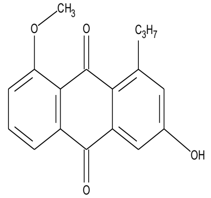	[B] Micromonospora rhodorangea
1-Hydroxy-4,6,8-trimethoxyanthraquinone	20259	0.018	-37.383	3	C17 H14 O6	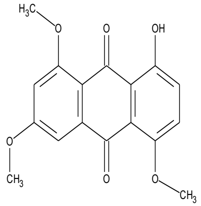	[synth.]
5,10-Dihydroxy-2-methyl-1,4-anthraquinone	20231	0.015	-20.653	3	C15 H10 O4	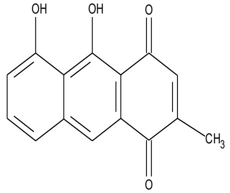	[synthetic]
5,8,8'-Trihydroxy-3,3'-dimethyl-2,2'-binaphthoquinone	20164	0.014	-32.481	2	C22 H14 O7	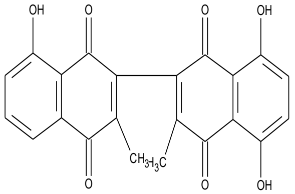	[synthetic]

Terremutin	13884	0.013	-31.746	1	C7 H8 O4	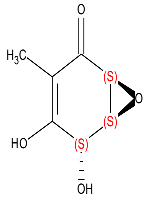	[F] Aspergillus terreus, Asp.paruvulus	1
6-Hydroxy-1,3-dimethoxy-7-methylanthraquinone	42013	0.012	-40.124	3	C17 H14 O5	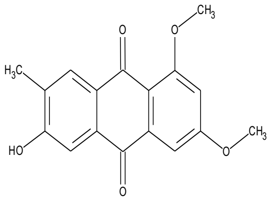	[F] freshwater fungus, Astrosphaeriella papuana YMF 1.01181	
b-Actinorhodin	6164	0.011	-72.757	3	C35 H28 O14	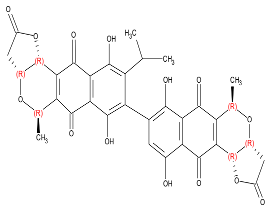	[B] Streptomyces coelicolor	
1,1',4,4'-Tetrahydroxy-6,6'-dimethyl-2,2'-binaphthyl-5,8	20185	0.010	-33.392	2	C22 H14 O8	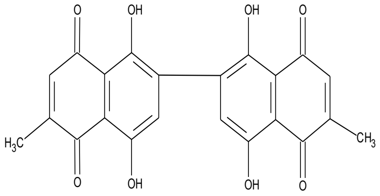	[synthetic]	
O-Demethyljavanicin	23217	0.010	-41.618	2	C14 H12 O6	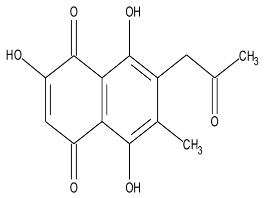	[F] Fusarium decemcellulare	

Galvaquinone C	41673	0.009	-51.989	3	C20 H18 O6	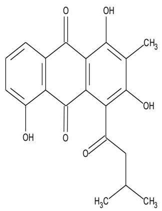	[B] marine-derived Streptomyces spinoverrucosus
Anyhdrofusarubin 9-methyl ether	15181	0.009	-31.761	3	C16 H14 O6	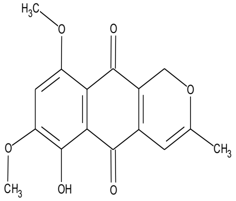	[F]fusarium - thomson
(1R,1'R,3S,3'S)-Actinorhodin	4898	0.008	-83.335	3	C32 H26 O14	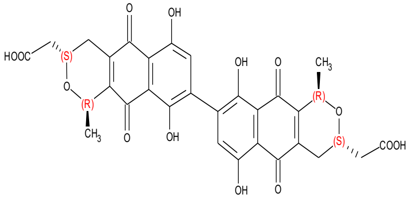	[B] Streptomyces coelicolor
Julichrome Q4,5	4432	0.007	-69.404	3	C36 H28 O13	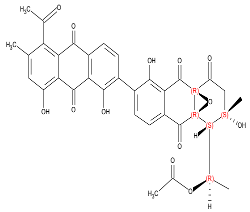	
Naphthoquinone 3	22941	0.004	-70.984	3	26 H32 N2 O9 S1	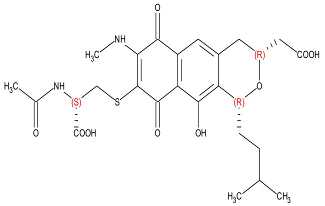	[B] Streptomyces sp.

2,3,5-Trihydroxy-1,4-naphthoquinone	17812	0.002	4.096	2	C10 H6 O5	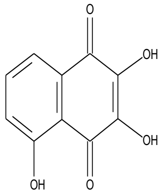	[S] Trichia floriformis
1-Hydroxyanthraquinone-2-carboxylic acid methyl ester	20136	0.001	-29.532	3	C16 H10 O5	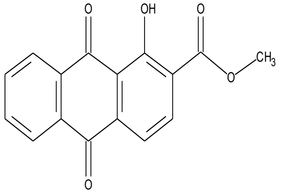	[synthetic]
1,5-Dihydroxy-2,6-dimethoxy-anthraquinone	20118	-0.001	-38.674	3	C16 H12 O6	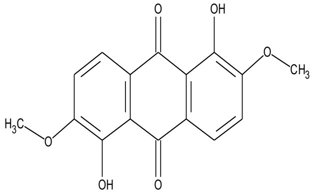	[synthetic]
5,7,10-Trihydroxy-2-methyl-1,4-anthraquinone	3062	-0.005	-30.070	3	C15 H10 O5	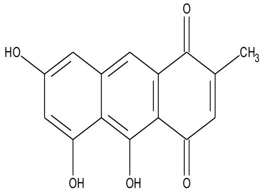	[F] Aspergillus cristatus (Eurotium cristatum)
11-Deoxy-bisanhydro-13-dihydrodaunomycinone	17199	-0.006	-39.060	4	C21 H16 O5	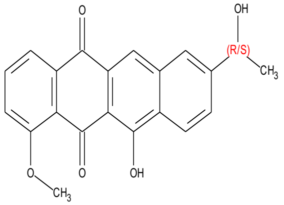	[B] Streptomyces sp. GW37/3236

Diboviquinone-3,4	16594	-0.008	-45.498	1	C47 H64 O8	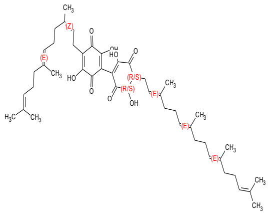	[F] Gomphidius rutilus
5-Hydroxydigitolutein	15323	-0.010	-26.576	3	C16 H12 O5	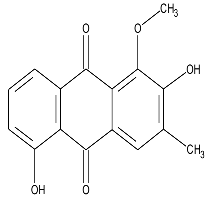	thomson
9-Ethyl-6-hydroxy-4-methoxynaphthacene-5,12-quinone	17197	-0.012	-30.601	4	C21 H16 O4	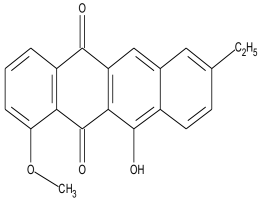	
1-Hydroxy-2,6,8-trimethoxy-9,10-anthraquinone	22561	-0.014	-45.291	3	C17 H14 O6	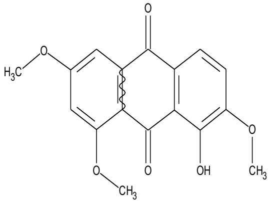	[B] bacteria Photorhabdus luminescens
Javanicin	5463	-0.014	-35.597	2	C15 H14 O6	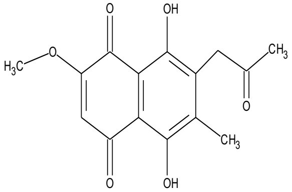	[F] Fusarium solani, F. decemcellulare cells [F] endophytic Chloridium sp.

DK 7814C	732	-0.015	-69.280	2	C26 H16 O14	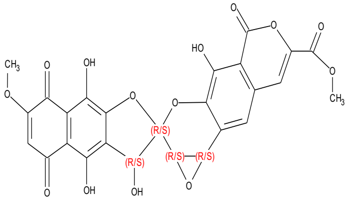	[B] Dactylosporangium purpureum ri-1 (FERM-p 5658)
5,10-Dihydroxy-7-methoxy-2-methyl-1,4-anthraquinone	4066	-0.020	-25.486	3	C16 H12 O5	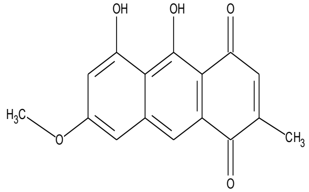	[F] Aspergillus cristatus (Eurotium cristatum)
5,10-Dihydroxy-2-methoxy-7-methyl-1,4-anthraquinone	20229	-0.020	-30.181	3	C16 H12 O5	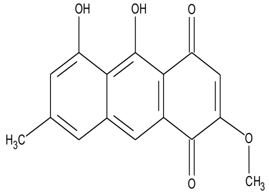	[synthetic]
5,8,8'-Trihydroxy-3',6-dimethyl-2,5'-dinaphthoquinone	20184	-0.022	-49.043	2	C22 H14 O7	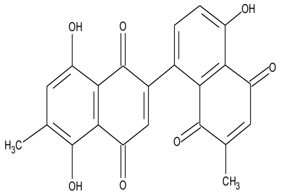	[synthetic]
DF-7814A	729	-0.022	-67.889	2	C26 H18 O15	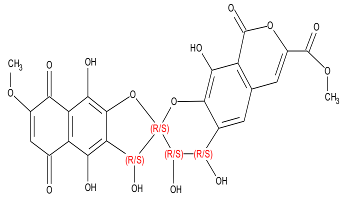	[B] Dactylosporangium purpureum ri-1 (FERM-p 5658)

DK 7814B	731	-0.027	-70.869	2	C26 H18 O14	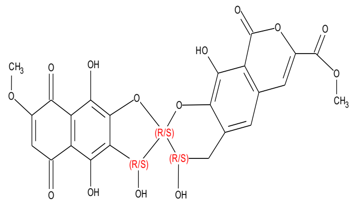	[B] Dactylosporangium purpureum ri-1 (FERM-p 5658)
Griseorhodin A	1763	-0.031	-60.125	2	C25 H16 O12	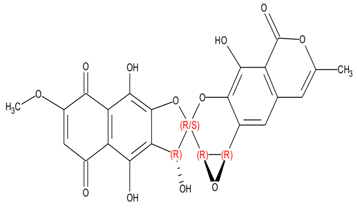	[B] Streptomyces griseus, S.californicus
11-O-Demethylpradimicinone II	7898	-0.032	-81.311	4	C27 H21 N1 O12	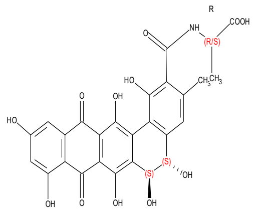	[B] Actinomadura verrucosospora subsp. neohibisca E-40, blocked mutant of Actinomadura hibisca p157-2
Kwanzoquinone A	39181	-0.036	-24.310	3	C18 H14 O4	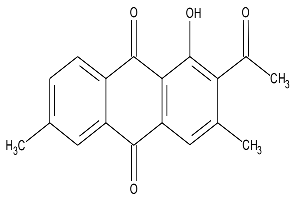	[Pl] Homerocallus fulva
Aristolindiquinone	15087	-0.038	-14.971	2	C12 H10 O4	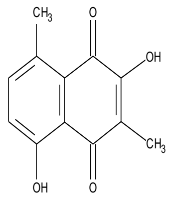	thomson

1-Hydroxyanthraquinone	9845	-0.038	-18.975	3	C14 H8 O3	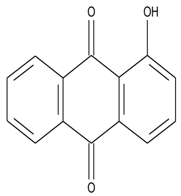	
Diazadiphenoquinone	19668	-0.038	-71.741	1	C12 H6 N2 O10	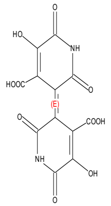	[B] Arthrobacter crystallopoietes
Griseorhodin G	751	-0.039	-61.643	2	C25 H18 O12	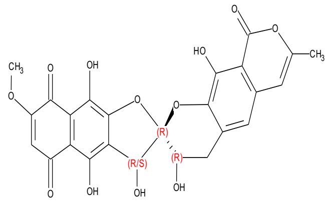	[B] Streptomyces griseus fcrc-57, S.californicus
(-)-Luteoskyrin	4897	-0.040	-50.951	4	C30 H22 O12	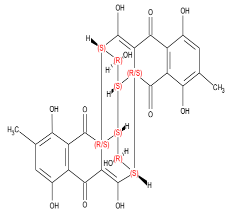	[F] Penicillium islandicum, Mycelia sterilia
Aurantio-obtusin	10161	-0.041	-33.226	3	C17 H14 O7	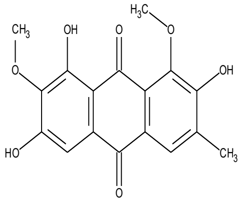	thomson

2-Acetyl-3,8-dihydroxy-6-methoxy anthraquinone (or 3-Acetyl-2,8-dihydroxy-6-methoxyanthraquinone)	23797	-0.043	-38.118	3	C17 H12 O6	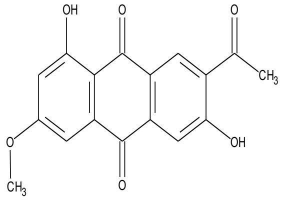	[F] Fusarium oxysporum
2-Acetoxy-1-hydroxy-anthraquinone	20146	-0.044	-27.970	3	C16 H10 O5	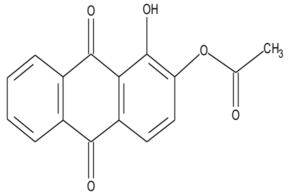	[synthetic]
Anhydroflavomannin-1,4-quinone-6,6'-di-O-methyl ether	17503	-0.047	-55.391	3	C32 H26 O10	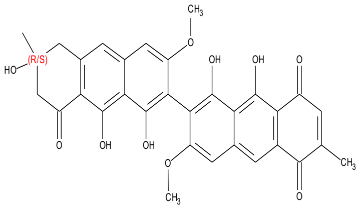	
Pachybasin	10665	-0.048	-20.626	3	C15 H10 O3	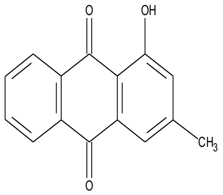	[F] Trichoderma harzianum rifai, T. hamatum
Ventiloquinone I	15189	-0.048	-27.547	3	C16 H16 O6	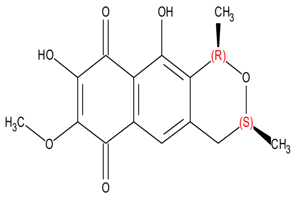	thomson

2,6-Diacetoxy-1-hydroxyanthraquinone	20150	-0.055	-35.855	3	C18 H12 O7	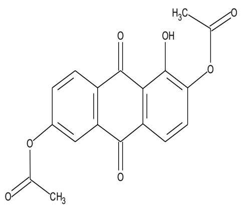	[synthetic]
Griseorhodin C	1764	-0.055	-64.381	2	C25 H18 O13	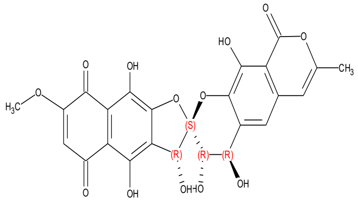	[B] Streptomyces californicus sp. 76, Actinomadura prunicolor, S.prunicolor, S.griseus
(7S)-5,6,7,8-Tetrahydro-7-hydroxy-2-methoxy-7-methyl-1,4- anthraquinone	8844	-0.056	-34.691	3	C16 H16 O6	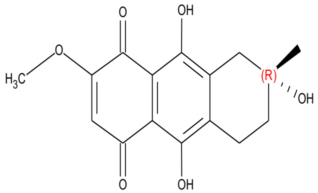	[F] Dermocybe splendida, D. umbonata
Ericamycin	12171	-0.056	-63.446	4	C28 H21 N1 O8	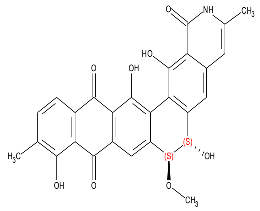	[B] Streptomyces varius
MT 81	836	-0.058	-32.580	3	C22 H18 O7	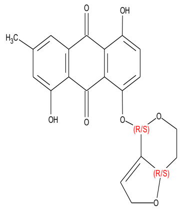	[F] Penicillium nigricans

1-Hydroxy-7-methylanthraquinone	20140	-0.062	-19.117	3	C15 H10 O3	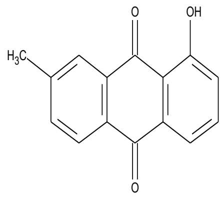	thomson
Fusarubinoic acid	23218	-0.062	-43.088	2	C15 H12 O8	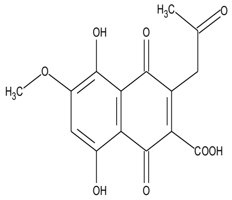	[F] Neocosmospora haematococca
2,5,7,8-Tetrahydroxy-1,4-naphthoquinone	3986	-0.063	-44.049	2	C10 H6 O6	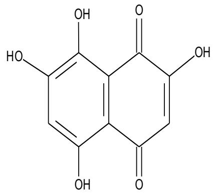	[An] Strongylocentrotus nudus
1-Hydroxy-2-methylanthraquinone	9844	-0.063	-18.812	3	C15 H10 O3	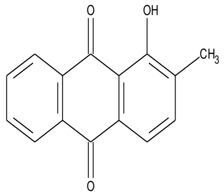	
Cuculoquinone	39882	-0.064	-29.159	2	C24 H18 O12	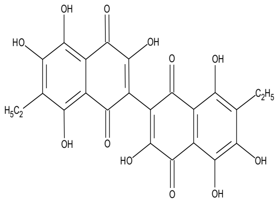	[L] Cetraria cucullata

Coriloxin	10260	-0.065	-25.702	1	C8 H8 O4	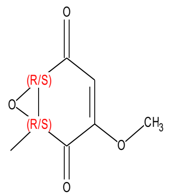	[F] Coriolus vernicipes
1-Hydroxy-6 or 7-hydroxymethylanthraquinone	15286	-0.068	-28.057	3	C15 H10 O4	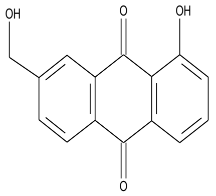	
Phenocyclinone	2607	-0.069	-78.777	4	C35 H24 O14	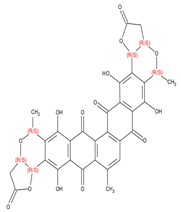	[B] Streptomyces coelicolor
4',10-Dihydroxy-4-oxoanhydro-flavomannin-9',10'-quinone 6,6'-dimethyl ether A	15899	-0.071	-55.465	3	C32 H24 O13	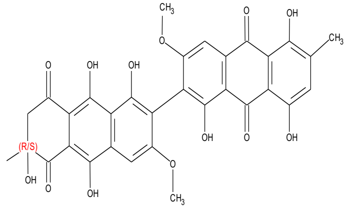	[F] Australian Dermocybe sp. WAT 21566
b-Naphthocyclinone	2921	-0.071	-74.395	4	C35 H32 O14	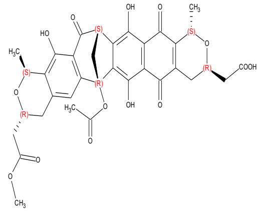	[B] Streptomyces arenae Tue 495

1,7-Dihydroxy-3-hydroxymethyl-9,10-anthraquinone	33809	-0.072	-39.160	3	C15 H10 O5	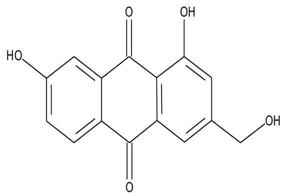	[F] Phoma sorghina from [Pl] Tithonia diversifolia (Asteraceae)
Terreic acid	5784	-0.077	-34.718	1	C7 H6 O4	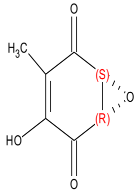	[F] Aspergillus terreus, Asp.paruvules
5,6-Dihydro-4,7,9,12-tetrahydroxy-2-methylbenzo[a]naphthacene-8,13-quinone	15467	-0.077	-45.582	4	C23 H16 O6	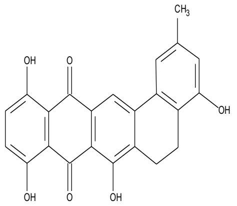	
1-Hydroxy-5-methoxy-2-methylanthraquinone	15297	-0.077	-20.939	3	C16 H12 O4	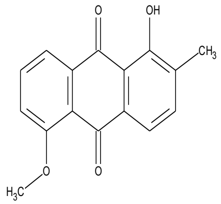	
5,8-Dihydroxy-2,6-dimethoxy-7-(2-oxopropyl)-1,4- naphthoquinone	5014	-0.077	-40.124	2	C15 H14 O7	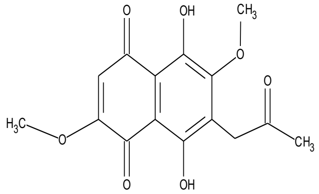	[F] Fusarium solani

5,8-Dihydroxy-2,7-dimethoxy-1,4-naphthoquinone	4068	-0.077	-28.516	2	C12 H10 O6	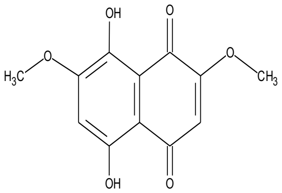	[B] Streptomyces sp., Streptoverticillium sp., Actinomadura sp.
Naphthoquinone 2	22940	-0.084	-32.163	3	C20 H23 N1 O6	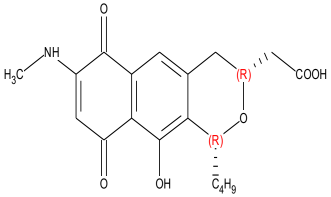	[B] Streptomyces sp.
Naphthoquinone 1	22939	-0.084	-33.484	3	C21 H25 N1 O6	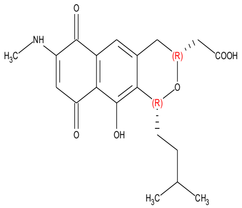	[B] Streptomyces sp.
3,8-Dihydroxy-1-methylanthraquinone-2-carboxylic acid	6937	-0.085	-37.939	3	C16 H10 O6	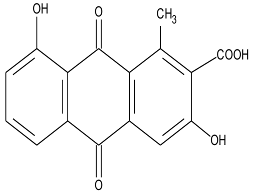	[B] Streptomyces sp. strain 3094; S. sp. AK671
Rhein	10738	-0.086	-38.212	3	C15 H8 O6	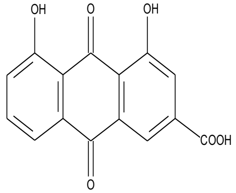	[Pl] Homerocallus fulva

Cervicarcin	1225	-0.088	-58.944	3	C19 H20 O9	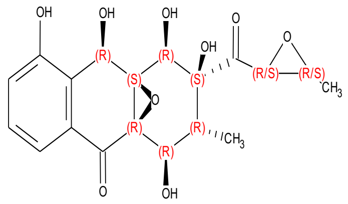	[B] Streptomyces ogaensis	1
Granatomycin D	1753	-0.088	-24.242	4	C22 H22 O10	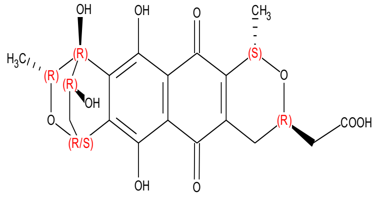	[B] Streptomyces thermoviolaceus subsp. pigens var. wr-141, S.lateritus	
4,9-Dihydroxyperylene-3,10-quinone	9987	-0.088	-30.979	4	C20 H10 O4	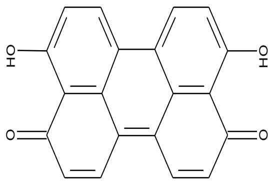	[F]	
Xanthopurpurin-3-methyl ether	17317	-0.089	-24.397	3	C15 H10 O4	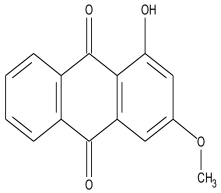		
1-Hydroxy-2-hydromethyl-5,6-dimethoxyanthraquinone	15325	-0.092	-31.218	3	C17 H14 O6	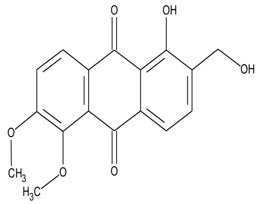		

1-Amino-8-hydroxy-anthraquinone	20144	-0.093	-26.628	3	C14 H9 N1 O3	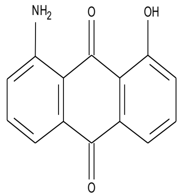	[synthetic]
6-Ethyl-2,5,7,8-tetrahydroxy-1,4-naphthoquinone	40217	-0.093	-27.193	2	C12 H10 O6	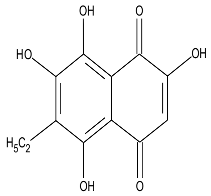	[L] Cetraria cucullata
8-Hydroxy-3-methoxy-1-methylanthraquinone-2-carboxylic acid methyl ester	12554	-0.095	-30.309	3	C18 H14 O6	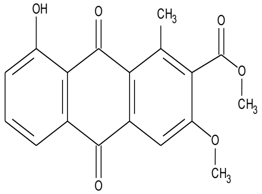	[B] Streptomyces sp. strain 3094
Solaniol	3485	-0.096	-35.419	2	C15 H16 O6	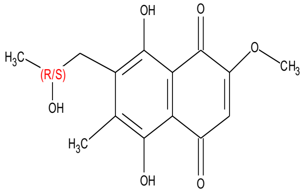	[F] Fusarium solani; "Munissi MUF2"
1-Hydroxy-7-methoxyanthraquinone	20156	-0.100	-25.281	3	C15 H10 O4	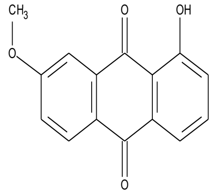	[synthetic]

2-(1-Hydroxyethyl)-3,8-dihydroxy-6-methoxyanthraquinone (or 3-(1- Hydroxyethyl)-2,8-dihydroxy-6-methoxyanthraquinone)	23798	-0.100	-42.077	3	C17 H14 O6	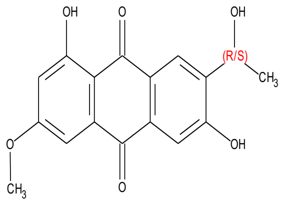	[F] Fusarium oxysporum
Granatomycin A	4370	-0.101	-26.424	4	C23 H24 O10	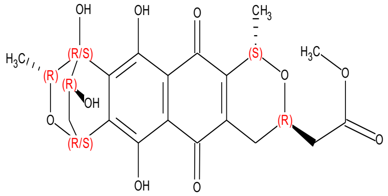	[B] Streptomyces lateritius zimet 43627
Methyl 8-hydroxy-3-methoxy-1-propylanthraquinone-2-carboxylate	37934	-0.101	-29.779	3	C20 H18 O6	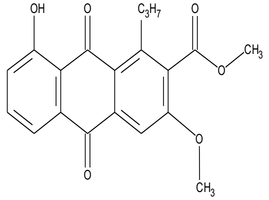	[B] Micromonospora rhodorangea
5-Hydroxyanhydroflavomannin-9,10-quinone-6,6'-di-O-methyl ether	17612	-0.104	-45.934	3	C32 H26 O11	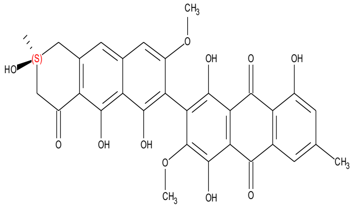	
1,3-Dihydroxy-2,5-dimethoxyanthraquinone	15377	-0.105	-27.726	3	C16 H12 O6	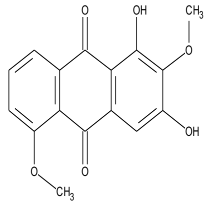	

1-Hydroxy-6-methoxyanthraquinone	20238	-0.107	-25.184	3	C15 H10 O4	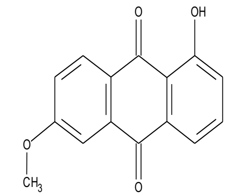	[synthetic]
1,6,8-Trhydroxy-3-propanoylanthraquinone	15369	-0.108	-36.936	3	C17 H12 O6	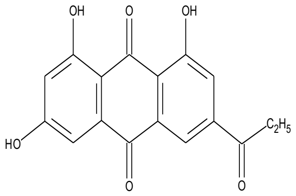	
Deflectin 1b	1370	-0.112	-22.974	2	C23 H28 O5	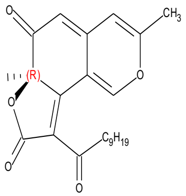	[F] Aspergillus deflectus
Basidifferquinone-B	9115	-0.112	-48.263	3	C23 H14 O8	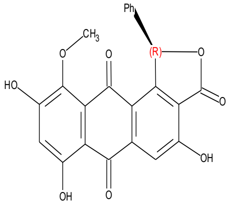	[B] Streptomyces sp. b-412
1,3-Dihydroxy-6-methyl-7-methoxyanthraquinone	34626	-0.112	-7.580	3	C16 H12 O5	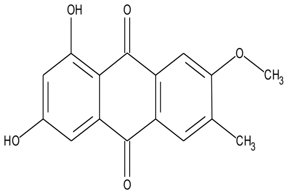	

Deflectin 1a	1369	-0.112	-19.991	2	C21 H24 O5	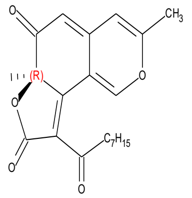	[F] Aspergillus deflectus
1,4-Dihydroxy-2,3-dimethoxy-anthraquinone	20120	-0.113	-26.699	3	C16 H12 O6	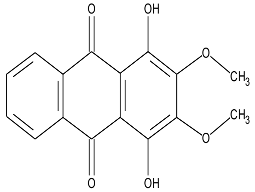	[synthetic]
5,6-Dihydro-4,7,9,12-tetrahydroxy-2-methylbenzo[a]naphthacene- 8,13-quinone-10 or 11-carboxylic acid	15468	-0.114	-53.837	4	C24 H16 O8	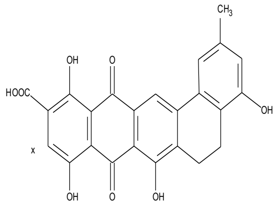	
3-O-Methylfusarubin	20078	-0.116	-26.854	3	C16 H16 O7	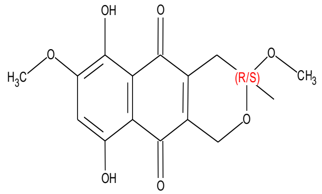	[F] Fusarium moniliforme
O-Ethylfusarubin	10636	-0.117	-25.486	3	C17 H18 O7	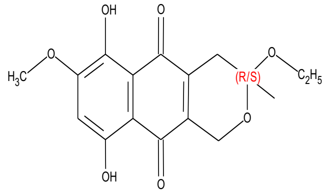	[F] Fusarium solani

1-Bromo-4,5-dihydroxy-7-methoxy-2-methylanthraquinone	20261	-0.117	-27.755	3	C16 H11 Br1 O5	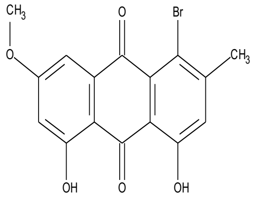	[synthetic]
1,3-Dihydroxy-5,6-dimethoxy-2-methoxymethyl-anthraquinone	42432	-0.122	-32.074	3	C18 H16 O7	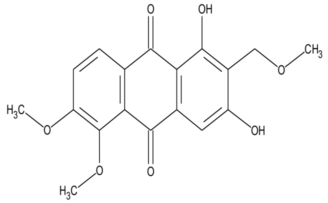	[Pl] Prismatomeris malayana
Aureoquinone	26020	-0.122	-42.290	2	C12 H10 O6	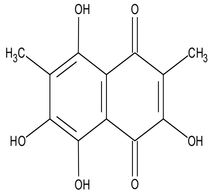	[F] Aureobasidium sp.
1,3,6-Trihydroxy-2-methylanthraquinone	15328	-0.123	-35.282	3	C15 H10 O5	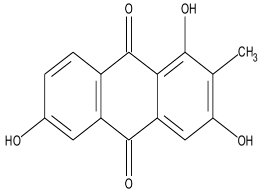	thomson
1-Hydroxy-2,5-dimethoxy-anthraquinone	20115	-0.123	-29.663	3	C16 H12 O5	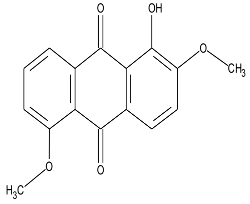	[synthetic]

1,3-Dihydroxy-6-hydroxymethyl-7-methoxyanthraquinone	31743	-0.123	-40.074	3	C16 H12 O6	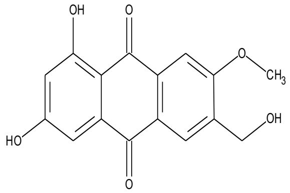	[F] Penicillium sp.	
MM 14201	833	-0.124	-36.385	1	C6 H7 N1 O3	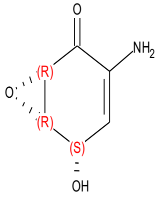	[B] Streptomyces sp. (ncib 11813)	1
Catenarin-5-methylether	4171	-0.125	-32.194	3	C16 H12 O6	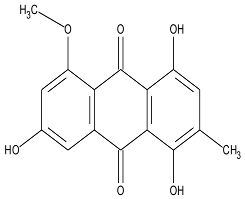	[F] Aspergillus ruber, Asp.glaucus, Asp.cristatus (Eurotium cristatum)	
Novarubin	5577	-0.126	-43.868	2	C15 H14 O7	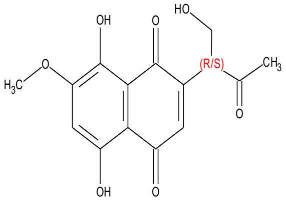	[F] Fusarium decemcellulare cells, Fus.martici-pisi, Fus.solani, Neocosmospora vasinfecta	
dimeric Ventiloquinone	23297	-0.127	-47.979	3	C31 H28 O11	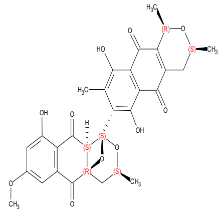	[Pl] Ventilago goughii	

1-Hydroxy-3,7-dimethoxy-6-methylanthraquinone	3942	-0.130	-30.118	3	C17 H14 O5	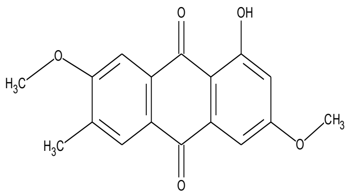	[F] Macrosporium porri, Phomopsis juniperovora
3,8-Dimethoxy-1-hydroxy-9,10-anthraquinone	19154	-0.130	-33.158	3	C16 H12 O5	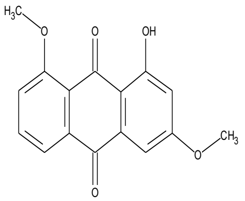	[B] entomopathogenic bacterium Photorhabdus luminescens
1-Hydroxy-3,7-dimethoxy-anthraquinone	20143	-0.131	-31.465	3	C16 H12 O5	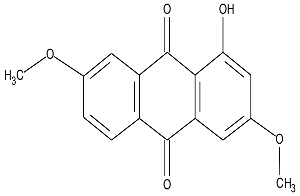	[synthetic]
1,3-Dihydroxy-5,6-dimethoxy-2-methyl-anthraquinone	42430	-0.131	-31.730	3	C17 H14 O6	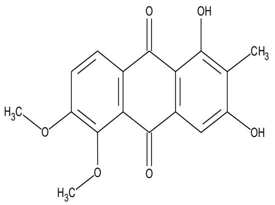	[Pl] Prismatomeris malayana
Anhydropseudophlegmacin-9,10-quinone-3-amino-8-O-methyl ether	40239	-0.131	-85.617	3	C30 H21 N1 O8	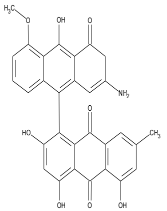	[F] Phoma herbarum FGCC#54

Basidifferquinone	7131	-0.131	-45.398	3	C24 H16 O8	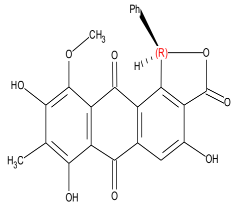	[B] Streptomyces rubiginosus b-412
Rubellin B	3452	-0.132	-59.335	3	C30 H22 O10	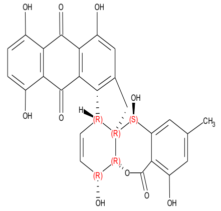	[F] Mycosphaerella rubella
3-Amino-1-hydroxy-2-methoxyanthraquinone	20153	-0.133	-31.307	3	C15 H11 N1 O4	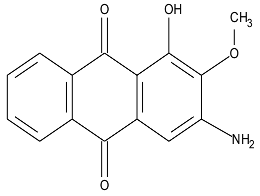	[synthetic]
2061-A	67	-0.133	-54.113	1	C7 H6 N2 O4	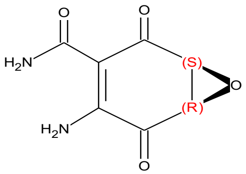	[B] Streptomyces no. 2061 fce, S. sp. (nclb 11306)
K-1115-A	22527	-0.138	-32.013	3	C18 H14 O6	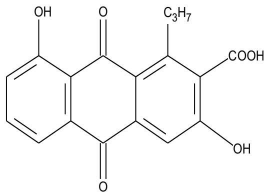	[B] Actinomycete sp. Mer-K1115; marine Streptomyces spp. FX-58, B8000

Eleuthraquinone A	40367	-0.141	######	3	C18 H14 O5	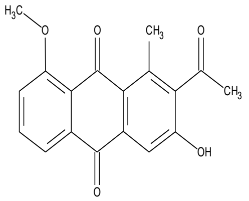	[Pl] Eleutherine americana
3,5,6-Trihydroxy-2-methylanthraquinone	15327	-0.141	-31.851	3	C15 H10 O5	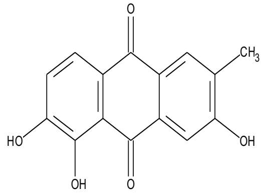	thomson
1,6-Dihydroxy-8-propylanthraquinone	33619	-0.142	-30.210	3	C17 H14 O4	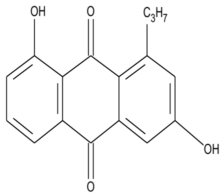	[B] marine Streptomyces spp. FX-58, B8000
Demethylmacrosporin	4216	-0.143	-38.561	3	C15 H10 O5	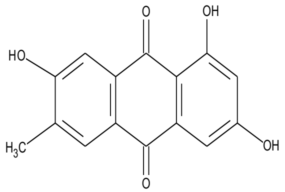	[F] Macrosporium porri; Alternaria solani, Dichotomophthora lutea
2-Hydroxynorjavanicin	6921	-0.144	-37.044	2	C14 H12 O7	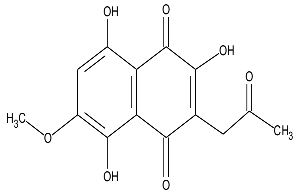	[F] Fusarium solani, Nectria haematococca (fungus)

1-Hydroxy-2,7-dimethoxy-anthraquinone	20243	-0.146	-33.786	3	C16 H12 O5	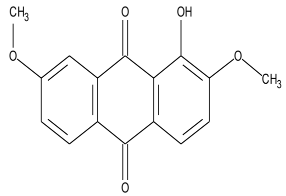	[synthetic]
1-Amino-4-hydroxy-anthraquinone	20141	-0.146	-25.581	3	C14 H9 N1 O3	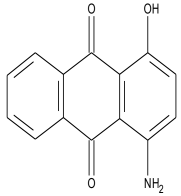	[synthetic]
5,7-Dihydroxy-2-[1-(4-methoxy-6-oxo-6H-pyran-2-yl)-2-phenylethylamino]-[1,4]naphthoquinone	35754	-0.148	-52.159	2	C24 H19 N1 O7	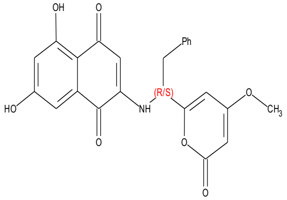	[F] endophytic Aspergillus niger EN-13
(3'R,P)-Anhydropseudophlegmacin-9,10-quinone-6',8'-di-O-methyl ether	23602	-0.148	-76.758	3	C32 H26 O10	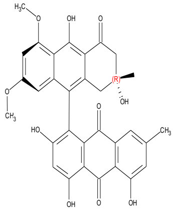	[F] fungus Dermocybe sp.
4-Hydroxy-4'-oxoanhydro-flavomannin-9,10-quinone	17626	-0.148	-57.943	3	C30 H20 O12	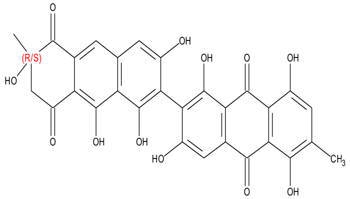	

2,3-Dimethoxy-5,6-dimethyl-2-cyclohexene-1,4-dione	1031	-0.150	-26.576	1	C10 H14 O4	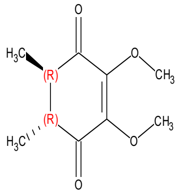	[F] Gliocladium roseum
1-Hydroxy-6-methoxy-8-methylanthraquinone	22350	-0.151	-24.662	3	C16 H12 O4	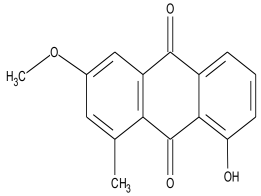	[B] Streptomyces sp. GW 24/1694
1-Hydroxy-2,6-dimethoxyanthraquinone	20242	-0.152	-31.320	3	C16 H12 O5	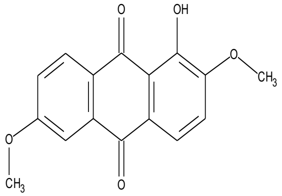	[synthetic]
9'-Hydroxyaloesaponarin II	31052	-0.152	-36.466	3	C15 H10 O5	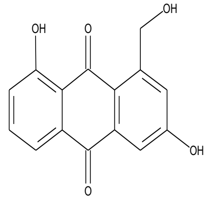	[B] recombinant Streptomyces lividans K4-114
1-Hydroxy-6-methoxy-8-propylanthraquinone	33618	-0.156	-24.924	3	C18 H16 O4	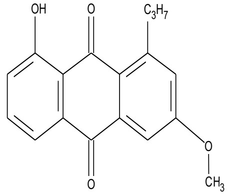	[B] marine Streptomyces sp. B8000

2-Acetyl-1,8-dihydroxy-3-methyl-anthraquinone	29567	-0.157	-24.399	3	C17 H12 O5	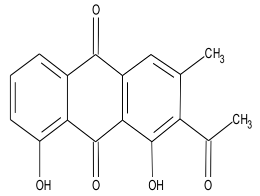	[B] degradation product from metabolite of Streptomyces aureofaciens, mutant ED 1369; [B] marine Streptomyces sp. Mei6 1,2; [B] terrestrial Streptomyces sp. Eg5
Julichrome-Q2,3	10483	-0.157	-84.049	3	C38 H34 O14	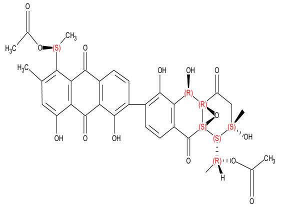	[B] Streptomyces shinodaensis
1,4-Dihydroxy-5,7-dimethoxyanthraquinone	20258	-0.161	-34.431	3	C16 H12 O6	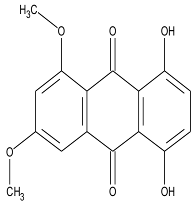	[synth.]
Chrysophanol	5285	-0.162	-19.878	3	C15 H10 O4	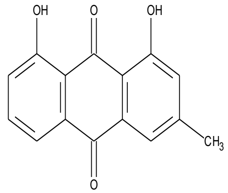	[F] Monilinia fructicola; Trichoderma harzianum rifai, Trichoderma hamatum
O-Demethylfusarubin	6851	-0.163	-40.050	3	C14 H12 O7	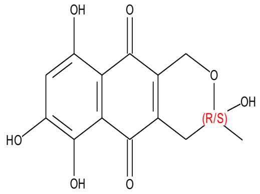	[F] Fusarium decemcellulare

Fumigatin oxide	5408	-0.172	-27.571	1	C8 H8 O5	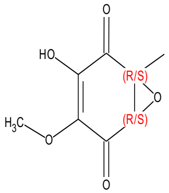	[F] Aspergillus fumigatus
Antibiotic Zg	303	-0.172	-49.150	4	C22 H18 O8	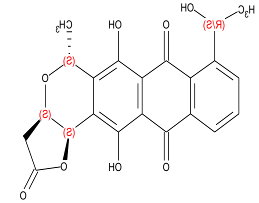	[B]Streptomyces thermoviolaceus subst. pigens var. wr-141
1,3-Dihydroxy-6-methoxy-2-methoxymethylanthraquinone	39170	-0.176	-31.021	3	C17 H14 O6	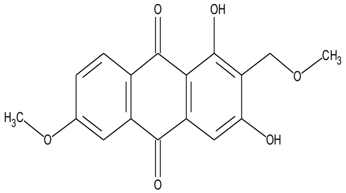	[Pl] Prismatomeris tetranga
1,4,6-Trihydroxy-5-methoxy-2 or 3-methylanthraquinone	15390	-0.176	-25.667	3	C16 H12 O6	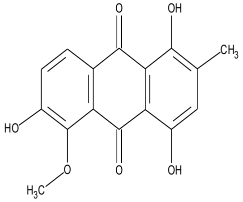	as a glycoside 10 roots of Cassia nodosaZ89 (Leguminosae). Thomson
3-Methyl-5,6(7),8-trihydroxy-2-aza-(9,10)-anthraquinone	6943	-0.179	-34.158	3	C14 H9 N1 O5	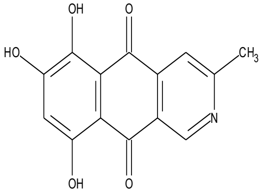	[F] Tolypocladium inflatum DSM 915

2,3,5,8-Tetrahydroxy-6-methyl-1,4-naphthoquinone	2992	-0.185	-26.568	2	C11 H8 O6	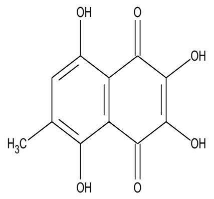	[B] Streptomyces filipensis MD 157-A9
Rubellin D	6711	-0.185	-60.012	3	C30 H22 O10	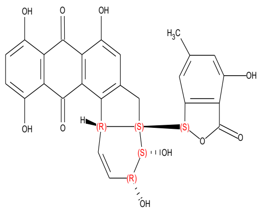	[F] Mycosphaerella rubella
1,3-Dihydroxy-6,8-dimethoxy-2-methylanthraquinone	15423	-0.185	-39.499	3	C17 H14 O6	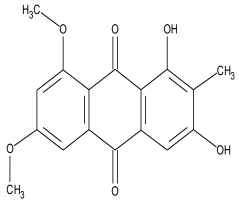	thomson
S 383-O	6712	-0.186	-31.018	3	C20 H16 O7	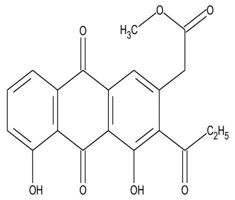	[B] Streptomyces galilaeus
1-Amino-5-hydroxy-anthraquinone	20145	-0.187	-24.817	3	C14 H9 N1 O3	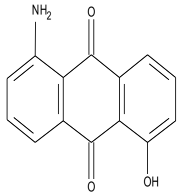	[synthetic]

Metabolite-III	12892	-0.188	-44.873	3	C21 H18 O9	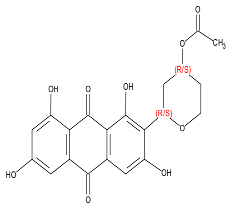	[F] Aspergillus versicolor
1,8-Dihydroxy-2-ethyl-3-methylanthraquinone	34409	-0.192	-19.991	3	C17 H14 O4	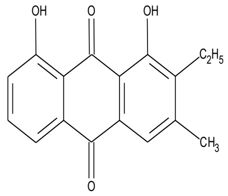	[B] marine Streptomyces sp. FX-58<br>[B] Micromonospora rhodorangea
Bostrycoidin	5246	-0.192	-29.262	3	C15 H11 N1 O5	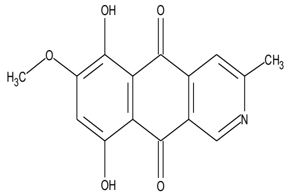	[F] Fusarium solani, F oxysporum,<br>F. decemcellulare cells<br>Fus.bostrycoides, Fus.solani-purple
1,8-Dihydroxy-5-methylanthraquinone	26057	-0.198	-17.872	3	C15 H10 O4	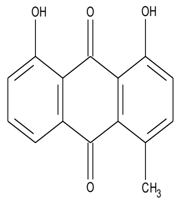	[C] Cyanobacterium Nostoc commune (EAWAG 122b)
1,8-Dihydroxy-2,7-dimethoxyanthraquinone	20135	-0.201	-36.044	3	C16 H12 O6	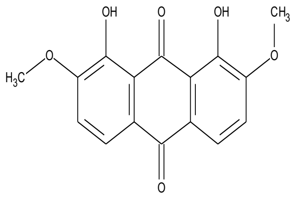	[synthetic]

2-Acetoxy-1,5-dihydroxyanthraquinone	20151	-0.202	-26.804	3	C16 H10 O6	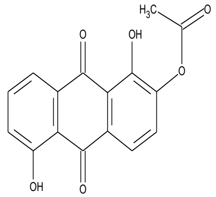	[synthetic]
1,5-Dihydroxyanthraquinone	20251	-0.202	-18.111	3	C14 H8 O4	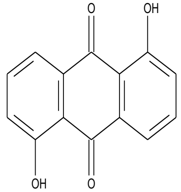	[synth.]
Julichrome Q2,5	1917	-0.208	-78.730	3	C36 H26 O11	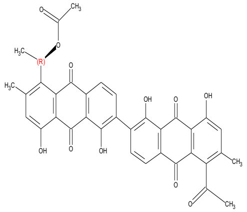	[B] Streptomyces shinodaensis
Blanchaquinone	35536	-0.212	-33.780	3	C22 H20 O7	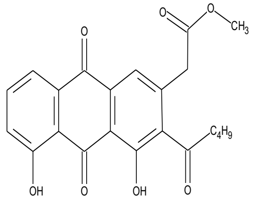	[B] Streptomyces sp.
a2-Rhodomycin-A	10084	-0.214	-72.680	4	C40 H51 N1 O15	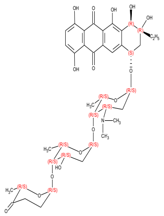	

1,6,8-Trihydroxy-3-(2-hydroxypentyl)anthraquinone	15371	-0.215	-37.144	3	C19 H18 O6	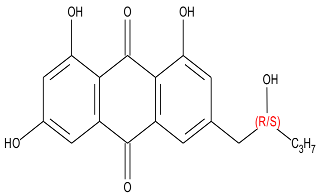	
3,7-Diacetoxy-1,5-dihydroxy-anthraquinone	20127	-0.217	-30.118	3	C18 H12 O8	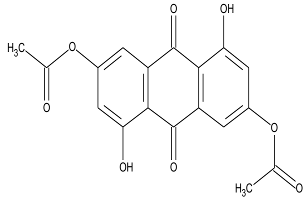	[synthetic]
1,4,5-Trimethoxy-8-methoxy-anthraquinone	20109	-0.217	-27.676	3	C15 H10 O6	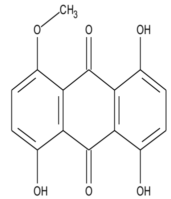	[synthetic]
Ekatetrone	1470	-0.220	-60.608	4	C19 H13 N1 O7	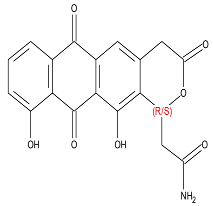	[B] Streptomyces aureofaciens 1717, 111158, 84(54
4-Hydroxyanhydroflavomannin-9,10-quinone-6,6'-di-O-methyl ether	17610	-0.220	-38.472	3	C32 H26 O11	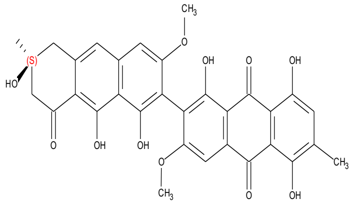	

Stemphyltoxin III	3525	-0.221	-94.809	4	C20 H12 O6	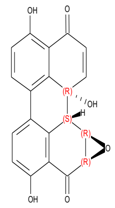	[F] Stemphylium botryosum var. lactucum
Methyl 3,4,8-trihydroxy-1-methylanthraquinone-2-carboxylate 3-methyl ether	15342	-0.222	-31.010	3	C18 H14 O7	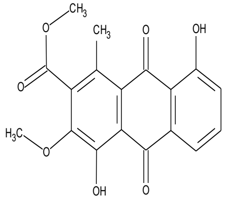	[B] Streptomyces sp. 3094
Averythrin 6-methyl ether	10167	-0.223	-30.105	3	C21 H20 O6	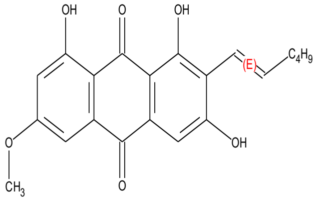	[L] Herpotrichia rhodosticta; lichen Solorina crocea
Evariquinone	28767	-0.223	-36.865	3	C16 H12 O6	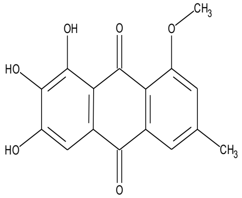	[F] Emericella variecolor
2,3,6,7-Tetraacetoxy-1,5-dihydroxyanthraquinone	20130	-0.228	-57.247	3	C22 H16 O12	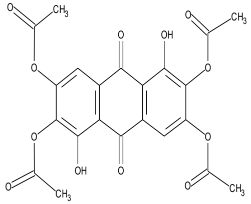	[synthetic]

Galvaquinone A	41671	-0.228	-28.059	3	C21 H20 O5	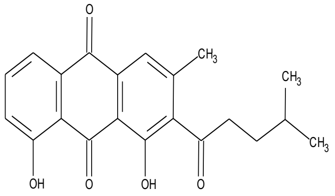	[B] marine-derived Streptomyces spinoverrucosus
1,5-Dihydroxy-2-methylanthraquinone	15296	-0.229	-17.993	3	C15 H10 O4	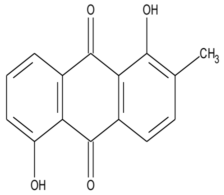	
3-Methoxychrysazin	15330	-0.231	-24.515	3	C15 H10 O5	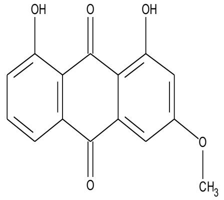	[B] bacteria Photorhabdus luminescens
1,8-Dihydroxy-2-methoxy-6-methylanthraquinone	34730	-0.233	-28.713	3	C16 H12 O5	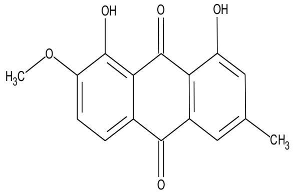	[F] marine sea urchin-derived Monodictys sp.
1,3,8-Trihydroxy-6-methoxyanthraquinone	28897	-0.233	-32.938	3	C15 H10 O6	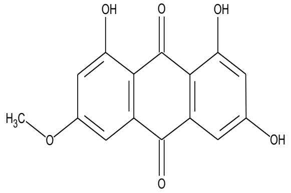	[F] fungus Curvularia lunata

4,5-Dihydroxy-2-methoxycarbonylmethyl-3-(3-oxopentyl)anthraquinone	15318	-0.238	-38.992	3	C22 H20 O7	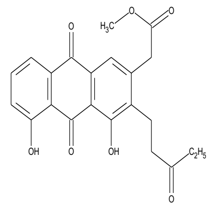	
1,4-Dihydroxy-6-methoxy-anthraquinone	20147	-0.238	-22.443	3	C15 H10 O5	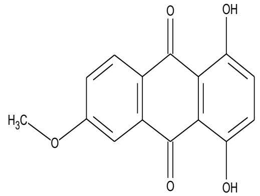	[synthetic]
Komodoquinone B	30422	-0.239	-55.005	4	C19 H16 O7	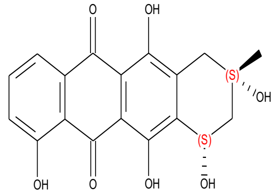	[B] marine Streptomyces sp. KS3, S. sp. GW10/1811
Dermoquinone	16274	-0.241	-17.694	3	C18 H14 O6	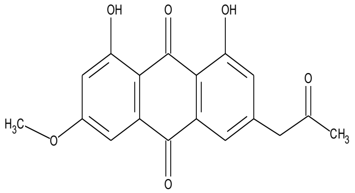	[F] Dermocybe sanguinea (sensu Cleland)
1,3,6,8-Tetrahydroxyanthraquinone	5898	-0.244	-38.835	3	C14 H8 O6	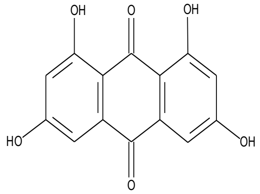	[F] endophytic conyothyrium sp. CAFT93 [F] Leptographium wageneri; Aspergillus versicolor, Rheum hotaoense, [F] Rumex alpinus, Trichoderma viride and Verticicladiella sp.

1,8-Dihydroxy-3,6-dimethoxy-anthraquinone	20247	-0.247	-28.180	3	C16 H12 O6	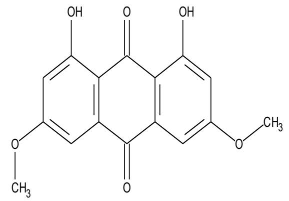	[synthetic]
1,5-Dihydroxy-3-methoxy-anthraquinone	20117	-0.249	-23.191	3	C15 H10 O5	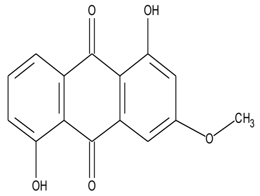	[synthetic]
Kwanzoquinone D	39186	-0.249	-51.306	3	C22 H22 O9	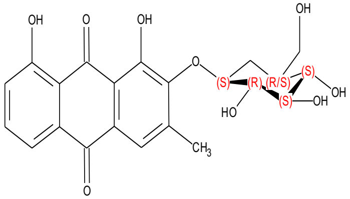	[Pl] Homerocallus fulva
2-n-Butyl-1,4-dihydroxy-anthraquinone	20149	-0.251	-18.875	3	C18 H16 O4	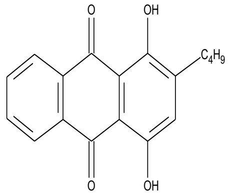	[synthetic]
1,5-Dihydroxy-3-methoxy-7-methylanthraquinone	42314	-0.259	-24.706	3	C16 H12 O5	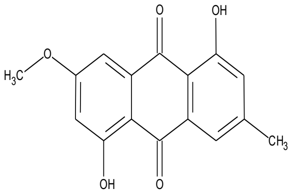	[F] endophytic Aspergillus wentii from red alga

1,3,8-Trihydroxy-6-hydroxymethylanthraquinone	3935	-0.265	-41.071	3	C15 H10 O6	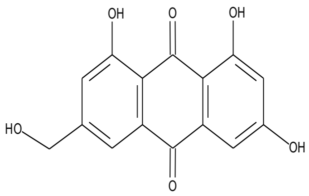	[F] Penicillium spp.; Preussia multispora
1,4-Dihydroxy-2,5-dimethoxy-9,10-anthraquinone	22562	-0.267	-35.563	3	C16 H12 O6	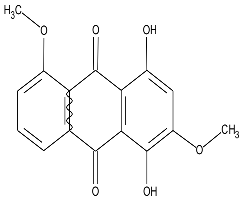	[B] bacteria Photorhabdus luminescens
Paeciloquinone-B	15941	-0.268	-59.175	3	C20 H16 O9	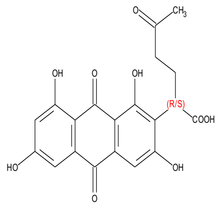	[F] Paecilomyces carneus
Kwanzoquinone E	39187	-0.270	-30.097	3	C15 H10 O6	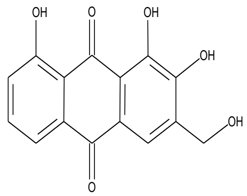	[Pl] Homerocallus fulva
Julichrome Q5,5	1920	-0.272	-50.980	3	C34 H22 O10	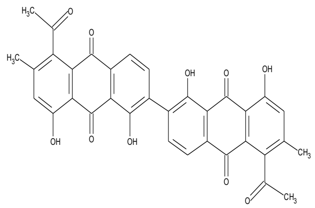	[B] Streptomyces shinodaensis

1,4-Dihydroxy-7-hydroxymethyl-2,5-dimethoxyanthraquinone	7887	-0.272	-48.738	3	C17 H14 O7	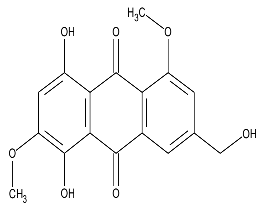	[F] Dermocybe WAT 22963
Vismiaquinone	15347	-0.273	-25.486	3	C21 H20 O5	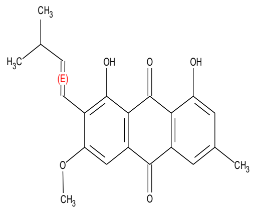	[Pl] Cordia aurantiaca
Emodin	5358	-0.275	-30.722	3	C15 H10 O5	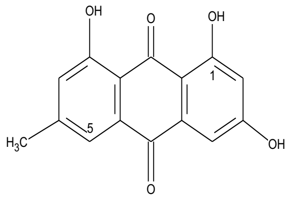	[F] endophytic Conyothyrium sp. CAFT93 [F] Penicillium islandicum, P. clavariae-formis, P.brunneum, P.avellaneum, Aspergillus ochraceus, A.wentii, Phoma foveata, Cladosporium fulvum,
Norsolorinic acid	10634	-0.277	-45.464	3	C20 H18 O7	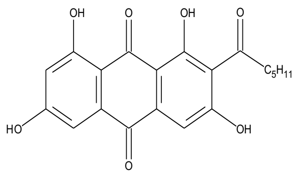	[F] Aspergillus versicolor, A.parasiticus, Sorlorina crocea
9-Ethyl-4,6-dihydroxynaphthacene-5,12-quinone	17198	-0.280	-20.860	4	C20 H14 O4	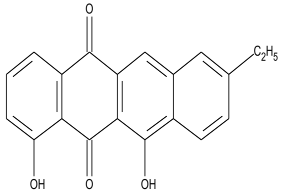	

3,5,8-Trihydroxy-7-methoxy-2-methylanthraquinone	39142	-0.284	-36.135	3	C16 H12 O6	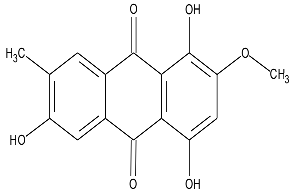	[F] endophytic Halorosellina sp.
5-Acetyl-2-methoxy-1,4,6-trihydroxy-anthraquinone	38778	-0.285	-48.518	3	C17 H12 O7	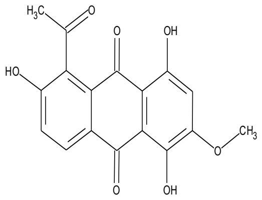	[F] marine endophytic Fusarium sp. No. b77
2-Acetoxy-1,4,8-trihydroxyanthraquinone	20152	-0.288	-24.659	3	C16 H10 O7	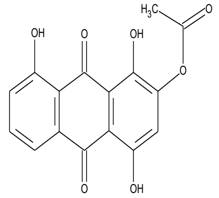	[synthetic]
Sterequinone C	41931	-0.288	-30.141	3	C19 H16 O3	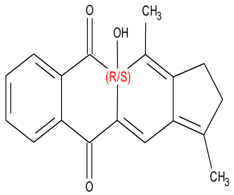	[F] mangrove endophytic Penicillium sp. ZH16
Paeciloquinone-C	15942	-0.292	-40.462	3	C15 H10 O7	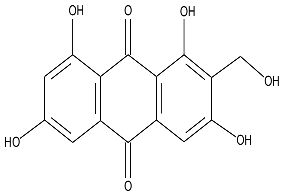	[F] Paecilomyces carneus

Physcion	4646	-0.293	-25.148	3	C16 H12 O5	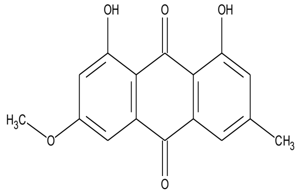	[F] Aspergillus ruber ifo 6004; Eurotium repens, Asp.glaucus, Penicillium herqueii, Rheum sp., Rumex sp., Asp.cristatus (Eurotium cristatum)
Javanicunine A	33857	-0.296	-39.420	1	C24 H30 N2 O4	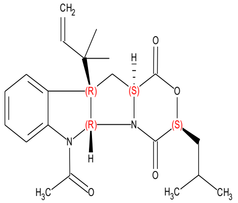	[F] Eupenicillium javanicum
1,2,3,8-Tetrahydroxy-6-methylanthraquinone	2958	-0.296	-28.682	3	C15 H10 O6	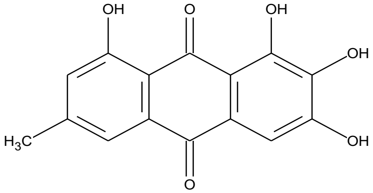	
Emodin 6-geranyl ether	9248	-0.299	-28.818	3	C25 H26 O5	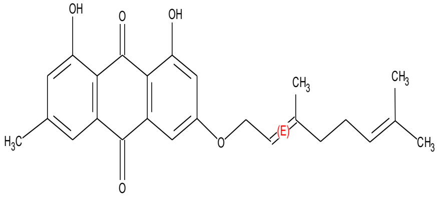	[Pl] Psorospermum febrifugum, Vismia laurentii, Cordia aurantiaca
1,3,8-Trihydroxy-6-methoxy-2-methylanthraquinone	15422	-0.303	-30.346	3	C16 H12 O6	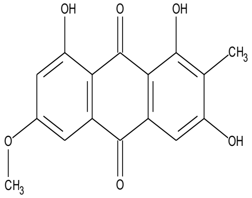	

2-n-Butyl-1,4-dihydroxy-3-methoxyanthraquinone	20154	-0.305	-18.983	3	C19 H18 O5	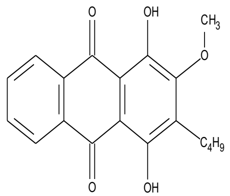	[synthetic]
1,8-Dihydroxy-3,6-dimethoxy-2-methyl-7-vinylanthraquinone	15424	-0.306	-2.082	3	C19 H16 O6	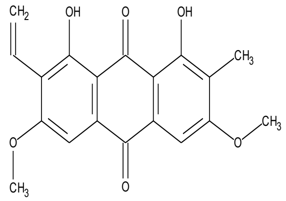	
Pradimicin-N	7628	-0.308	-72.368	4	C29 H25 N1 O12	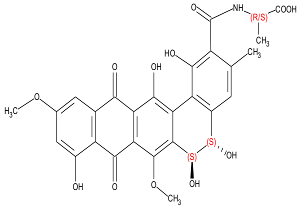	[B] Actinomadura mh193-16f4, blocked mutant of Actinomadura hibisca p157-2
Paeciloquinone-E	15944	-0.309	-34.006	4	C20 H16 O7	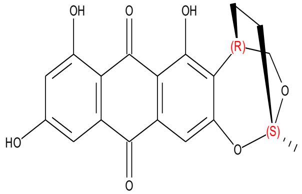	[F] Paecilomyces carneus
Julichrome Q5,6	1921	-0.311	-56.862	3	C37 H32 O11	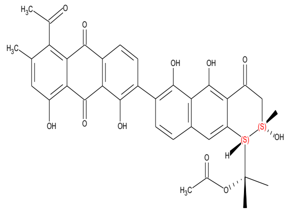	[B] Streptomyces shinodaensis

1,3,5-Trihydroxy-6,7-dimethoxy-2-methylanthraquinone	15436	-0.312	-31.601	3	C17 H14 O7	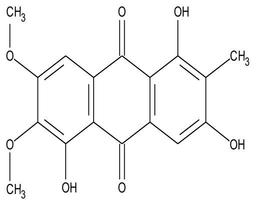	
Paeciloquinone-D	15943	-0.313	-41.962	3	C18 H14 O9	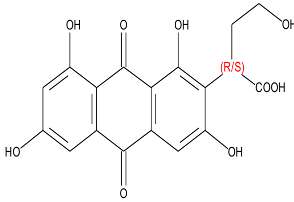	[F] Paecilomyces carneus
1,3,6,8-Tetrahydroxy-2-(1-methoxyhexyl)anthraquinone	2962	-0.314	-34.607	3	C21 H22 O7	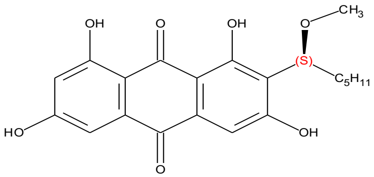	[L] [F] Aspergillus versicolor, Solorina crocea
1,3,6,8-Tetrahydroxy-2-methoxyethylanthraquinone IV	25600	-0.316	-35.261	3	C17 H14 O7	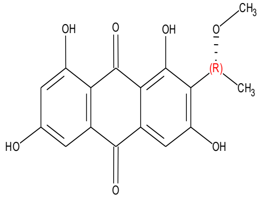	[F] sponge-associated marine fungus Microsphaeropsis sp.
1,2,8-Trihydroxy-6-methoxy-3-methylanthraquinone	3933	-0.317	-25.786	3	C16 H12 O6	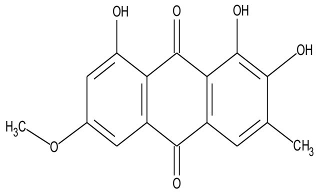	[F] Alternaria solani; Alternaria porri

1,3,5,8-Tetrahydroxy-6,7-dimethoxy-2-methylanthraquinone	17201	-0.325	-34.229	3	C17 H14 O8	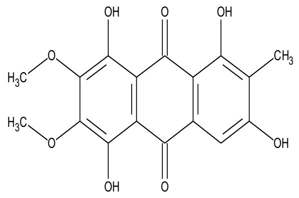	
4,5,8-Trihydroxy-2-methoxycarbonylmethyl-3-(3-oxopentyl)anthraquinone	15374	-0.330	-37.805	3	C22 H20 O8	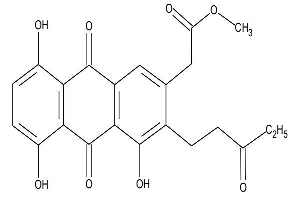	[B] marine Streptomyces sp. B7486
1,5-Dihydroxy-2,3,6,7-tetramethoxyanthraquinone	20129	-0.331	-28.734	3	C18 H16 O8	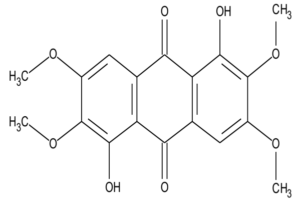	[synthetic]
Julichrome Q2,2	1916	-0.331	-61.598	3	C38 H30 O12	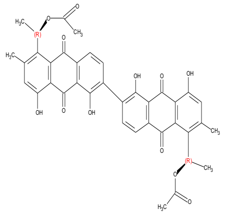	[B] Streptomyces shinodaensis
Paeciloquinone-A	15940	-0.332	-42.904	3	C18 H12 O8	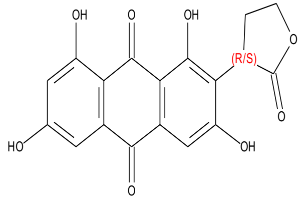	[F] Paecilomyces carneus

1,4,5-Trihydroxyanthraquinone	20257	-0.333	-18.326	3	C14 H8 O5	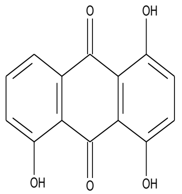	[synthetic]
1,4,5-Trihydroxy-6-acetoxyanthraquinone	20123	-0.337	-28.264	3	C16 H10 O7	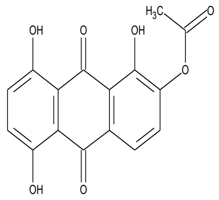	[synthetic]
1-Amino-4-hydroxy-2-methoxyanthraquinone	20133	-0.339	-30.097	3	C15 H11 N1 O4	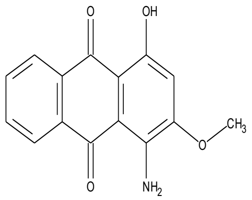	[synthetic]
1,3,6,7-8-Pentahydroxy-2-methoxyethylanthraquinon e V	25601	-0.350	-39.578	3	C17 H14 O8	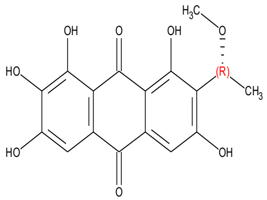	[F] sponge-associated marine fungus Microsphaeropsis sp.
Paeciloquinone-F	15945	-0.352	######	4	C20 H14 O9	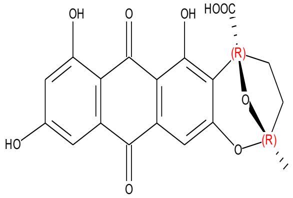	[F] Paecilomyces carneus

A-80915-G	7062	-0.352	-35.463	2	C25 H30 O5	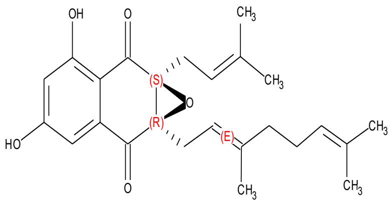	[B] Streptomyces aculeolatus NRRL 18422; alkaline treatment of naphthomevalin
1,4-Dihydroxy-2,5,8-trimethylanthraquinone	20128	-0.354	-16.378	3	C17 H14 O4	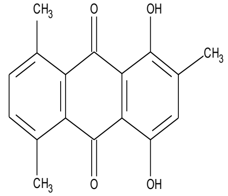	[synthetic]
Napyradiomycin C2	2333	-0.357	-41.738	3	C25 H27 Cl3 O5	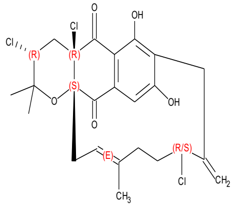	[B] Chainia rubra mg802-af1
(+)-3,3',7,7',8,8'-Hexahydroxy-5,5'-dimethyl-bisanthraquinone	37623	-0.361	-33.061	3	C30 H18 O10	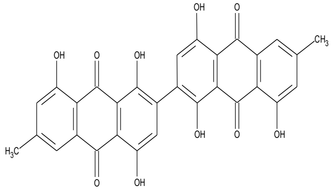	[F] mangrove endophytic fungus no 2240
1,3,5-Trihydroxy-8-methoxy-2-methyanthraquinone	15420	-0.363	-35.395	3	C16 H12 O6	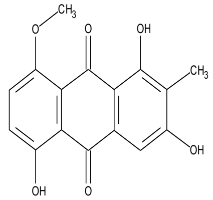	

Galvaquinone B	41672	-0.365	-24.000	3	C21 H20 O6	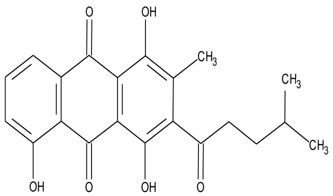	[B] marine-derived Streptomyces spinoverrucosus
1,4,5-Trihydroxy-2-methoxy-anthraquinone	20116	-0.367	-25.835	3	C15 H10 O6	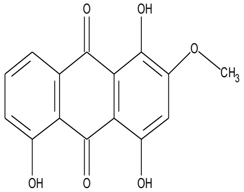	[synthetic]
Isofusarubin	1875	-0.372	-37.278	2	C15 H14 O7	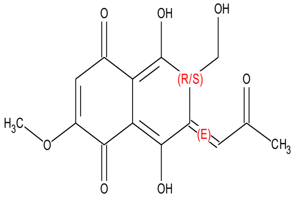	[F] Fusarium sp.
Basidiodifferoquinone	9117	-0.375	-45.700	3	C24 H18 O8	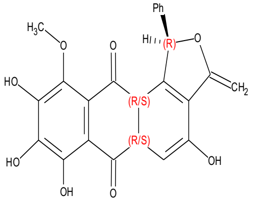	[B] Streptomyces sp.
1,4,5-Trihydroxy-2-methylanthraquinone	9837	-0.375	-18.321	3	C15 H10 O5	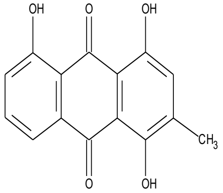	[F] Penicillium islandicum

1,4,5,7-Tetrahydroxy-2-methylanthraquinone	3937	-0.378	-28.393	3	C15 H10 O6	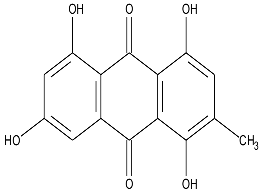	[F] Aspergillus ruber ifo 6004, Asp.amstelodami, Penicillium islandicum, Helminthosporium catenarii, Asp.cristatus (Eurotium cristatum)
Nanaomycin E	2319	-0.379	-24.407	3	C16 H14 O7	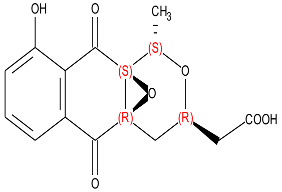	[B] Streptomyces rosa var. notoensis os-3966 (ATCC 31135, FERM-p 2209)
Napyradiomycin A	2328	-0.380	-39.627	3	C25 H30 Cl2 O5	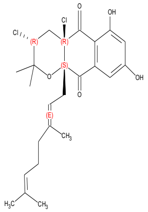	[B] Chainia rubra mg 802-af1; Streptomyces sp.
Nanaomycin betaE	2320	-0.387	-33.224	3	C16 H16 O6	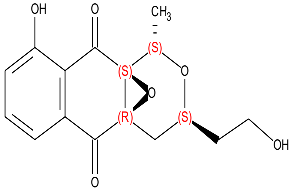	[B] Streptomyces sp. om-173 (FERM-p 6509)
Helminthosporin	10426	-0.390	-20.264	3	C15 H10 O5	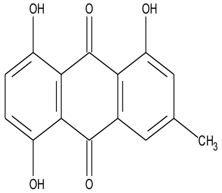	[F] mangrove endophytic fungus #2240

Nanaomycin aE	4550	-0.392	-34.993	3	C17 H16 O7	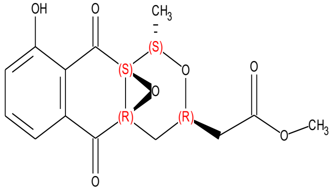	[B] Streptomyces sp. om-173 (FERM-p 6509)
Napyradiomycin B3	2331	-0.392	-40.809	3	[illegible]5 H29 Br1 Cl2 O5	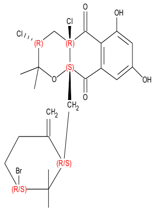	[B] Chainia rubra mg802-af1
1,3,4,5-Tetrahydroxy-2-methylanthraquinone	8849	-0.394	-28.797	3	C15 H10 O6	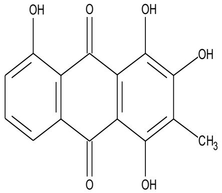	[Pl] Ventilago calyculata; [F] Penicillium islandicum
1,4,5-Trihydroxy-7-methoxy-3-methylanthraquinone	15391	-0.396	-22.262	3	C16 H12 O6	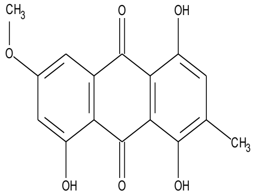	as a glycoside 10 roots of Cassia nodosaZ89 (Leguminosae). Thomson
Amylocyanine	1198	-0.402	-87.116	1	[illegible]20 H23 N3 O12	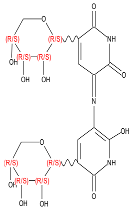	[B] Streptomyces coelicolor

Napyradiomycin C1	2332	-0.407	-36.317	3	C25 H28 Cl2 O5	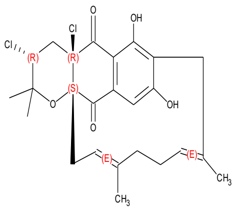	[B] Chainia rubra mg802-af1	
1,4,5-Trihydroxy-6-methylanthraquinone	20155	-0.413	-18.250	3	C15 H10 O5	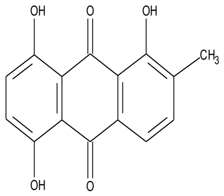	[synthetic]	
1,4,5,7-Tetrahydroxy-2-(1-hydroxypropyl)anthraquinone	15392	-0.419	-37.209	3	C17 H14 O7	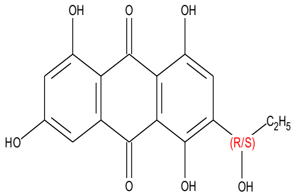		
Fusarnaphthoquinone B	38690	-0.423	-34.017	2	C15 H16 O5	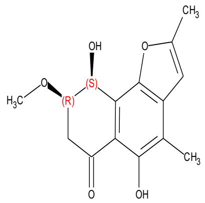	[F] Fusarium spp. PSU-F14 and PSU-F135	1
1,4,7,8-Tetrahydroxy-2-methylanthraquinone	16825	-0.424	-32.701	3	C15 H10 O6	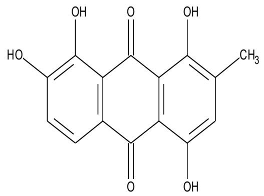	[F] Penicillium islandicum	

Frenolicin	1635	-0.427	-37.968	3	C18 H18 O7	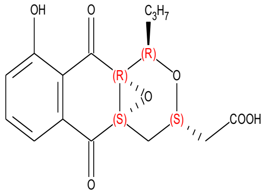	[B] Streptomyces fradiae
1,5-Dihydroxy-2-methoxy-anthraquinone	20119	-0.431	-26.311	3	C15 H10 O5	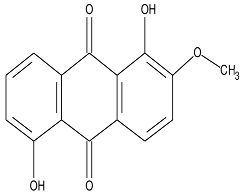	[synthetic]
Phosphatoquinone-A	23209	-0.437	-33.723	2	C21 H24 O5	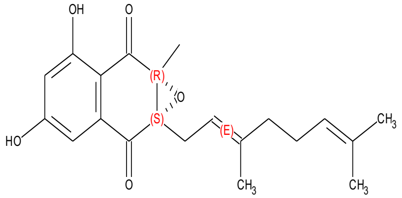	[B] Streptomyces sp. TA-0363
Nanaomycin aB	4549	-0.450	-39.793	3	C17 H18 O7	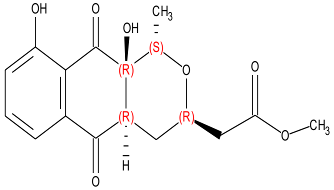	[B] Streptomyces sp. om-173 (FERM-p 6509)
Napyradiomycin B1	2329	-0.451	-41.639	3	C25 H29 Cl3 O5	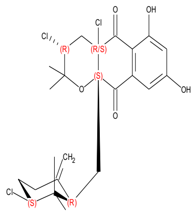	[B] Streptomyces sp.; Chainia rubra mg802-af1

(+)-Rugulosin	4895	-0.452	-62.709	4	C30 H22 O10	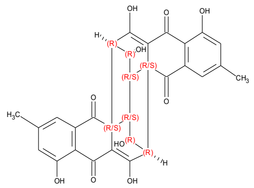	[F] Penicillium rugulosum, P.variabile, P.tardum, P.wortmanni, Endothia parasitica, E.fluens, E.gyrosa, Sepedonium ampullosporum, Myrothecium verrucaria, P.brunneum
1,4,5-Trihydroxy-7-methoxyanthraquinone	20134	-0.454	-23.339	3	C15 H10 O6	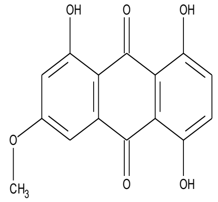	[synthetic]
1,8-Dihydroxy-3,5,7-trimethoxy-2-methylanthraquinone	15438	-0.461	-32.221	3	C18 H16 O7	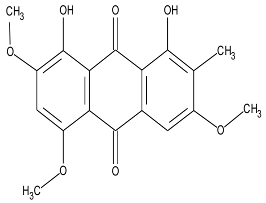	
1,3,8-Trihydroxy-2-(1-hydroxyhexyl)-6-methoxyanthraquinone	2964	-0.473	-0.186	3	C21 H22 O7	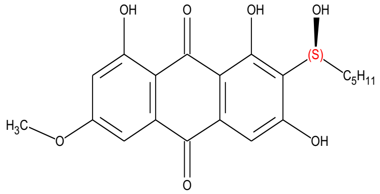	[L] Solorina crocea
Erythroglaucin	4265	-0.536	-23.480	3	C16 H12 O6	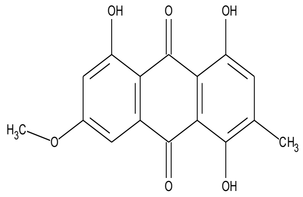	[L] [F] Aspergillus ruber ifo 6004, Asp.glaucus, Asp.cristatus (Eurotium cristatum), Xanthoria elegans

(-)-Rubroskyrin	2846	-0.554	-61.842	3	C30 H22 O12	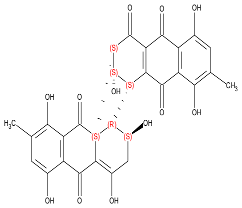	[F] Penicillium islandicum, P. rugulosum
2-n-Butyl-1,4,5,8-tetrahydroxy-anthraquinone	20126	-0.556	-18.647	3	C18 H16 O6	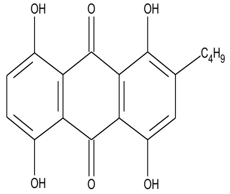	[synthetic]
1,4,5,8-Tetrahydroxy-2,6-dimethylanthraquinone	9836	-0.574	-17.791	3	C16 H12 O6	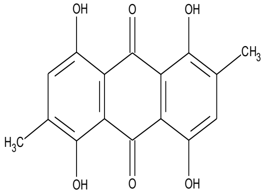	[F]
1,2,4,5,6-Pentahydroxy-7-hydroxymethylanthraquinone	9830	-0.578	-29.726	3	C15 H10 O8	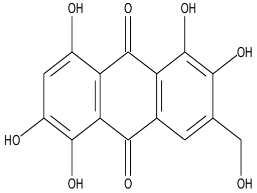	[F] Aspergillus nidulans
Nanaomycin-B	2322	-0.579	-27.671	3	C16 H16 O7	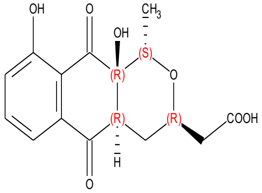	[B] Streptomyces rosa-notoensis

Julichrome Q3,3	1918	-0.585	-87.021	3	C38 H38 O16	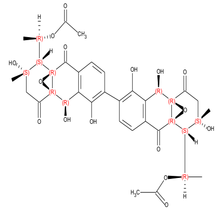	[B] Streptomyces shiodaensis	1
4-Hydroxydihydronorjavanicin	40043	-0.603	-39.515	2	C14 H16 O6	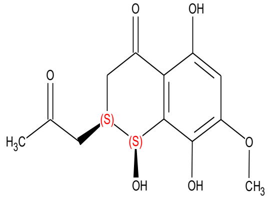	[F] endophytic Fusarium sp. BCC14842	1
1,4,5,8-Tetrahydroxy-2-methylanthraquinone	3938	-0.604	-18.153	3	C15 H10 O6	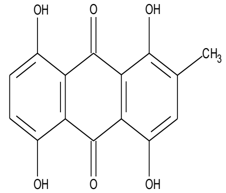	[F] Helminthosporium spp, Curruldria spp, Drechslera spp, Cercospora cari	
Verdoskyrin	14020	-0.608	-20.847	3	C15 H11 N1 O6	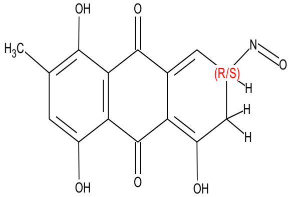	[F] Penicillium islandicum	
1,3,5,6,7,8-Hexahydroxy-2-methylanthraquinone	9884	-0.638	-35.484	3	C15 H10 O8	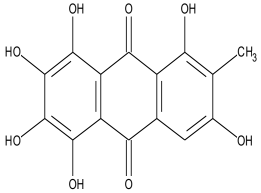	[F]	

2,3-Dihydro,5,8-dihydroxy-6-methoxy-2-hydroxymethyl-3-(2-hydroxypropyl)-1,4-naphthoquinone	17629	-0.643	-44.220	2	C15 H18 O7	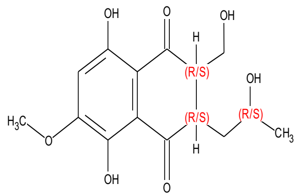	[F] Fusarium solani, Nectria haematococca (fungus)	
Quinone C	6694	-0.651	-37.537	2	C51 H74 O3	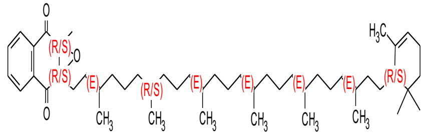	[B] Nocardia brasiliensis	
Quinone B	6693	-0.670	-35.067	2	C51 H76 O3	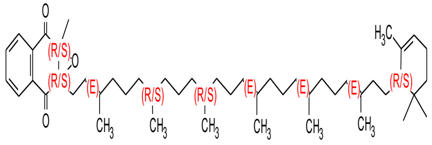	[B] Nocardia brasiliensis	
(3R, 4aS, 5R, 10aR)-5-Hydroxydihydrofusarubin A	40045	-0.679	-39.491	3	C15 H18 O7	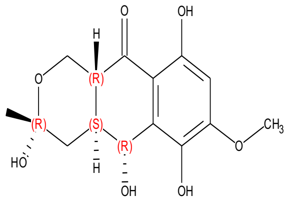	[F] endophytic Fusarium sp. BCC14842	
5-Hydroxy-3-methoxydihydrofusarubin A	40047	-0.696	-33.431	3	C16 H20 O7	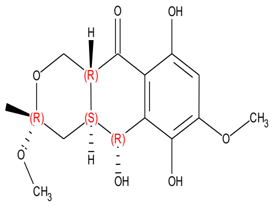	[F] endophytic Fusarium sp. BCC14842	1

Ascoquinone-A	14608	-0.795	-9.565	2	C30 H18 O15	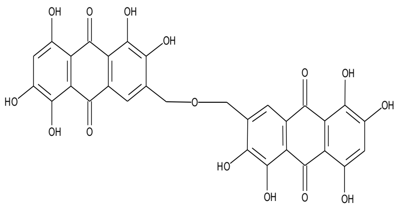	[F] Aspergillus nidulans spore pigment	
Dihydrofusarubin B	4235	-0.822	-37.629	3	C15 H16 O7	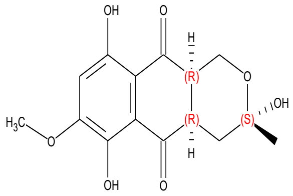	[F] Fusarium solani	
3-O-Ethyldihydrofusarubin B	4036	-0.833	-35.637	3	C17 H20 O7	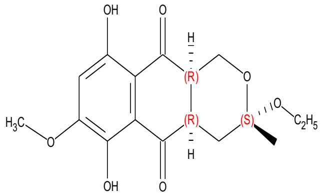	[F] Nectria haematococca F. marhi	
5-Hydroxydihydrofusarubin C	40044	-0.836	-38.643	3	C15 H18 O7	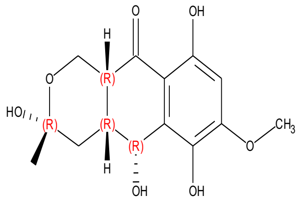	[F] endophytic Fusarium sp. BCC14842	1
(-)-Flavoskyrin	1590	-0.864	-68.254	3	C30 H24 O10	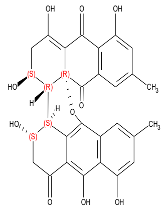	[F] Penicillium islandicum	

(3R, 4aS, 5S, 10aR)-5-Hydroxydihydrofusarubin D	40046	-0.885	-39.491	3	C15 H18 O7	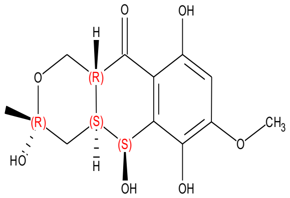	[F] endophytic Fusarium sp. BCC14842	1
3-O-Ethyldihydrofusarubin A	4035	-0.904	-31.816	3	C17 H20 O7	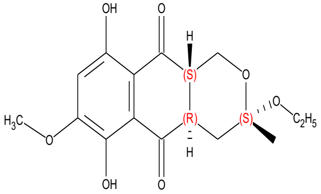	[F] Fusarium solani	
Dihydrofusarubin A	8248	-0.911	-34.873	3	C15 H16 O7	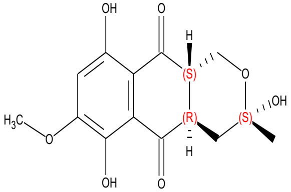	[F] Fusarium solani	
Stemphyltoxin IV	3526	-0.976	-36.403	4	C20 H12 O7	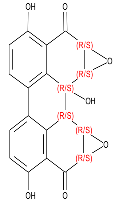	[F] Stemphylium botryosum var. lactucum	
3-O-Methyldihydrofusarubin A	4039	-1.001	-33.434	3	C16 H18 O7	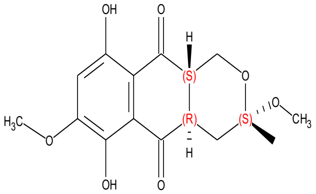	[F] Fusarium martii	

Altertoxin II	256	-1.008	-41.941	4	C20 H14 O6	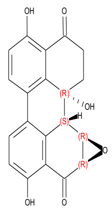	[F] Stemphylium botryosum var. lactucum, Alternaria tenuis, A. mali
Altertoxin I	7082	-1.094	-37.338	4	C20 H16 O6	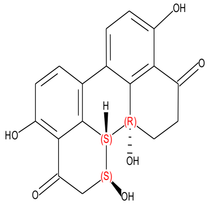	[F] Alternaria tenuis, A. mali; A. alternata
Granaticin B	1752	-1.144	-72.421	4	C28 H30 O12	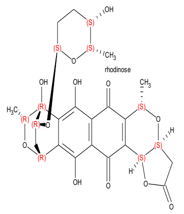	[B] Streptomyces spiroverticillatus N-9940 (FERM-p 2330), S.violaceoruber
Stemphyltoxin I	3524	-1.298	-40.153	4	C20 H14 O7	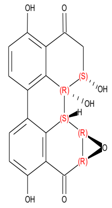	[F] Stemphylium botryosum var. lactucum
Pradimicin M	8584	-1.382	-74.938	4	C24 H16 O10	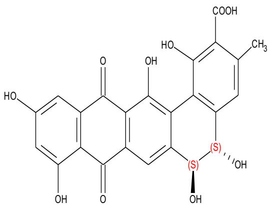	[B] blocked mutant of Actinomadura hibisca p157-2, Actinomadura verrucosospora subsp. neohibisca E-40
